# Taxonomy notes and new occurrence data of four species of atyid shrimp (Crustacea: Decapoda: Atyidae) in Vietnam, all described from China

**DOI:** 10.3897/BDJ.9.e70289

**Published:** 2021-09-27

**Authors:** Do Van Tu, Kristina von Rintelen, Werner Klotz, Le Hung Anh, Tran Anh Tuan, Dang Van Dong, Phan Thi Yen, Nguyen Tong Cuong, Hoang Ngoc Khac, Phan Doan Dang, Thomas von Rintelen

**Affiliations:** 1 Institute of Ecology and Biological Resources, Vietnam Academy of Science and Technology, 18 Hoang Quoc Viet, Cau Giay, Ha Noi, Vietnam Institute of Ecology and Biological Resources, Vietnam Academy of Science and Technology 18 Hoang Quoc Viet, Cau Giay, Ha Noi Vietnam; 2 Graduate University of Science and Technology, Vietnam Academy of Science and Technology, 18 Hoang Quoc Viet, Cau Giay, Ha Noi, Vietnam Graduate University of Science and Technology, Vietnam Academy of Science and Technology 18 Hoang Quoc Viet, Cau Giay, Ha Noi Vietnam; 3 Museum für Naturkunde, Leibniz Institute for Evolution and Biodiversity Science, Berlin, Germany Museum für Naturkunde, Leibniz Institute for Evolution and Biodiversity Science Berlin Germany; 4 Medizinische Universität Innsbruck, Wiesenweg 1, Austria Medizinische Universität Innsbruck Wiesenweg 1 Austria; 5 Hung Vuong University, Nong Trang Ward - Viet Tri City, Vietnam Hung Vuong University Nong Trang Ward - Viet Tri City Vietnam; 6 Department of Natural Resources Management, Hanoi University of Natural Resources and Environment, 41A Phu Dien, North-Tu Liem, Hanoi, Vietnam Department of Natural Resources Management, Hanoi University of Natural Resources and Environment 41A Phu Dien, North-Tu Liem, Hanoi Vietnam; 7 Institute of Tropical Biology, Vietnam Academy of Science and Technology, Ho Chi Minh, Vietnam Institute of Tropical Biology, Vietnam Academy of Science and Technology Ho Chi Minh Vietnam

**Keywords:** freshwater fauna, *
Caridina
*, *
Neocaridina
*, taxonomy, distribution

## Abstract

**Background:**

Freshwater shrimp of the family Atyidae De Haan, 1849 have been studied in Vietnam for more than a century. A total of 24 species of atyid shrimps from the genera *Caridina* H. Milne Edwards, 1837, *Neocaridina* Kubo, 1938, *Atyopsis* Chace, 1983 have been recorded from Vietnam. With 22 species, the majority are from the genus *Caridina*. In 2013, Karge and Klotz mentioned the occurrence of four yet undescribed species belonging to *Paracaridina* Liang, Guo & Tang, 1999 in Vietnam without taxonomic details.

In general, studies of freshwater atyids in Vietnam are limited and most Vietnamese taxa await a taxonomic revision. The available data do not fully reflect their estimated species diversity in the country and distribution data are deficient. Here, we focus on four species of atyid shrimps from two genera, viz. *Caridinacantonensis* Yu, 1938, *C.lanceifrons* Yu, 1936, *C.serrata* Stimpson, 1860 and *Neocaridinapalmata* (Shen, 1948), all described from China and have been reported to occur in Vietnam. The previous reports on the occurrence of these species in Vietnam are largely unreliable due to taxonomic confusion. To contribute to the knowledge of these taxa, we provide the first verified distribution records of the four species in the country with some taxonomic remarks.

**New information:**

This study shows the first taxonomically-verified distribution data of four atyid shrimp species originally described from China, but also reported from Vietnam, albeit under various species names and, in some cases, erroneously. These data allow the first meaningful discussion of the distribution in light of the reproductive strategy of these four species and, in conjunction with the taxonomic remarks, will contribute to the knowledge of these taxa. As a result of this research and data from previous studies, we now consider all four species as widespread and non-endemic, but land-locked (with a complete freshwater life cycle). In Vietnam, all four species are confined to the northern half of the country. Beyond Vietnam, we provide the first records for *Caridinalanceifrons* from southern Thailand, which suggests a major sampling gap in Indochina.

## Introduction

Freshwater shrimps of the family Atyidae (De Haan 1849) have a global distribution in the tropics and subtropics. Atyids are old (von Rintelen et al. 2012) and diverse with ca. 440 species (De Grave et al. 2015). The highest diversity of atyids is found in Southeast Asia (here including southern China) with more than 210 species in 13 genera (De Grave et al. 2008). Atyid shrimps are, alongside palaemonids, the only diverse group of shrimps in freshwater (De Grave et al. 2015) and play an important ecological role as primary consumers and aid in detritus decomposition (Dudgeon and Wu 1999, Crowl et al. 2001, Williams 2002, Yam and Dudgeon 2006).

Most recent taxonomic research on atyids – traditional and integrative – has focused on Southeast Asia, but the atyid fauna of Indochina including Vietnam has been largely neglected. Virtually all of the few studies in this region have been conducted in Vietnam and all are purely taxonomic. At present, 24 described atyid species from four genera, including *Caridina* Milne Edwards, 1837 (Milne Edwards 1837), *Paracaridina* Liang, Guo & Tang, 1999 (Liang et al. 1999), *Neocaridina* Kubo, 1938 (Kubo 1938) and *Atyopsis* Chace, 1983 (Chace 1983), have been recorded from Vietnam (Bouvier 1904, Bouvier 1905, Bouvier 1919, Bouvier 1925, Dang 1967, Dang 1975, Dang et al. 1980, Cai 1996, Cai et al. 1999, Nguyen 1999, Li and Liang 2002, Nguyen 2003, Dang and Do 2007a, Dang and Do 2007b, Dang and Do 2008, Do and Dang 2010, Dang and Ho 2012, Karge and Klotz 2013, Do et al. 2020, Do et al. 2021). The majority (22 species) come from the genus *Caridina* (Karge and Klotz 2013, Do et al. 2020, Do et al. 2021). The number of species is likely going to increase when more studies are done. However, the taxonomic status of all species described by Vietnamese taxonomists is hard to verify, as all types have been lost due to lack of care (the species described by Dang (1975), Dang et al. (1980), Dang and Do (2007a), Do and Dang (2010)).

The taxonomic confusion in atyids in general and Vietnamese taxa, in particular, does not only make it difficult to obtain accurate estimates of species diversity, but also prevents the use of existing data for biogeographic or evolutionary studies, as species distribution cannot be reliably assessed.

Four species, *Caridinacantonensis* Yu, 1938, *C.lanceifrons* Yu, 1936, *C.serrata* Stimpson, 1860 and *Neocaridinapalmata* (Shen, 1948), were originally described from China (Stimpson 1860, Shen 1948, Yu 1936, Yu 1938). Today, these are known to be widely distributed in China (Liang 2004). Vietnamese authors published taxonomic studies on the atyid shrimps of Vietnam including descriptions of new species, such as *Caridinaflavilineata* Dang, 1975, *C.vietriensis* Dang & Do, 2007, *C.pseudoflavilineata* Do & Dang, 2010 and *Caridinadenticulatavietnamensis* Dang, 1967. These authors also recorded *Caridinacantonensis* and *C.serrata* from Vietnam (Dang 1975, Dang et al. 1980, Dang and Do 2007a, Dang and Do 2007b, Dang and Ho 2012). However, Cai (1996) synonymised *Caridinadenticulatavietnamensis* with *Neocaridinapalmatapalmata* (Shen, 1948), Li and Liang (2002) and Liang (2004) considered *Caridinaflavilineata* Dang, 1975 as junior synonyms of *C.lanceifrons* Yu, 1936 (Li and Liang 2002, Liang 2004). Dang and Ho (2012) resurrected *C.flavilineata* and *N.vietnamensis* as distinct species compared to the species described from China. Based on the description and the figures in Dang (1975), Dang et al. (1980), Dang and Do (2007a), Do and Dang (2010), we found that three species of the genus *Caridina* (*C.flavilineata*, *C.vietriensis* and *C.pseudoflavilineata*) are similar to *C.lanceifrons* Yu, 1936, while *Neocaridinavietnamensis* is hardly distinguishable from *N.palmata* (Shen, 1948). In order to contribute to the taxonomic debate on the atyid shrimp fauna of Vietnam and China, only a proper revision could really solve it. A sampling campaign was conducted to cover as many locations in Vietnam as possible, especially in the northern part of the country. Our paper aims to address the relevant taxonomic issues and provide new occurrence data in Vietnam of four species of atyid shrimps originally described from China.

## Materials and methods

We collected atyid specimens in different habitats including rivers, streams, lakes and caves from the mainland to islands throughout Vietnam between 2003 and 2020. The collected sites were partially chosen for their accessibility (Fig. 1, Suppl. material 1). The collecting methods included catching by hand-nets both during the daytime and at night. At each site, at least 10 specimens were collected. To document their live colouration, specimens were then photographed with a Nikon D5600 Digital Camera and afterwards preserved in 90% ethanol. During the field surveys, other activities included observing living animals in their natural habitats, recording coordinates and photographing habitats. Additionally, we interviewed local people on their personal knowledge of the shrimp fauna to gain further information on species composition and distribution in local streams and rivers.

All collected samples were identified to species level, based on existing descriptions. The specimens were dissected and observed by using a Leica MZ 12 stereomicroscope. Voucher specimens are deposited in the Institute of Ecology and Biological Resources (IEBR), Vietnam Academy of Science and Technology (VAST), Ha Noi, Vietnam, the Museum für Naturkunde (MfN), Berlin, Germany (collection codens: ZMB), Ha Noi University of Science (HUS) and Oxford University Museum of Natural History (OUMNH). In addition to the data of the collected samples in Vietnam, we used records of the four species from China and other areas from the collection of Museum für Naturkunde (MfN), Berlin, Germany and the literature. For the latter, we only used records that could be georeferenced with reasonable accuracy and precision (< ca. 1 km radius across point locality) from authors who are experts on atyids. Based on these criteria, all records from Shen (1948), Zheng (1989), Cai and Yuan (1996), Cai and Ng (1999), Cai (2014) have been included. Liang (2004) contains a number of additional localities, but we have refrained from including them as they could not be georeferenced and the maps are not precise enough. The maps for *Caridinacantonensis* and *Neocaridinapalmata* are thus not complete for China.

In the description, all terminology used follows Chace (1997). The abbreviation cl is used for carapace length, measured from the postorbital margin to the posterior median margin of the carapace in mm. The rostral formula, used in the present study, is defined as: number of dorsal teeth on the carapace posterior to the orbital margin + the number of dorsal teeth on the rostrum anterior to the orbital margin/number of ventral teeth of the rostrum.

## Taxon treatments

### Caridina cantonensis

Yu, 1938

00D4BBA3-05D7-5EB9-9EE6-B5942CE5B847

Caridina cantonensis Yu, 1938: 290, figs. 7–8. [Type locality: Qing’ao Village, Nan’ao County, Guangdong Province, China; neotype designation by Cai and Ng (1999)].
Caridina mutata Cai & Ng, 1999: 1624; figs. 12–13. [Type locality: mountain stream near Fangcheng Town, Fangcheng County, Guangxi Province, China]

#### Materials

- **Type status:**Other material. **Occurrence:** catalogNumber: ZMB 30253; recordedBy: Do Van Tu; disposition: in collection; **Taxon:** scientificName: *Caridinacantonensis* Yu, 1938; kingdom: Animal; phylum: Arthropoda; class: Malacostraca; order: Decapoda; family: Atyidae; genus: Caridina; specificEpithet: *cantonensis*; taxonRank: Species; scientificNameAuthorship: Yu, 1938; vernacularName: atyid shrimp; nomenclaturalCode: ICZN; taxonomicStatus: accepted; **Location:** island: Cu Lao Cham; country: Vietnam; countryCode: VN; stateProvince: Quang Nam; county: Hoi An; locality: Stream near Bai Huong, Cu Lao Cham Island; decimalLatitude: 15.93944; decimalLongitude: 108.52; geodeticDatum: WGS84; georeferenceProtocol: GPS; **Identification:** identifiedBy: Do Van Tu; dateIdentified: 2020; **Event:** samplingProtocol: hand net; eventDate: 2017-5-9; year: 2017; month: 5; day: 9; **Record Level:** type: PhysicalObject; institutionCode: ZMB; collectionCode: ZMB; basisOfRecord: PreservedSpecimen
- **Type status:**Other material. **Occurrence:** catalogNumber: ZMB 30309; recordedBy: Do Van Tu; disposition: in collection; **Taxon:** scientificName: *Caridinacantonensis* Yu, 1938; kingdom: Animal; phylum: Arthropoda; class: Malacostraca; order: Decapoda; family: Atyidae; genus: Caridina; specificEpithet: *cantonensis*; taxonRank: Species; scientificNameAuthorship: Yu, 1938; vernacularName: atyid shrimp; nomenclaturalCode: ICZN; taxonomicStatus: accepted; **Location:** island: Cu Lao Cham; country: Vietnam; countryCode: VN; stateProvince: Quang Nam; county: Hoi An; locality: Stream near on the road to Bai Huong, Cu Lao Cham Island; decimalLatitude: 15.94417; decimalLongitude: 108.5125; geodeticDatum: WGS84; georeferenceProtocol: GPS; **Identification:** identifiedBy: Do Van Tu; dateIdentified: 2020; **Event:** samplingProtocol: hand net; eventDate: 2017-5-9; year: 2017; month: 5; day: 9; **Record Level:** type: PhysicalObject; institutionCode: ZMB; collectionCode: ZMB; basisOfRecord: PreservedSpecimen
- **Type status:**Other material. **Occurrence:** catalogNumber: OUMNH 201307016; recordedBy: Werner & Maria Klotz; disposition: in collection; **Taxon:** scientificName: *Caridinacantonensis* Yu, 1938; kingdom: Animal; phylum: Arthropoda; class: Malacostraca; order: Decapoda; family: Atyidae; genus: Caridina; specificEpithet: *cantonensis*; taxonRank: Species; scientificNameAuthorship: Yu, 1938; vernacularName: atyid shrimp; nomenclaturalCode: ICZN; taxonomicStatus: accepted; **Location:** country: China; countryCode: CN; stateProvince: Guangdong; locality: Conghua, small stream and trickle; decimalLatitude: 23.575; decimalLongitude: 113.4374; geodeticDatum: WGS84; georeferenceProtocol: GPS; **Identification:** identifiedBy: Do Van Tu; dateIdentified: 2020; **Event:** samplingProtocol: hand net; eventDate: 2012-4-1; year: 2012; month: 4; day: 1; **Record Level:** type: PhysicalObject; institutionCode: OUMNH; collectionCode: OUMNH; basisOfRecord: PreservedSpecimen
- **Type status:**Other material. **Occurrence:** catalogNumber: ZMB 28041; recordedBy: Werner Klotz, Yixiong Cai, Chris Lukhaup; disposition: in collection; **Taxon:** scientificName: *Caridinacantonensis* Yu, 1938; kingdom: Animal; phylum: Arthropoda; class: Malacostraca; order: Decapoda; family: Atyidae; genus: Caridina; specificEpithet: *cantonensis*; taxonRank: Species; scientificNameAuthorship: Yu, 1938; vernacularName: atyid shrimp; nomenclaturalCode: ICZN; taxonomicStatus: accepted; **Location:** country: China; countryCode: CN; stateProvince: Guangdong; locality: Near Quing Yuan city; decimalLatitude: 23.56641; decimalLongitude: 113.17765; geodeticDatum: WGS84; georeferenceProtocol: GPS; **Identification:** identifiedBy: Do Van Tu; dateIdentified: 2020; **Event:** samplingProtocol: hand net; eventDate: 2012-4-1; year: 2012; month: 4; day: 1; **Record Level:** type: PhysicalObject; institutionCode: ZMB; collectionCode: ZMB; basisOfRecord: PreservedSpecimen
- **Type status:**Other material. **Occurrence:** catalogNumber: ZMB 31788; recordedBy: Zheng, M.Q. (1989); disposition: in collection; **Taxon:** scientificName: *Caridinacantonensis* Yu, 1938; kingdom: Animal; phylum: Arthropoda; class: Malacostraca; order: Decapoda; family: Atyidae; genus: Caridina; specificEpithet: *cantonensis*; taxonRank: Species; scientificNameAuthorship: Yu, 1938; vernacularName: atyid shrimp; nomenclaturalCode: ICZN; taxonomicStatus: accepted; **Location:** island: Hong Kong; country: China; countryCode: CN; stateProvince: ﻿Hong Kong; locality: Kau Sai Chau Island, stream at E shore (Stream "B"); decimalLatitude: 22.3575; decimalLongitude: 114.3186; geodeticDatum: WGS84; georeferenceProtocol: GPS; **Identification:** identifiedBy: Do Van Tu; dateIdentified: 2020; **Event:** samplingProtocol: hand net; eventDate: 2015-5-28; year: 2015; month: 5; day: 28; **Record Level:** type: PhysicalObject; institutionCode: ZMB; collectionCode: ZMB; basisOfRecord: PreservedSpecimen
- **Type status:**Other material. **Occurrence:** catalogNumber: ZMB 31790; recordedBy: Julia Chan, Thomas von Rintelen; disposition: in collection; **Taxon:** scientificName: *Caridinacantonensis* Yu, 1938; kingdom: Animal; phylum: Arthropoda; class: Malacostraca; order: Decapoda; family: Atyidae; genus: Caridina; specificEpithet: *cantonensis*; taxonRank: Species; scientificNameAuthorship: Yu, 1938; vernacularName: atyid shrimp; nomenclaturalCode: ICZN; taxonomicStatus: accepted; **Location:** island: Hong Kong; country: China; countryCode: CN; stateProvince: ﻿Hong Kong; locality: Kau Sai Chau Island, stream at E shore (Stream "A"); decimalLatitude: 22.359; decimalLongitude: 114.3185; geodeticDatum: WGS84; georeferenceProtocol: GPS; **Identification:** identifiedBy: Do Van Tu; dateIdentified: 2020; **Event:** samplingProtocol: hand net; eventDate: 2015-5-20; year: 2015; month: 5; day: 20; **Record Level:** type: PhysicalObject; institutionCode: ZMB; collectionCode: ZMB; basisOfRecord: PreservedSpecimen
- **Type status:**Other material. **Occurrence:** catalogNumber: ZMB 31793; recordedBy: Julia Chan; disposition: in collection; **Taxon:** scientificName: *Caridinacantonensis* Yu, 1938; kingdom: Animal; phylum: Arthropoda; class: Malacostraca; order: Decapoda; family: Atyidae; genus: Caridina; specificEpithet: *cantonensis*; taxonRank: Species; scientificNameAuthorship: Yu, 1938; vernacularName: atyid shrimp; nomenclaturalCode: ICZN; taxonomicStatus: accepted; **Location:** island: Hong Kong; country: China; countryCode: CN; stateProvince: ﻿Hong Kong; locality: Kau Sai Chau Island, stream at E shore (Stream "C" upstream); decimalLatitude: 22.3535; decimalLongitude: 114.3223; geodeticDatum: WGS84; georeferenceProtocol: GPS; **Identification:** identifiedBy: Do Van Tu; dateIdentified: 2020; **Event:** samplingProtocol: hand net; eventDate: 2015-5-28; year: 2015; month: 5; day: 28; **Record Level:** type: PhysicalObject; institutionCode: ZMB; collectionCode: ZMB; basisOfRecord: PreservedSpecimen
- **Type status:**Other material. **Occurrence:** catalogNumber: ZMB 32144; recordedBy: Werner & Maria Klotz; disposition: in collection; **Taxon:** scientificName: *Caridinacantonensis* Yu, 1938; kingdom: Animal; phylum: Arthropoda; class: Malacostraca; order: Decapoda; family: Atyidae; genus: Caridina; specificEpithet: *cantonensis*; taxonRank: Species; scientificNameAuthorship: Yu, 1938; vernacularName: atyid shrimp; nomenclaturalCode: ICZN; taxonomicStatus: accepted; **Location:** country: China; countryCode: CN; stateProvince: Guangdong; locality: Spring near Lixi Town (near Ying De); decimalLatitude: 23.9059; decimalLongitude: 113.2457; geodeticDatum: WGS84; georeferenceProtocol: GPS; **Identification:** identifiedBy: Do Van Tu; dateIdentified: 2020; **Event:** samplingProtocol: hand net; eventDate: 2010-3-18; year: 2010; month: 3; day: 18; **Record Level:** type: PhysicalObject; institutionCode: ZMB; collectionCode: ZMB; basisOfRecord: PreservedSpecimen
- **Type status:**Other material. **Occurrence:** catalogNumber: ZMB 32145; recordedBy: Werner & Maria Klotz; disposition: in collection; **Taxon:** scientificName: *Caridinacantonensis* Yu, 1938; kingdom: Animal; phylum: Arthropoda; class: Malacostraca; order: Decapoda; family: Atyidae; genus: Caridina; specificEpithet: *cantonensis*; taxonRank: Species; scientificNameAuthorship: Yu, 1938; vernacularName: atyid shrimp; nomenclaturalCode: ICZN; taxonomicStatus: accepted; **Location:** country: China; countryCode: CN; stateProvince: Guangdong; locality: Conghua, small stream and trickle; decimalLatitude: 23.575; decimalLongitude: 113.4374; geodeticDatum: WGS84; georeferenceProtocol: GPS; **Identification:** identifiedBy: Do Van Tu; dateIdentified: 2020; **Event:** samplingProtocol: hand net; eventDate: 2010-3-21; year: 2010; month: 3; day: 21; **Record Level:** type: PhysicalObject; institutionCode: ZMB; collectionCode: ZMB; basisOfRecord: PreservedSpecimen
- **Type status:**Other material. **Occurrence:** catalogNumber: ZMB 32146; recordedBy: Werner & Maria Klotz; disposition: in collection; **Taxon:** scientificName: *Caridinacantonensis* Yu, 1938; kingdom: Animal; phylum: Arthropoda; class: Malacostraca; order: Decapoda; family: Atyidae; genus: Caridina; specificEpithet: *cantonensis*; taxonRank: Species; scientificNameAuthorship: Yu, 1938; vernacularName: atyid shrimp; nomenclaturalCode: ICZN; taxonomicStatus: accepted; **Location:** country: China; countryCode: CN; stateProvince: Guangdong; locality: Small mountain stream near Lixi Town (near Ying De); decimalLatitude: 23.9067; decimalLongitude: 113.2521; geodeticDatum: WGS84; georeferenceProtocol: GPS; **Identification:** identifiedBy: Do Van Tu; dateIdentified: 2020; **Event:** samplingProtocol: hand net; eventDate: 2010-3-19; year: 2010; month: 3; day: 19; **Record Level:** type: PhysicalObject; institutionCode: ZMB; collectionCode: ZMB; basisOfRecord: PreservedSpecimen
- **Type status:**Other material. **Occurrence:** catalogNumber: ZMB 32147; recordedBy: Werner & Maria Klotz; disposition: in collection; **Taxon:** scientificName: *Caridinacantonensis* Yu, 1938; kingdom: Animal; phylum: Arthropoda; class: Malacostraca; order: Decapoda; family: Atyidae; genus: Caridina; specificEpithet: *cantonensis*; taxonRank: Species; scientificNameAuthorship: Yu, 1938; vernacularName: atyid shrimp; nomenclaturalCode: ICZN; taxonomicStatus: accepted; **Location:** country: China; countryCode: CN; stateProvince: Guangdong; locality: Small mountain stream near Lixi Town (near Ying De); decimalLatitude: 23.9095; decimalLongitude: 113.2597; geodeticDatum: WGS84; georeferenceProtocol: GPS; **Identification:** identifiedBy: Do Van Tu; dateIdentified: 2020; **Event:** samplingProtocol: hand net; eventDate: 2010-3-19; year: 2010; month: 3; day: 19; **Record Level:** type: PhysicalObject; institutionCode: ZMB; collectionCode: ZMB; basisOfRecord: PreservedSpecimen
- **Type status:**Other material. **Occurrence:** catalogNumber: ZMB 32183; recordedBy: Werner Klotz; disposition: in collection; **Taxon:** scientificName: *Caridinacantonensis* Yu, 1938; kingdom: Animal; phylum: Arthropoda; class: Malacostraca; order: Decapoda; family: Atyidae; genus: Caridina; specificEpithet: *cantonensis*; taxonRank: Species; scientificNameAuthorship: Yu, 1938; vernacularName: atyid shrimp; nomenclaturalCode: ICZN; taxonomicStatus: accepted; **Location:** country: China; countryCode: CN; stateProvince: Guangdong; locality: Henquin island, Zhuhai, San Die Quan spring; decimalLatitude: 22.11369; decimalLongitude: 113.50278; geodeticDatum: WGS84; georeferenceProtocol: GPS; **Identification:** identifiedBy: Do Van Tu; dateIdentified: 2020; **Event:** samplingProtocol: hand net; eventDate: 2009-4-5; year: 2009; month: 4; day: 5; **Record Level:** type: PhysicalObject; institutionCode: ZMB; collectionCode: ZMB; basisOfRecord: PreservedSpecimen
- **Type status:**Other material. **Occurrence:** catalogNumber: ZMB 32185; recordedBy: Werner Klotz; disposition: in collection; **Taxon:** scientificName: *Caridinacantonensis* Yu, 1938; kingdom: Animal; phylum: Arthropoda; class: Malacostraca; order: Decapoda; family: Atyidae; genus: Caridina; specificEpithet: *cantonensis*; taxonRank: Species; scientificNameAuthorship: Yu, 1938; vernacularName: atyid shrimp; nomenclaturalCode: ICZN; taxonomicStatus: accepted; **Location:** island: Hong Kong; country: China; countryCode: CN; stateProvince: ﻿Hong Kong; locality: Hongkong Island, Lung Hang Stream; decimalLatitude: 22.4035; decimalLongitude: 114.3209; geodeticDatum: WGS84; georeferenceProtocol: GPS; **Identification:** identifiedBy: Do Van Tu; dateIdentified: 2020; **Event:** samplingProtocol: hand net; eventDate: 2009-4-7; year: 2009; month: 4; day: 7; **Record Level:** type: PhysicalObject; institutionCode: ZMB; collectionCode: ZMB; basisOfRecord: PreservedSpecimen
- **Type status:**Other material. **Occurrence:** catalogNumber: ZMB 32186; recordedBy: pet trade; disposition: in collection; **Taxon:** scientificName: *Caridinacantonensis* Yu, 1938; kingdom: Animal; phylum: Arthropoda; class: Malacostraca; order: Decapoda; family: Atyidae; genus: Caridina; specificEpithet: *cantonensis*; taxonRank: Species; scientificNameAuthorship: Yu, 1938; vernacularName: atyid shrimp; nomenclaturalCode: ICZN; taxonomicStatus: accepted; **Location:** island: Hong Kong; country: China; countryCode: CN; stateProvince: ﻿Hong Kong; decimalLatitude: 22.38333; decimalLongitude: 114.1; geodeticDatum: WGS84; georeferenceProtocol: GPS; **Identification:** identifiedBy: Do Van Tu; dateIdentified: 2020; **Event:** samplingProtocol: hand net; eventDate: 2009-8-1; year: 2009; month: 8; day: 1; **Record Level:** type: PhysicalObject; institutionCode: ZMB; collectionCode: ZMB; basisOfRecord: PreservedSpecimen
- **Type status:**Other material. **Occurrence:** catalogNumber: ZMB 32192; recordedBy: Werner Klotz; disposition: in collection; **Taxon:** scientificName: *Caridinacantonensis* Yu, 1938; kingdom: Animal; phylum: Arthropoda; class: Malacostraca; order: Decapoda; family: Atyidae; genus: Caridina; specificEpithet: *cantonensis*; taxonRank: Species; scientificNameAuthorship: Yu, 1938; vernacularName: atyid shrimp; nomenclaturalCode: ICZN; taxonomicStatus: accepted; **Location:** island: Hong Kong; country: China; countryCode: CN; stateProvince: ﻿Hong Kong; locality: Lantau Island, Stream at Pui O; decimalLatitude: 22.2485; decimalLongitude: 113.9828; geodeticDatum: WGS84; georeferenceProtocol: GPS; **Identification:** identifiedBy: Do Van Tu; dateIdentified: 2020; **Event:** samplingProtocol: hand net; eventDate: 2009-4-9; year: 2009; month: 4; day: 9; **Record Level:** type: PhysicalObject; institutionCode: ZMB; collectionCode: ZMB; basisOfRecord: PreservedSpecimen
- **Type status:**Other material. **Occurrence:** catalogNumber: ZMB 32195; recordedBy: Werner Klotz; disposition: in collection; **Taxon:** scientificName: *Caridinacantonensis* Yu, 1938; kingdom: Animal; phylum: Arthropoda; class: Malacostraca; order: Decapoda; family: Atyidae; genus: Caridina; specificEpithet: *cantonensis*; taxonRank: Species; scientificNameAuthorship: Yu, 1938; vernacularName: atyid shrimp; nomenclaturalCode: ICZN; taxonomicStatus: accepted; **Location:** island: Hong Kong; country: China; countryCode: CN; stateProvince: ﻿Hong Kong; locality: Stream at Ha Miu Tin; decimalLatitude: 22.5033; decimalLongitude: 114.2643; geodeticDatum: WGS84; georeferenceProtocol: GPS; **Identification:** identifiedBy: Do Van Tu; dateIdentified: 2020; **Event:** samplingProtocol: hand net; eventDate: 2009-4-8; year: 2009; month: 4; day: 8; **Record Level:** type: PhysicalObject; institutionCode: ZMB; collectionCode: ZMB; basisOfRecord: PreservedSpecimen
- **Type status:**Other material. **Occurrence:** catalogNumber: ZMB 32196; recordedBy: Werner Klotz; disposition: in collection; **Taxon:** scientificName: *Caridinacantonensis* Yu, 1938; kingdom: Animal; phylum: Arthropoda; class: Malacostraca; order: Decapoda; family: Atyidae; genus: Caridina; specificEpithet: *cantonensis*; taxonRank: Species; scientificNameAuthorship: Yu, 1938; vernacularName: atyid shrimp; nomenclaturalCode: ICZN; taxonomicStatus: accepted; **Location:** island: Hong Kong; country: China; countryCode: CN; stateProvince: ﻿Hong Kong; locality: Stream near Sheung Miu Tin; decimalLatitude: 22.5023; decimalLongitude: 114.26; geodeticDatum: WGS84; georeferenceProtocol: GPS; **Identification:** identifiedBy: Do Van Tu; dateIdentified: 2020; **Event:** samplingProtocol: hand net; eventDate: 2019-4-8; year: 2019; month: 4; day: 8; **Record Level:** type: PhysicalObject; institutionCode: ZMB; collectionCode: ZMB; basisOfRecord: PreservedSpecimen
- **Type status:**Other material. **Occurrence:** catalogNumber: ZMB 32307; recordedBy: Andreas Karge; disposition: in collection; **Taxon:** scientificName: *Caridinacantonensis* Yu, 1938; kingdom: Animal; phylum: Arthropoda; class: Malacostraca; order: Decapoda; family: Atyidae; genus: Caridina; specificEpithet: *cantonensis*; taxonRank: Species; scientificNameAuthorship: Yu, 1938; vernacularName: atyid shrimp; nomenclaturalCode: ICZN; taxonomicStatus: accepted; **Location:** island: Hong Kong; country: China; countryCode: CN; stateProvince: ﻿Hong Kong; locality: Tai Mo Shan; decimalLatitude: 22.409; decimalLongitude: 114.1372; geodeticDatum: WGS84; georeferenceProtocol: GPS; **Identification:** identifiedBy: Do Van Tu; dateIdentified: 2020; **Event:** samplingProtocol: hand net; eventDate: 2010-3-23; year: 2010; month: 3; day: 23; **Record Level:** type: PhysicalObject; institutionCode: ZMB; collectionCode: ZMB; basisOfRecord: PreservedSpecimen
- **Type status:**Other material. **Occurrence:** catalogNumber: ZMB 29690; recordedBy: Chris Lukhaup; disposition: in collection; **Taxon:** scientificName: *Caridinacantonensis* Yu, 1938; kingdom: Animal; phylum: Arthropoda; class: Malacostraca; order: Decapoda; family: Atyidae; genus: Caridina; specificEpithet: *cantonensis*; taxonRank: Species; scientificNameAuthorship: Yu, 1938; vernacularName: atyid shrimp; nomenclaturalCode: ICZN; taxonomicStatus: accepted; **Location:** country: China; countryCode: CN; stateProvince: Guangdong; locality: W of Shenzen (ca. 1h); decimalLatitude: 22.74862; decimalLongitude: 114.06034; geodeticDatum: WGS84; georeferenceProtocol: GPS; **Identification:** identifiedBy: Do Van Tu; dateIdentified: 2020; **Event:** samplingProtocol: hand net; eventDate: 2015-4-15; year: 2015; month: 4; day: 15; **Record Level:** type: PhysicalObject; institutionCode: ZMB; collectionCode: ZMB; basisOfRecord: PreservedSpecimen
- **Type status:**Other material. **Occurrence:** catalogNumber: ZMB 29756; recordedBy: Chris Lukhaup; disposition: in collection; **Taxon:** scientificName: *Caridinacantonensis* Yu, 1938; kingdom: Animal; phylum: Arthropoda; class: Malacostraca; order: Decapoda; family: Atyidae; genus: Caridina; specificEpithet: *cantonensis*; taxonRank: Species; scientificNameAuthorship: Yu, 1938; vernacularName: atyid shrimp; nomenclaturalCode: ICZN; taxonomicStatus: accepted; **Location:** country: China; countryCode: CN; stateProvince: Guangdong; locality: Shenzen city boundary; decimalLatitude: 22.74929; decimalLongitude: 114.06058; geodeticDatum: WGS84; georeferenceProtocol: GPS; **Identification:** identifiedBy: Do Van Tu; dateIdentified: 2020; **Event:** samplingProtocol: hand net; eventDate: 2015-4-15; year: 2015; month: 4; day: 15; **Record Level:** type: PhysicalObject; institutionCode: ZMB; collectionCode: ZMB; basisOfRecord: PreservedSpecimen

#### Description

**Material examined for description**. *Caridinacantonensis* Yu, 1938: 7 males, cl 3.5–4.1, 4 females, cl 4.0–4.8 (ZMB 30253), Vietnam: Quang Nam Province, Hoi An City, Cu Lao Cham Island, a stream near Bai Huong, 15°56'22''N 108°31'12''E, coll. Do Van Tu, 9 May 2017; 3 males, cl 3.6–4.0, 2 females, cl 4.2–4.4 (ZMB 30309), Vietnam: Quang Nam Province, Hoi An City, Cu Lao Cham Island, a small stream ca. 3 km to Bai Huong, 15°56'39''N 108°30'45'' E, coll. Do Van Tu, 9 May 2017.

**Cephalothorax and cephalic appendages.** Carapace length 3.5–4.8 mm. Rostrum straight, slightly bent downwards, reaching to middle or the end of second segment of antennular peduncle, 0.38–0.46 (median 0.43) times as long as carapace, rostral formula: 4–5+9–12/3–5 (n = 10), teeth normal (Fig. 2a). Suborbital angle acute, completely fused with antennal spine; pterygostomian margin rounded (Fig. 2a). Eyes well developed with globular cornea, anterior end reaching to 0.7 times length of basal segment of antennular peduncle (Fig. 2a). Antennular peduncle 0.54–0.67 (median 0.60) times as long as carapace; basal segment 1.38–1.83 (median 1.57) times as long as second segment, second segment 1.4–2.0 (median 1.55) times as long as third segment (Fig. 2b). Stylocerite reaching almost to middle (0.4 times length) of second segment of antennular peduncle (Fig. 2b). Scaphocerite reaching beyond distal end of antennular peduncle, 3.0–4.0 (median 3.46) times as long as wide (Fig. 2a, c).

**Abdominal somites, telson and uropods.** Sixth abdominal somite 0.5–0.6 (median 0.57) times length of carapace, 1.50–2.0 (median 1.67) times as long as fifth abdominal somite, 0.86–1.05 (median 1.0) times length of telson. Telson length 2.1–2.75 (median 2.44) times as long as proximal width, distal margin triangular, terminating in a short median projection, with 5–6 pairs of dorsal spiniform setae and one pair of dorso-subdistal spiniform setae; distal end with 4 pairs of spiniform setae, lateral pair slightly shorter than intermediate pairs (Fig. 2d). Pre-anal carina low, with few setae, lacking a tooth or spine. Uropodal diaeresis with 18 movable spiniform setae, outermost one slightly shorter than lateral angle (Fig. 2e).

**Pereiopods.** Epipod present on first four pereiopods. First pereiopod short, robust, reaching beyond end of basal segment of antennular peduncle; chela 2.0–2.2 (median 2.0) times as long as wide, 1.33–1.67 (median 1.42) times length of carpus; tips of fingers rounded, without hooks; dactylus slightly longer than palm, 1.0–1.1 (median 1.1) times as long as palm in males and shorter than palm, 0.8–0.86 (median 0.83) times as long as palm in females; carpus excavated anteriorly, 1.5–1.88 (median 1.65) times as long as wide, 0.67–0.8 (median 0.8) times length of merus; merus 2.14–3.0 (median 2.5) times as long as wide, longer than ischium (Fig. 3a). Second pereiopod long, slender, reaching to distal end of antennular peduncle; chela 2.22–2.63 (median 2.42) times as long as wide, 0.66–0.8 (median 0.73) times length of carpus; tips of fingers rounded, without hooks; dactylus 1.22–1.30 (median 1.26) times as long as palm; carpus 4.5–5.3 (median 4.67) times as long as wide, 1.09–1.19 (median 1.14) times as long as merus; merus 4.18–5.0 (median 4.65) times as long as wide, longer than ischium (Fig. 3b). Third pereiopod slender, reaching beyond end of scaphocerite by its dactylus, terminating in one claw, with four to five accessory spiniform setae on flexor margin, dactylus 2.83–3.6 (median 3.2) times as long as wide (terminal claw and spiniform setae on flexor margin included), propodus 9.0–10.9 (median 9.42) times as long as wide, 3.5–4.47 (median 3.88) times as long as dactylus; carpus 4.25–5.0 (median 4.75) times as long as wide, 0.61–0.67 (median 0.67) times as long as propodus, 0.45–0.63 (median 0.5) times as long as merus; merus 5.71–6.8 (median 6.19) times as long as wide, bearing four strong, movable spiniform setae on posterior margin of outer surface; ischium with one movable spiniform seta (Fig. 3c). Fifth pereiopod slender, reaching to end of second segment of antennular peduncle, dactylus 3.2–3.6 (median 3.2) times as long as wide (terminal claw and spiniform setae on flexor margin included), terminating in one large claw, with 25–28 spiniform setae on flexor margin; propodus 13.7–14.69 (median 13.71) times as long as wide, 3.13–5.33 (median 5.22) times length of dactylus; carpus 4.2–4.75 (median 4.50) times as long as wide, 0.48–0.56 (median 0.55) times as long as propodus, 0.4–0.51 (median 0.46) times as long as merus; merus 5.45–6.0 (median 6.0) times as long as wide, bearing 3–4 strong, movable spiniform setae on posterior margin of outer surface (Fig. 3d).

**Pleopods.** Endopod of male first pleopod extending to 0.6 times length of exopod, rectangular in shape, anterior part folded backwards, 2.0–2.73 (median 2.53) times as long as proximal width, inner margin concave, outer margin concave or slightly straight, rounded distally, long plumose setae on outer and distal margins, medium-length setae on inner margin; with appendix interna exceeding terminal margin of endopod by 0.3 of its length (Fig. 3e). Appendix masculina of male second pleopod slender, sub-cylindrical, reaching to proximal 0.5 times endopod length, 4.7 times as long as distal width, finger-shaped, slightly widened distally with some short spiniform setae on outer surface and some long spiniform setae on distal surface; appendix interna at middle of appendix masculina, narrow, small, extending about 0.5 times length of appendix masculina (Fig. 3f).

**Figure 4.**
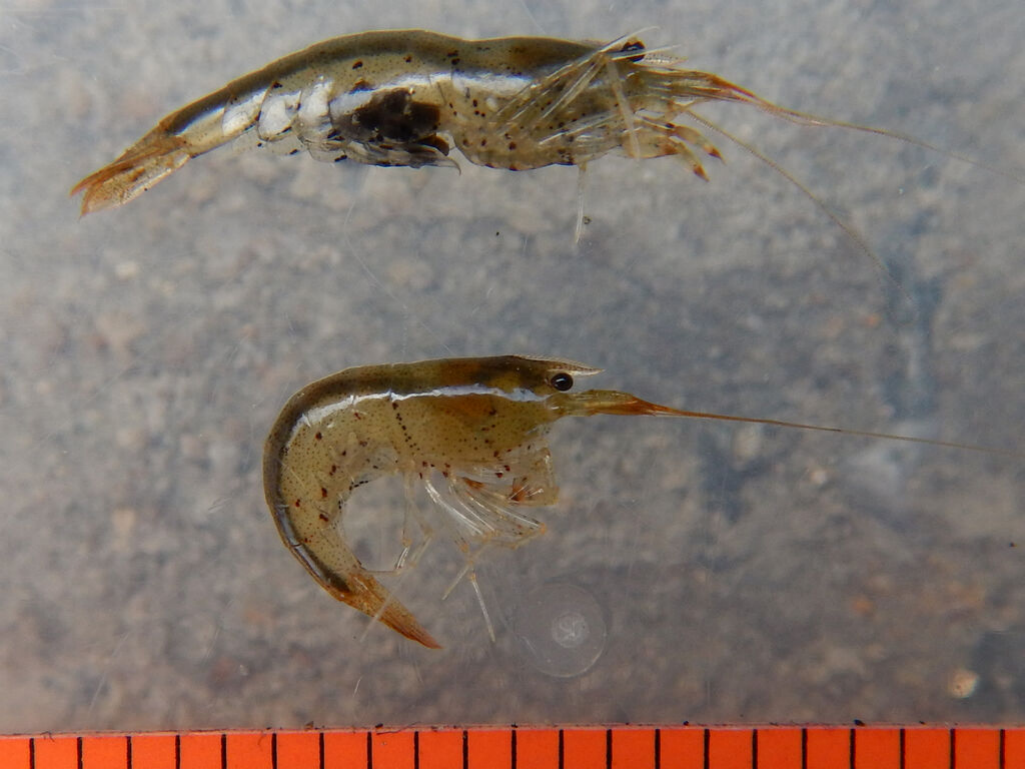
Live colouration of *Caridinacantonensis* Yu, 1938, collected on Cu Lao Cham Island, Hoi An City, Quang Nam Province.

**Colouration.** Body yellowish, few irregular small black spots present on ventrolateral parts of carapace and abdominal somites (Fig. 4).

#### Distribution

Cu Lao Cham, in north-central Vietnam (Fig. 1a). Guangdong, Guangxi and Hong Kong, China (Liang 2004).

#### Notes

Dang et al. (1980) recorded *C.cantonensis* from Chi Lang District, Lang Son Province, North Vietnam, near the border to China. We could neither find this species amongst our samples surrounding this area nor from many survey sites in the mainland of northern Vietnam. Our samples from Lang Son Province are not just a distinct species compared to *C.cantonensis*, especially in the anterior region of the endopod of male first pleopod (straight vs. folded backwards), but belong to an undescribed species. As this species has not been known from China until now, a description would be out of the focus of this paper. However, we discovered *C.cantonensis* at Cu Lao Cham Island off the central coast of Vietnam (Fig. 1a). The population in Vietnam showed no differences when compared to the original description (Yu 1938) and the later re-descriptions in Cai and Ng (1999) and Liang (2004), except the lower number of spiniform setae on flexor margin of the dactylus of the fifth pereiopod (25–28 vs. 38–45, Fig. 3c, cf. Yu (1938): fig. 8h). In China, this species is known from Guangdong and Guangxi Provinces and from Hong Kong Island. It is only found in streams and, based on egg-size (Table 1), it can be considered as land-locked (Dudgeon 1987). In our study, this species was only found on one small island in central Vietnam, far from the type locality (Fig. 1). An introduction of this species, for example, along with fish fry from fish farming, cannot be ruled out.

### Caridina lanceifrons

Yu, 1936

7EF1B844-B684-5777-A5D5-0518D27AC513

Caridina lanceifrons Yu, 1936: 89, figs. 4–7. [Type locality: near the lighthouse at Hai-kiu-sche in the salt water; neotype locality: pond near Qiongshan City, Haikou, Hainan, China (designation by Cai (2014)).
Caridina lanceifrons — Liang and Zheng (1988): 15; Zheng (1989): 8; Liang and Zhou (1993): 231; Liang et al. (1993): 41; Li (1997): 454; Li and Liang (2002): 709; Cai (2014): 217–220, figs. 6–7.
Caridina flavilineata Dang, 1975: 70–71, fig. 5. [Type locality: Nam Ha = Ha Nam and Nam Dinh Provinces, northern Vietnam]; Dang and Do (2008): 8–10, fig. 5; Dang and Ho (2012): 154–156, fig. 53.
Caridina vietriensis Dang & Do, 2007: 9–10, figs. 7–8. [Type locality: Viet Tri City, Phu Tho Province, Vietnam].
Caridina pseudoflavilineata Do & Dang, 2010: 31–34, figs. 3–4. [Type locality: Hai Van mountain pass, Thua Thien Hue Province, Vietnam].

#### Materials

- **Type status:**Other material. **Occurrence:** catalogNumber: ZMB 32411 or 32312; recordedBy: Marco Endruweit, Wang Jing; disposition: in collection; **Taxon:** scientificName: *Caridinalanceifrons* Yu, 1936; kingdom: Animal; phylum: Arthropoda; class: Malacostraca; order: Decapoda; family: Atyidae; genus: Caridina; specificEpithet: *lanceifrons*; taxonRank: Species; scientificNameAuthorship: Yu, 1936; vernacularName: atyid shrimp; nomenclaturalCode: ICZN; taxonomicStatus: accepted; **Location:** country: Vietnam; countryCode: VN; stateProvince: Thai Nguyen; county: Pho Yen; municipality: Dac Son; locality: Cau River, along Road TL 261 Pho Yen - Đại Từ, crossing road from right - left, flowing out off the Nui Coc reservoir, Red River basin, Eastface of Tao Dao National Park; decimalLatitude: 21.42178; decimalLongitude: 105.83862; geodeticDatum: WGS84; georeferenceProtocol: GPS; **Identification:** identifiedBy: Do Van Tu; dateIdentified: 2020; **Event:** samplingProtocol: hand net; eventDate: 2012-4-8; year: 2012; month: 4; day: 8; **Record Level:** type: PhysicalObject; institutionCode: ZMB; collectionCode: ZMB; basisOfRecord: PreservedSpecimen
- **Type status:**Other material. **Occurrence:** catalogNumber: ZMB; recordedBy: Andreas Karge; disposition: in collection; **Taxon:** scientificName: *Caridinalanceifrons* Yu, 1936; kingdom: Animal; phylum: Arthropoda; class: Malacostraca; order: Decapoda; family: Atyidae; genus: Caridina; specificEpithet: *lanceifrons*; taxonRank: Species; scientificNameAuthorship: Yu, 1936; vernacularName: atyid shrimp; nomenclaturalCode: ICZN; taxonomicStatus: accepted; **Location:** country: Vietnam; countryCode: VN; stateProvince: Quang Nam; county: Duy Xuyen; locality: My Son (Ruin City); decimalLatitude: 15.7634; decimalLongitude: 108.12458; geodeticDatum: WGS84; georeferenceProtocol: GPS; **Identification:** identifiedBy: Do Van Tu; dateIdentified: 2020; **Event:** samplingProtocol: hand net; eventDate: 2009-7-17; year: 2009; month: 7; day: 17; **Record Level:** type: PhysicalObject; institutionCode: ZMB; collectionCode: ZMB; basisOfRecord: PreservedSpecimen
- **Type status:**Other material. **Occurrence:** catalogNumber: ZMB 29493; recordedBy: Frank Köhler; disposition: in collection; **Taxon:** scientificName: *Caridinalanceifrons* Yu, 1936; kingdom: Animal; phylum: Arthropoda; class: Malacostraca; order: Decapoda; family: Atyidae; genus: Caridina; specificEpithet: *lanceifrons*; taxonRank: Species; scientificNameAuthorship: Yu, 1936; vernacularName: atyid shrimp; nomenclaturalCode: ICZN; taxonomicStatus: accepted; **Location:** country: Vietnam; countryCode: VN; stateProvince: Quang Tri; locality: Dakrông River near Dakrong, 20 km South of Dakrong bridge; decimalLatitude: 16.5735; decimalLongitude: 106.95983; geodeticDatum: WGS84; georeferenceProtocol: GPS; **Identification:** identifiedBy: Do Van Tu; dateIdentified: 2020; **Event:** samplingProtocol: hand net; eventDate: 2006-10-21; year: 2006; month: 10; day: 21; **Record Level:** type: PhysicalObject; institutionCode: ZMB; collectionCode: ZMB; basisOfRecord: PreservedSpecimen
- **Type status:**Other material. **Occurrence:** catalogNumber: ZMB 29494; recordedBy: Frank Köhler; disposition: in collection; **Taxon:** scientificName: *Caridinalanceifrons* Yu, 1936; kingdom: Animal; phylum: Arthropoda; class: Malacostraca; order: Decapoda; family: Atyidae; genus: Caridina; specificEpithet: *lanceifrons*; taxonRank: Species; scientificNameAuthorship: Yu, 1936; vernacularName: atyid shrimp; nomenclaturalCode: ICZN; taxonomicStatus: accepted; **Location:** country: Vietnam; countryCode: VN; stateProvince: Nghe An; locality: Highway 7 between Con Cuong and Laos; decimalLatitude: 19.215; decimalLongitude: 104.5475; geodeticDatum: WGS84; georeferenceProtocol: GPS; **Identification:** identifiedBy: Do Van Tu; dateIdentified: 2020; **Event:** samplingProtocol: hand net; eventDate: 2006-10-21; year: 2006; month: 10; day: 21; **Record Level:** type: PhysicalObject; institutionCode: ZMB; collectionCode: ZMB; basisOfRecord: PreservedSpecimen
- **Type status:**Other material. **Occurrence:** catalogNumber: ZMB 29495; recordedBy: Frank Köhler; disposition: in collection; **Taxon:** scientificName: *Caridinalanceifrons* Yu, 1936; kingdom: Animal; phylum: Arthropoda; class: Malacostraca; order: Decapoda; family: Atyidae; genus: Caridina; specificEpithet: *lanceifrons*; taxonRank: Species; scientificNameAuthorship: Yu, 1936; vernacularName: atyid shrimp; nomenclaturalCode: ICZN; taxonomicStatus: accepted; **Location:** country: Vietnam; countryCode: VN; stateProvince: Bac Kan; county: Cho Moi; municipality: Cao Ky; locality: River parallel highway Thai Nguyen - Bac Kan, 13km South of Bac Kan; decimalLatitude: 22.03498; decimalLongitude: 105.85088; geodeticDatum: WGS84; georeferenceProtocol: GPS; **Identification:** identifiedBy: Do Van Tu; dateIdentified: 2020; **Event:** samplingProtocol: hand net; eventDate: 2006-3-21; year: 2006; month: 3; day: 21; **Record Level:** type: PhysicalObject; institutionCode: ZMB; collectionCode: ZMB; basisOfRecord: PreservedSpecimen
- **Type status:**Other material. **Occurrence:** catalogNumber: ZMB 29496; recordedBy: Frank Köhler; disposition: in collection; **Taxon:** scientificName: *Caridinalanceifrons* Yu, 1936; kingdom: Animal; phylum: Arthropoda; class: Malacostraca; order: Decapoda; family: Atyidae; genus: Caridina; specificEpithet: *lanceifrons*; taxonRank: Species; scientificNameAuthorship: Yu, 1936; vernacularName: atyid shrimp; nomenclaturalCode: ICZN; taxonomicStatus: accepted; **Location:** country: Vietnam; countryCode: VN; stateProvince: Quang Binh; county: Minh Hoa; decimalLatitude: 17.87483; decimalLongitude: 105.867; geodeticDatum: WGS84; georeferenceProtocol: GPS; **Identification:** identifiedBy: Do Van Tu; dateIdentified: 2020; **Event:** samplingProtocol: hand net; eventDate: 2006-10-21; year: 2006; month: 10; day: 21; **Record Level:** type: PhysicalObject; institutionCode: ZMB; collectionCode: ZMB; basisOfRecord: PreservedSpecimen
- **Type status:**Other material. **Occurrence:** catalogNumber: ZMB 29638; recordedBy: Do Van Tu; disposition: in collection; **Taxon:** scientificName: *Caridinalanceifrons* Yu, 1936; kingdom: Animal; phylum: Arthropoda; class: Malacostraca; order: Decapoda; family: Atyidae; genus: Caridina; specificEpithet: *lanceifrons*; taxonRank: Species; scientificNameAuthorship: Yu, 1936; vernacularName: atyid shrimp; nomenclaturalCode: ICZN; taxonomicStatus: accepted; **Location:** country: Vietnam; countryCode: VN; stateProvince: Hoa Binh; county: Da Bac; decimalLatitude: 20.88603; decimalLongitude: 105.15267; geodeticDatum: WGS84; georeferenceProtocol: GPS; **Identification:** identifiedBy: Do Van Tu; dateIdentified: 2020; **Event:** samplingProtocol: hand net; eventDate: 2015-3-14; year: 2015; month: 3; day: 14; **Record Level:** type: PhysicalObject; institutionCode: ZMB; collectionCode: ZMB; basisOfRecord: PreservedSpecimen
- **Type status:**Other material. **Occurrence:** catalogNumber: ZMB 29640; recordedBy: Do Van Tu; disposition: in collection; **Taxon:** scientificName: *Caridinalanceifrons* Yu, 1936; kingdom: Animal; phylum: Arthropoda; class: Malacostraca; order: Decapoda; family: Atyidae; genus: Caridina; specificEpithet: *lanceifrons*; taxonRank: Species; scientificNameAuthorship: Yu, 1936; vernacularName: atyid shrimp; nomenclaturalCode: ICZN; taxonomicStatus: accepted; **Location:** country: Vietnam; countryCode: VN; stateProvince: Ha Giang; county: Quan Binh; municipality: Bang Lang; decimalLatitude: 22.36506; decimalLongitude: 104.63511; geodeticDatum: WGS84; georeferenceProtocol: GPS; **Identification:** identifiedBy: Do Van Tu; dateIdentified: 2020; **Event:** samplingProtocol: hand net; eventDate: 2012-11-16; year: 2012; month: 11; day: 16; **Record Level:** type: PhysicalObject; institutionCode: ZMB; collectionCode: ZMB; basisOfRecord: PreservedSpecimen
- **Type status:**Other material. **Occurrence:** catalogNumber: ZMB 29646; recordedBy: Do Van Tu; disposition: in collection; **Taxon:** scientificName: *Caridinalanceifrons* Yu, 1936; kingdom: Animal; phylum: Arthropoda; class: Malacostraca; order: Decapoda; family: Atyidae; genus: Caridina; specificEpithet: *lanceifrons*; taxonRank: Species; scientificNameAuthorship: Yu, 1936; vernacularName: atyid shrimp; nomenclaturalCode: ICZN; taxonomicStatus: accepted; **Location:** country: Vietnam; countryCode: VN; stateProvince: Yen Bai; county: Van Chan; decimalLatitude: 21.47856; decimalLongitude: 104.73222; geodeticDatum: WGS84; georeferenceProtocol: GPS; **Identification:** identifiedBy: Do Van Tu; dateIdentified: 2020; **Event:** samplingProtocol: hand net; eventDate: 2012-11-20; year: 2012; month: 11; day: 20; **Record Level:** type: PhysicalObject; institutionCode: ZMB; collectionCode: ZMB; basisOfRecord: PreservedSpecimen
- **Type status:**Other material. **Occurrence:** catalogNumber: ZMB 29647; recordedBy: Do Van Tu; disposition: in collection; **Taxon:** scientificName: *Caridinalanceifrons* Yu, 1936; kingdom: Animal; phylum: Arthropoda; class: Malacostraca; order: Decapoda; family: Atyidae; genus: Caridina; specificEpithet: *lanceifrons*; taxonRank: Species; scientificNameAuthorship: Yu, 1936; vernacularName: atyid shrimp; nomenclaturalCode: ICZN; taxonomicStatus: accepted; **Location:** country: Vietnam; countryCode: VN; stateProvince: Quang Binh; locality: Stream near km 28, Phong Nha-Ke Bang National Park; decimalLatitude: 17.49628; decimalLongitude: 106.29108; geodeticDatum: WGS84; georeferenceProtocol: GPS; **Identification:** identifiedBy: Do Van Tu; dateIdentified: 2020; **Event:** samplingProtocol: hand net; eventDate: 2014-4-22; year: 2014; month: 4; day: 22; **Record Level:** type: PhysicalObject; institutionCode: ZMB; collectionCode: ZMB; basisOfRecord: PreservedSpecimen
- **Type status:**Other material. **Occurrence:** catalogNumber: ZMB 29652; recordedBy: Do Van Tu; disposition: in collection; **Taxon:** scientificName: *Caridinalanceifrons* Yu, 1936; kingdom: Animal; phylum: Arthropoda; class: Malacostraca; order: Decapoda; family: Atyidae; genus: Caridina; specificEpithet: *lanceifrons*; taxonRank: Species; scientificNameAuthorship: Yu, 1936; vernacularName: atyid shrimp; nomenclaturalCode: ICZN; taxonomicStatus: accepted; **Location:** country: Vietnam; countryCode: VN; stateProvince: Thai Binh; locality: Thanh Tan; decimalLatitude: 20.43108; decimalLongitude: 106.43853; geodeticDatum: WGS84; georeferenceProtocol: GPS; **Identification:** identifiedBy: Do Van Tu; dateIdentified: 2020; **Event:** samplingProtocol: hand net; eventDate: 2010-6-5; year: 2010; month: 6; day: 5; **Record Level:** type: PhysicalObject; institutionCode: ZMB; collectionCode: ZMB; basisOfRecord: PreservedSpecimen
- **Type status:**Other material. **Occurrence:** catalogNumber: ZMB 29658; recordedBy: Jens Kühne; disposition: in collection; **Taxon:** scientificName: *Caridinalanceifrons* Yu, 1936; kingdom: Animal; phylum: Arthropoda; class: Malacostraca; order: Decapoda; family: Atyidae; genus: Caridina; specificEpithet: *lanceifrons*; taxonRank: Species; scientificNameAuthorship: Yu, 1936; vernacularName: atyid shrimp; nomenclaturalCode: ICZN; taxonomicStatus: accepted; **Location:** country: Vietnam; countryCode: VN; stateProvince: Hoa Binh; locality: Village 15 km from Hoa Binh, small creek, downstream; decimalLatitude: 20.90833; decimalLongitude: 105.33611; geodeticDatum: WGS84; georeferenceProtocol: GPS; **Identification:** identifiedBy: Do Van Tu; dateIdentified: 2020; **Event:** samplingProtocol: hand net; eventDate: 2015-7-6; year: 2015; month: 7; day: 6; **Record Level:** type: PhysicalObject; institutionCode: ZMB; collectionCode: ZMB; basisOfRecord: PreservedSpecimen
- **Type status:**Other material. **Occurrence:** catalogNumber: ZMB 29659; recordedBy: Jens Kühne; disposition: in collection; **Taxon:** scientificName: *Caridinalanceifrons* Yu, 1936; kingdom: Animal; phylum: Arthropoda; class: Malacostraca; order: Decapoda; family: Atyidae; genus: Caridina; specificEpithet: *lanceifrons*; taxonRank: Species; scientificNameAuthorship: Yu, 1936; vernacularName: atyid shrimp; nomenclaturalCode: ICZN; taxonomicStatus: accepted; **Location:** country: Vietnam; countryCode: VN; stateProvince: Hoa Binh; locality: Village 15 km from Hoa Binh, small creek, upstream; decimalLatitude: 20.90861; decimalLongitude: 105.33667; geodeticDatum: WGS84; georeferenceProtocol: GPS; **Identification:** identifiedBy: Do Van Tu; dateIdentified: 2020; **Event:** samplingProtocol: hand net; eventDate: 2015-7-6; year: 2015; month: 7; day: 6; **Record Level:** type: PhysicalObject; institutionCode: ZMB; collectionCode: ZMB; basisOfRecord: PreservedSpecimen
- **Type status:**Other material. **Occurrence:** catalogNumber: ZMB 29662; recordedBy: Jens Kühne; disposition: in collection; **Taxon:** scientificName: *Caridinalanceifrons* Yu, 1936; kingdom: Animal; phylum: Arthropoda; class: Malacostraca; order: Decapoda; family: Atyidae; genus: Caridina; specificEpithet: *lanceifrons*; taxonRank: Species; scientificNameAuthorship: Yu, 1936; vernacularName: atyid shrimp; nomenclaturalCode: ICZN; taxonomicStatus: accepted; **Location:** country: Vietnam; countryCode: VN; stateProvince: Hoa Binh; locality: Song Da Catchment, below Coffeeshop on the street No 6; decimalLatitude: 20.71278; decimalLongitude: 105.04194; geodeticDatum: WGS84; georeferenceProtocol: GPS; **Identification:** identifiedBy: Do Van Tu; dateIdentified: 2020; **Event:** samplingProtocol: hand net; eventDate: 2015-7-10; year: 2015; month: 7; day: 10; **Record Level:** type: PhysicalObject; institutionCode: ZMB; collectionCode: ZMB; basisOfRecord: PreservedSpecimen
- **Type status:**Other material. **Occurrence:** catalogNumber: ZMB 29758; recordedBy: Marco Endruweit, Wang Jing; disposition: in collection; **Taxon:** scientificName: *Caridinalanceifrons* Yu, 1936; kingdom: Animal; phylum: Arthropoda; class: Malacostraca; order: Decapoda; family: Atyidae; genus: Caridina; specificEpithet: *lanceifrons*; taxonRank: Species; scientificNameAuthorship: Yu, 1936; vernacularName: atyid shrimp; nomenclaturalCode: ICZN; taxonomicStatus: accepted; **Location:** country: Vietnam; countryCode: VN; stateProvince: Tuyen Quang; county: Son Duong; locality: Pho Day River (Song Pho Day) mainchannel, Lô-Gâm River subbasin, Red River basin, westface of Tam Dao National Park; decimalLatitude: 21.53797; decimalLongitude: 105.48437; geodeticDatum: WGS84; georeferenceProtocol: GPS; **Identification:** identifiedBy: Do Van Tu; dateIdentified: 2020; **Event:** samplingProtocol: hand net; eventDate: 2012-4-11; year: 2012; month: 4; day: 11; **Record Level:** type: PhysicalObject; institutionCode: ZMB; collectionCode: ZMB; basisOfRecord: PreservedSpecimen
- **Type status:**Other material. **Occurrence:** catalogNumber: ZMB 29761; recordedBy: Marco Endruweit, Wang Jing; disposition: in collection; **Taxon:** scientificName: *Caridinalanceifrons* Yu, 1936; kingdom: Animal; phylum: Arthropoda; class: Malacostraca; order: Decapoda; family: Atyidae; genus: Caridina; specificEpithet: *lanceifrons*; taxonRank: Species; scientificNameAuthorship: Yu, 1936; vernacularName: atyid shrimp; nomenclaturalCode: ICZN; taxonomicStatus: accepted; **Location:** country: Vietnam; countryCode: VN; stateProvince: Thai Nguyen; county: Pho Yen; decimalLatitude: 21.48103; decimalLongitude: 105.72652; geodeticDatum: WGS84; georeferenceProtocol: GPS; **Identification:** identifiedBy: Do Van Tu; dateIdentified: 2020; **Event:** samplingProtocol: hand net; eventDate: 2012-4-8; year: 2012; month: 4; day: 8; **Record Level:** type: PhysicalObject; institutionCode: ZMB; collectionCode: ZMB; basisOfRecord: PreservedSpecimen
- **Type status:**Other material. **Occurrence:** catalogNumber: ZMB 30228; recordedBy: Thomas von Rintelen, Do Van Tu; disposition: in collection; **Taxon:** scientificName: *Caridinalanceifrons* Yu, 1936; kingdom: Animal; phylum: Arthropoda; class: Malacostraca; order: Decapoda; family: Atyidae; genus: Caridina; specificEpithet: *lanceifrons*; taxonRank: Species; scientificNameAuthorship: Yu, 1936; vernacularName: atyid shrimp; nomenclaturalCode: ICZN; taxonomicStatus: accepted; **Location:** country: Vietnam; countryCode: VN; stateProvince: Ninh Binh; locality: River in limestone area Northeast of Cuc Phuong National Park; decimalLatitude: 20.27252; decimalLongitude: 105.91127; geodeticDatum: WGS84; georeferenceProtocol: GPS; **Identification:** identifiedBy: Do Van Tu; dateIdentified: 2020; **Event:** samplingProtocol: hand net; eventDate: 2017-3-4; year: 2017; month: 3; day: 4; **Record Level:** type: PhysicalObject; institutionCode: ZMB; collectionCode: ZMB; basisOfRecord: PreservedSpecimen
- **Type status:**Other material. **Occurrence:** catalogNumber: ZMB 30258; recordedBy: Do Van Tu; disposition: in collection; **Taxon:** scientificName: *Caridinalanceifrons* Yu, 1936; kingdom: Animal; phylum: Arthropoda; class: Malacostraca; order: Decapoda; family: Atyidae; genus: Caridina; specificEpithet: *lanceifrons*; taxonRank: Species; scientificNameAuthorship: Yu, 1936; vernacularName: atyid shrimp; nomenclaturalCode: ICZN; taxonomicStatus: accepted; **Location:** country: Vietnam; countryCode: VN; stateProvince: Hoa Binh; county: Kim Boi; locality: Bo; decimalLatitude: 20.66833; decimalLongitude: 105.53528; geodeticDatum: WGS84; georeferenceProtocol: GPS; **Identification:** identifiedBy: Do Van Tu; dateIdentified: 2020; **Event:** samplingProtocol: hand net; eventDate: 2017-4-23; year: 2017; month: 4; day: 23; **Record Level:** type: PhysicalObject; institutionCode: ZMB; collectionCode: ZMB; basisOfRecord: PreservedSpecimen
- **Type status:**Other material. **Occurrence:** catalogNumber: ZMB 30264; recordedBy: Do Van Tu; disposition: in collection; **Taxon:** scientificName: *Caridinalanceifrons* Yu, 1936; kingdom: Animal; phylum: Arthropoda; class: Malacostraca; order: Decapoda; family: Atyidae; genus: Caridina; specificEpithet: *lanceifrons*; taxonRank: Species; scientificNameAuthorship: Yu, 1936; vernacularName: atyid shrimp; nomenclaturalCode: ICZN; taxonomicStatus: accepted; **Location:** country: Vietnam; countryCode: VN; stateProvince: Da Nang; locality: Stream on Lang Van; decimalLatitude: 16.16778; decimalLongitude: 108.14222; geodeticDatum: WGS84; georeferenceProtocol: GPS; **Identification:** identifiedBy: Do Van Tu; dateIdentified: 2020; **Event:** samplingProtocol: hand net; eventDate: 2017-5-5; year: 2017; month: 5; day: 5; **Record Level:** type: PhysicalObject; institutionCode: ZMB; collectionCode: ZMB; basisOfRecord: PreservedSpecimen
- **Type status:**Other material. **Occurrence:** catalogNumber: ZMB 30266; recordedBy: Do Van Tu; disposition: in collection; **Taxon:** scientificName: *Caridinalanceifrons* Yu, 1936; kingdom: Animal; phylum: Arthropoda; class: Malacostraca; order: Decapoda; family: Atyidae; genus: Caridina; specificEpithet: *lanceifrons*; taxonRank: Species; scientificNameAuthorship: Yu, 1936; vernacularName: atyid shrimp; nomenclaturalCode: ICZN; taxonomicStatus: accepted; **Location:** country: Vietnam; countryCode: VN; stateProvince: Nghe An; locality: Khe Kem water fall, Pu Mat National Park; decimalLatitude: 18.97044; decimalLongitude: 104.80075; geodeticDatum: WGS84; georeferenceProtocol: GPS; **Identification:** identifiedBy: Do Van Tu; dateIdentified: 2020; **Event:** samplingProtocol: hand net; eventDate: 2017-5-18; year: 2017; month: 5; day: 18; **Record Level:** type: PhysicalObject; institutionCode: ZMB; collectionCode: ZMB; basisOfRecord: PreservedSpecimen
- **Type status:**Other material. **Occurrence:** catalogNumber: ZMB 30268; recordedBy: Le Hung Anh; disposition: in collection; **Taxon:** scientificName: *Caridinalanceifrons* Yu, 1936; kingdom: Animal; phylum: Arthropoda; class: Malacostraca; order: Decapoda; family: Atyidae; genus: Caridina; specificEpithet: *lanceifrons*; taxonRank: Species; scientificNameAuthorship: Yu, 1936; vernacularName: atyid shrimp; nomenclaturalCode: ICZN; taxonomicStatus: accepted; **Location:** country: Vietnam; countryCode: VN; stateProvince: Nghe An; county: Ky Son; municipality: Muong Xen; locality: Muong Xen; decimalLatitude: 19.28122; decimalLongitude: 104.42111; geodeticDatum: WGS84; georeferenceProtocol: GPS; **Identification:** identifiedBy: Do Van Tu; dateIdentified: 2020; **Event:** samplingProtocol: hand net; eventDate: 2017-3-9; year: 2017; month: 3; day: 9; **Record Level:** type: PhysicalObject; institutionCode: ZMB; collectionCode: ZMB; basisOfRecord: PreservedSpecimen
- **Type status:**Other material. **Occurrence:** catalogNumber: ZMB 30275; recordedBy: Do Van Tu; disposition: in collection; **Taxon:** scientificName: *Caridinalanceifrons* Yu, 1936; kingdom: Animal; phylum: Arthropoda; class: Malacostraca; order: Decapoda; family: Atyidae; genus: Caridina; specificEpithet: *lanceifrons*; taxonRank: Species; scientificNameAuthorship: Yu, 1936; vernacularName: atyid shrimp; nomenclaturalCode: ICZN; taxonomicStatus: accepted; **Location:** country: Vietnam; countryCode: VN; stateProvince: Quang Tri; county: Huong Hoa; municipality: Huong Linh; locality: Ku Vo; decimalLatitude: 16.72072; decimalLongitude: 106.86354; geodeticDatum: WGS84; georeferenceProtocol: GPS; **Identification:** identifiedBy: Do Van Tu; dateIdentified: 2020; **Event:** samplingProtocol: hand net; eventDate: 2017-6-25; year: 2017; month: 6; day: 25; **Record Level:** type: PhysicalObject; institutionCode: ZMB; collectionCode: ZMB; basisOfRecord: PreservedSpecimen
- **Type status:**Other material. **Occurrence:** catalogNumber: ZMB 30279; recordedBy: Nguyen Tong Cuong; disposition: in collection; **Taxon:** scientificName: *Caridinalanceifrons* Yu, 1936; kingdom: Animal; phylum: Arthropoda; class: Malacostraca; order: Decapoda; family: Atyidae; genus: Caridina; specificEpithet: *lanceifrons*; taxonRank: Species; scientificNameAuthorship: Yu, 1936; vernacularName: atyid shrimp; nomenclaturalCode: ICZN; taxonomicStatus: accepted; **Location:** country: Vietnam; countryCode: VN; stateProvince: Vinh Phuc; locality: Me Linh Station for Biodiversity; decimalLatitude: 21.38967; decimalLongitude: 105.71271; geodeticDatum: WGS84; georeferenceProtocol: GPS; **Identification:** identifiedBy: Do Van Tu; dateIdentified: 2020; **Event:** samplingProtocol: hand net; eventDate: 2017-3-16; year: 2017; month: 3; day: 16; **Record Level:** type: PhysicalObject; institutionCode: ZMB; collectionCode: ZMB; basisOfRecord: PreservedSpecimen
- **Type status:**Other material. **Occurrence:** catalogNumber: ZMB 30284; recordedBy: Do Van Tu; disposition: in collection; **Taxon:** scientificName: *Caridinalanceifrons* Yu, 1936; kingdom: Animal; phylum: Arthropoda; class: Malacostraca; order: Decapoda; family: Atyidae; genus: Caridina; specificEpithet: *lanceifrons*; taxonRank: Species; scientificNameAuthorship: Yu, 1936; vernacularName: atyid shrimp; nomenclaturalCode: ICZN; taxonomicStatus: accepted; **Location:** country: Vietnam; countryCode: VN; stateProvince: Hai Phong; county: Thuy Nguyen; locality: NT4; decimalLatitude: 20.97348; decimalLongitude: 106.75721; geodeticDatum: WGS84; georeferenceProtocol: GPS; **Identification:** identifiedBy: Do Van Tu; dateIdentified: 2020; **Event:** samplingProtocol: hand net; eventDate: 2017-1-1; year: 2017; month: 1; day: 1; **Record Level:** type: PhysicalObject; institutionCode: ZMB; collectionCode: ZMB; basisOfRecord: PreservedSpecimen
- **Type status:**Other material. **Occurrence:** catalogNumber: ZMB 30286; recordedBy: Do Van Tu; disposition: in collection; **Taxon:** scientificName: *Caridinalanceifrons* Yu, 1936; kingdom: Animal; phylum: Arthropoda; class: Malacostraca; order: Decapoda; family: Atyidae; genus: Caridina; specificEpithet: *lanceifrons*; taxonRank: Species; scientificNameAuthorship: Yu, 1936; vernacularName: atyid shrimp; nomenclaturalCode: ICZN; taxonomicStatus: accepted; **Location:** country: Vietnam; countryCode: VN; stateProvince: Quang Binh; county: Le Thuy; municipality: Kim Thuy; locality: Ho Rum; decimalLatitude: 16.99901; decimalLongitude: 106.64663; geodeticDatum: WGS84; georeferenceProtocol: GPS; **Identification:** identifiedBy: Do Van Tu; dateIdentified: 2020; **Event:** samplingProtocol: hand net; eventDate: 2017-5-25; year: 2017; month: 5; day: 25; **Record Level:** type: PhysicalObject; institutionCode: ZMB; collectionCode: ZMB; basisOfRecord: PreservedSpecimen
- **Type status:**Other material. **Occurrence:** catalogNumber: ZMB 30288; recordedBy: Do Van Tu; disposition: in collection; **Taxon:** scientificName: *Caridinalanceifrons* Yu, 1936; kingdom: Animal; phylum: Arthropoda; class: Malacostraca; order: Decapoda; family: Atyidae; genus: Caridina; specificEpithet: *lanceifrons*; taxonRank: Species; scientificNameAuthorship: Yu, 1936; vernacularName: atyid shrimp; nomenclaturalCode: ICZN; taxonomicStatus: accepted; **Location:** country: Vietnam; countryCode: VN; stateProvince: Hoa Binh; county: Lac Son; municipality: Nhan Nghia; decimalLatitude: 20.51861; decimalLongitude: 105.44417; geodeticDatum: WGS84; georeferenceProtocol: GPS; **Identification:** identifiedBy: Do Van Tu; dateIdentified: 2020; **Event:** samplingProtocol: hand net; eventDate: 2017-4-23; year: 2017; month: 4; day: 23; **Record Level:** type: PhysicalObject; institutionCode: ZMB; collectionCode: ZMB; basisOfRecord: PreservedSpecimen
- **Type status:**Other material. **Occurrence:** catalogNumber: ZMB 30292; recordedBy: Do Van Tu; disposition: in collection; **Taxon:** scientificName: *Caridinalanceifrons* Yu, 1936; kingdom: Animal; phylum: Arthropoda; class: Malacostraca; order: Decapoda; family: Atyidae; genus: Caridina; specificEpithet: *lanceifrons*; taxonRank: Species; scientificNameAuthorship: Yu, 1936; vernacularName: atyid shrimp; nomenclaturalCode: ICZN; taxonomicStatus: accepted; **Location:** country: Vietnam; countryCode: VN; stateProvince: Yen Bai; county: Van Yen; locality: Nhay, Chau Que Thuong; decimalLatitude: 22.0719; decimalLongitude: 104.46180; geodeticDatum: WGS84; georeferenceProtocol: GPS; **Identification:** identifiedBy: Do Van Tu; dateIdentified: 2020; **Event:** samplingProtocol: hand net; eventDate: 2017-9-18; year: 2017; month: 9; day: 18; **Record Level:** type: PhysicalObject; institutionCode: ZMB; collectionCode: ZMB; basisOfRecord: PreservedSpecimen
- **Type status:**Other material. **Occurrence:** catalogNumber: ZMB 30294; recordedBy: Do Van Tu; disposition: in collection; **Taxon:** scientificName: *Caridinalanceifrons* Yu, 1936; kingdom: Animal; phylum: Arthropoda; class: Malacostraca; order: Decapoda; family: Atyidae; genus: Caridina; specificEpithet: *lanceifrons*; taxonRank: Species; scientificNameAuthorship: Yu, 1936; vernacularName: atyid shrimp; nomenclaturalCode: ICZN; taxonomicStatus: accepted; **Location:** country: Vietnam; countryCode: VN; stateProvince: Lao Cai; decimalLatitude: 22.42587; decimalLongitude: 104.02684; geodeticDatum: WGS84; georeferenceProtocol: GPS; **Identification:** identifiedBy: Do Van Tu; dateIdentified: 2020; **Event:** samplingProtocol: hand net; eventDate: 2017-9-16; year: 2017; month: 9; day: 16; **Record Level:** type: PhysicalObject; institutionCode: ZMB; collectionCode: ZMB; basisOfRecord: PreservedSpecimen
- **Type status:**Other material. **Occurrence:** catalogNumber: ZMB 30299; recordedBy: Do Van Tu; disposition: in collection; **Taxon:** scientificName: *Caridinalanceifrons* Yu, 1936; kingdom: Animal; phylum: Arthropoda; class: Malacostraca; order: Decapoda; family: Atyidae; genus: Caridina; specificEpithet: *lanceifrons*; taxonRank: Species; scientificNameAuthorship: Yu, 1936; vernacularName: atyid shrimp; nomenclaturalCode: ICZN; taxonomicStatus: accepted; **Location:** country: Vietnam; countryCode: VN; stateProvince: Quang Nam; county: Phuc Son; locality: Phuoc Son; decimalLatitude: 15.56702; decimalLongitude: 107.82195; geodeticDatum: WGS84; georeferenceProtocol: GPS; **Identification:** identifiedBy: Do Van Tu; dateIdentified: 2020; **Event:** samplingProtocol: hand net; eventDate: 2017-6-29; year: 2017; month: 6; day: 29; **Record Level:** type: PhysicalObject; institutionCode: ZMB; collectionCode: ZMB; basisOfRecord: PreservedSpecimen
- **Type status:**Other material. **Occurrence:** catalogNumber: ZMB 30307; recordedBy: Do Van Tu; disposition: in collection; **Taxon:** scientificName: *Caridinalanceifrons* Yu, 1936; kingdom: Animal; phylum: Arthropoda; class: Malacostraca; order: Decapoda; family: Atyidae; genus: Caridina; specificEpithet: *lanceifrons*; taxonRank: Species; scientificNameAuthorship: Yu, 1936; vernacularName: atyid shrimp; nomenclaturalCode: ICZN; taxonomicStatus: accepted; **Location:** country: Vietnam; countryCode: VN; stateProvince: Hoa Binh; county: Kim Boi; municipality: Hop Kim; locality: Boi river; decimalLatitude: 20.66067; decimalLongitude: 105.55772; geodeticDatum: WGS84; georeferenceProtocol: GPS; **Identification:** identifiedBy: Do Van Tu; dateIdentified: 2020; **Event:** samplingProtocol: hand net; eventDate: 2017-4-23; year: 2017; month: 4; day: 23; **Record Level:** type: PhysicalObject; institutionCode: ZMB; collectionCode: ZMB; basisOfRecord: PreservedSpecimen
- **Type status:**Other material. **Occurrence:** catalogNumber: ZMB 30318; recordedBy: Do Van Tu; disposition: in collection; **Taxon:** scientificName: *Caridinalanceifrons* Yu, 1936; kingdom: Animal; phylum: Arthropoda; class: Malacostraca; order: Decapoda; family: Atyidae; genus: Caridina; specificEpithet: *lanceifrons*; taxonRank: Species; scientificNameAuthorship: Yu, 1936; vernacularName: atyid shrimp; nomenclaturalCode: ICZN; taxonomicStatus: accepted; **Location:** country: Vietnam; countryCode: VN; stateProvince: Ha Giang; county: Bac Quang; municipality: Tan Thanh; decimalLatitude: 22.54697; decimalLongitude: 104.91033; geodeticDatum: WGS84; georeferenceProtocol: GPS; **Identification:** identifiedBy: Do Van Tu; dateIdentified: 2020; **Event:** samplingProtocol: hand net; eventDate: 2012-11-16; year: 2012; month: 11; day: 16; **Record Level:** type: PhysicalObject; institutionCode: ZMB; collectionCode: ZMB; basisOfRecord: PreservedSpecimen
- **Type status:**Other material. **Occurrence:** catalogNumber: ZMB 30350; recordedBy: Do Van Tu; disposition: in collection; **Taxon:** scientificName: *Caridinalanceifrons* Yu, 1936; kingdom: Animal; phylum: Arthropoda; class: Malacostraca; order: Decapoda; family: Atyidae; genus: Caridina; specificEpithet: *lanceifrons*; taxonRank: Species; scientificNameAuthorship: Yu, 1936; vernacularName: atyid shrimp; nomenclaturalCode: ICZN; taxonomicStatus: accepted; **Location:** country: Vietnam; countryCode: VN; stateProvince: Thai Nguyen; county: Vo Nhai; municipality: Phu Thuong; locality: Mo Ga stream; decimalLatitude: 21.77583; decimalLongitude: 106.12058; geodeticDatum: WGS84; georeferenceProtocol: GPS; **Identification:** identifiedBy: Do Van Tu; dateIdentified: 2020; **Event:** samplingProtocol: hand net; eventDate: 2019-6-9; year: 2019; month: 6; day: 9; **Record Level:** type: PhysicalObject; institutionCode: ZMB; collectionCode: ZMB; basisOfRecord: PreservedSpecimen
- **Type status:**Other material. **Occurrence:** catalogNumber: ZMB 30355; recordedBy: Do Van Tu; disposition: in collection; **Taxon:** scientificName: *Caridinalanceifrons* Yu, 1936; kingdom: Animal; phylum: Arthropoda; class: Malacostraca; order: Decapoda; family: Atyidae; genus: Caridina; specificEpithet: *lanceifrons*; taxonRank: Species; scientificNameAuthorship: Yu, 1936; vernacularName: atyid shrimp; nomenclaturalCode: ICZN; taxonomicStatus: accepted; **Location:** country: Vietnam; countryCode: VN; stateProvince: Son La; county: Quynh Nhai; municipality: Pac Ma; locality: Entrance of a cave; decimalLatitude: 21.7545; decimalLongitude: 103.62372; geodeticDatum: WGS84; georeferenceProtocol: GPS; **Identification:** identifiedBy: Do Van Tu; dateIdentified: 2020; **Event:** samplingProtocol: hand net; eventDate: 2019-7-3; year: 2019; month: 7; day: 3; **Record Level:** type: PhysicalObject; institutionCode: ZMB; collectionCode: ZMB; basisOfRecord: PreservedSpecimen
- **Type status:**Other material. **Occurrence:** catalogNumber: ZMB 30356; recordedBy: Do Van Tu; disposition: in collection; **Taxon:** scientificName: *Caridinalanceifrons* Yu, 1936; kingdom: Animal; phylum: Arthropoda; class: Malacostraca; order: Decapoda; family: Atyidae; genus: Caridina; specificEpithet: *lanceifrons*; taxonRank: Species; scientificNameAuthorship: Yu, 1936; vernacularName: atyid shrimp; nomenclaturalCode: ICZN; taxonomicStatus: accepted; **Location:** country: Vietnam; countryCode: VN; stateProvince: Son La; county: Quynh Nhai; municipality: Pac Ma; decimalLatitude: 21.75449; decimalLongitude: 103.62372; geodeticDatum: WGS84; georeferenceProtocol: GPS; **Identification:** identifiedBy: Do Van Tu; dateIdentified: 2020; **Event:** samplingProtocol: hand net; eventDate: 2019-7-3; year: 2019; month: 7; day: 3; **Record Level:** type: PhysicalObject; institutionCode: ZMB; collectionCode: ZMB; basisOfRecord: PreservedSpecimen
- **Type status:**Other material. **Occurrence:** catalogNumber: ZMB 30357; recordedBy: Do Van Tu; disposition: in collection; **Taxon:** scientificName: *Caridinalanceifrons* Yu, 1936; kingdom: Animal; phylum: Arthropoda; class: Malacostraca; order: Decapoda; family: Atyidae; genus: Caridina; specificEpithet: *lanceifrons*; taxonRank: Species; scientificNameAuthorship: Yu, 1936; vernacularName: atyid shrimp; nomenclaturalCode: ICZN; taxonomicStatus: accepted; **Location:** country: Vietnam; countryCode: VN; stateProvince: Son La; county: Quynh Nhai; municipality: Muong Chien; decimalLatitude: 21.83467; decimalLongitude: 103.59315; geodeticDatum: WGS84; georeferenceProtocol: GPS; **Identification:** identifiedBy: Do Van Tu; dateIdentified: 2020; **Event:** samplingProtocol: hand net; eventDate: 2019-7-3; year: 2019; month: 7; day: 3; **Record Level:** type: PhysicalObject; institutionCode: ZMB; collectionCode: ZMB; basisOfRecord: PreservedSpecimen
- **Type status:**Other material. **Occurrence:** catalogNumber: ZMB 30358; recordedBy: Do Van Tu; disposition: in collection; **Taxon:** scientificName: *Caridinalanceifrons* Yu, 1936; kingdom: Animal; phylum: Arthropoda; class: Malacostraca; order: Decapoda; family: Atyidae; genus: Caridina; specificEpithet: *lanceifrons*; taxonRank: Species; scientificNameAuthorship: Yu, 1936; vernacularName: atyid shrimp; nomenclaturalCode: ICZN; taxonomicStatus: accepted; **Location:** country: Vietnam; countryCode: VN; stateProvince: Yen Bai; county: Van Chan; locality: Cat Thinh; decimalLatitude: 21.47307; decimalLongitude: 104.72254; geodeticDatum: WGS84; georeferenceProtocol: GPS; **Identification:** identifiedBy: Do Van Tu; dateIdentified: 2020; **Event:** samplingProtocol: hand net; eventDate: 2019-7-5; year: 2019; month: 7; day: 5; **Record Level:** type: PhysicalObject; institutionCode: ZMB; collectionCode: ZMB; basisOfRecord: PreservedSpecimen
- **Type status:**Other material. **Occurrence:** catalogNumber: ZMB 30365; recordedBy: Nguyen Tong Cuong; disposition: in collection; **Taxon:** scientificName: *Caridinalanceifrons* Yu, 1936; kingdom: Animal; phylum: Arthropoda; class: Malacostraca; order: Decapoda; family: Atyidae; genus: Caridina; specificEpithet: *lanceifrons*; taxonRank: Species; scientificNameAuthorship: Yu, 1936; vernacularName: atyid shrimp; nomenclaturalCode: ICZN; taxonomicStatus: accepted; **Location:** country: Vietnam; countryCode: VN; stateProvince: Tuyen Quang; county: Ham Yen; municipality: Phu Luu; locality: Kieng stream; decimalLatitude: 22.17092; decimalLongitude: 105.01269; geodeticDatum: WGS84; georeferenceProtocol: GPS; **Identification:** identifiedBy: Do Van Tu; dateIdentified: 2020; **Event:** samplingProtocol: hand net; eventDate: 2018-10-28; year: 2018; month: 10; day: 28; **Record Level:** type: PhysicalObject; institutionCode: ZMB; collectionCode: ZMB; basisOfRecord: PreservedSpecimen
- **Type status:**Other material. **Occurrence:** catalogNumber: ZMB 30366; recordedBy: Nguyen Tong Cuong; disposition: in collection; **Taxon:** scientificName: *Caridinalanceifrons* Yu, 1936; kingdom: Animal; phylum: Arthropoda; class: Malacostraca; order: Decapoda; family: Atyidae; genus: Caridina; specificEpithet: *lanceifrons*; taxonRank: Species; scientificNameAuthorship: Yu, 1936; vernacularName: atyid shrimp; nomenclaturalCode: ICZN; taxonomicStatus: accepted; **Location:** country: Vietnam; countryCode: VN; stateProvince: Tuyen Quang; county: Ham Yen; municipality: Phu Luu; locality: Nam Luong stream 2; decimalLatitude: 22.21023; decimalLongitude: 105.05780; geodeticDatum: WGS84; georeferenceProtocol: GPS; **Identification:** identifiedBy: Do Van Tu; dateIdentified: 2020; **Event:** samplingProtocol: hand net; eventDate: 2018-10-29; year: 2018; month: 10; day: 29; **Record Level:** type: PhysicalObject; institutionCode: ZMB; collectionCode: ZMB; basisOfRecord: PreservedSpecimen
- **Type status:**Other material. **Occurrence:** catalogNumber: ZMB 30370; recordedBy: Nguyen Tong Cuong; disposition: in collection; **Taxon:** scientificName: *Caridinalanceifrons* Yu, 1936; kingdom: Animal; phylum: Arthropoda; class: Malacostraca; order: Decapoda; family: Atyidae; genus: Caridina; specificEpithet: *lanceifrons*; taxonRank: Species; scientificNameAuthorship: Yu, 1936; vernacularName: atyid shrimp; nomenclaturalCode: ICZN; taxonomicStatus: accepted; **Location:** country: Vietnam; countryCode: VN; stateProvince: Tuyen Quang; county: Ham Yen; municipality: Phu Luu; locality: Khang stream; decimalLatitude: 22.16812; decimalLongitude: 104.99143; geodeticDatum: WGS84; georeferenceProtocol: GPS; **Identification:** identifiedBy: Do Van Tu; dateIdentified: 2020; **Event:** samplingProtocol: hand net; eventDate: 2018-10-28; year: 2018; month: 10; day: 28; **Record Level:** type: PhysicalObject; institutionCode: ZMB; collectionCode: ZMB; basisOfRecord: PreservedSpecimen
- **Type status:**Other material. **Occurrence:** catalogNumber: ZMB 30378; recordedBy: Dang Van Dong; disposition: in collection; **Taxon:** scientificName: *Caridinalanceifrons* Yu, 1936; kingdom: Animal; phylum: Arthropoda; class: Malacostraca; order: Decapoda; family: Atyidae; genus: Caridina; specificEpithet: *lanceifrons*; taxonRank: Species; scientificNameAuthorship: Yu, 1936; vernacularName: atyid shrimp; nomenclaturalCode: ICZN; taxonomicStatus: accepted; **Location:** country: Vietnam; countryCode: VN; stateProvince: Ha Giang; county: Vi Xuyen; locality: Stream near Thanh Thuy border (4 km); decimalLatitude: 22.91206; decimalLongitude: 104.873; geodeticDatum: WGS84; georeferenceProtocol: GPS; **Identification:** identifiedBy: Do Van Tu; dateIdentified: 2020; **Event:** samplingProtocol: hand net; eventDate: 2019-2-23; year: 2019; month: 2; day: 23; **Record Level:** type: PhysicalObject; institutionCode: ZMB; collectionCode: ZMB; basisOfRecord: PreservedSpecimen
- **Type status:**Other material. **Occurrence:** catalogNumber: ZMB 30383; recordedBy: Dang Van Dong; disposition: in collection; **Taxon:** scientificName: *Caridinalanceifrons* Yu, 1936; kingdom: Animal; phylum: Arthropoda; class: Malacostraca; order: Decapoda; family: Atyidae; genus: Caridina; specificEpithet: *lanceifrons*; taxonRank: Species; scientificNameAuthorship: Yu, 1936; vernacularName: atyid shrimp; nomenclaturalCode: ICZN; taxonomicStatus: accepted; **Location:** country: Vietnam; countryCode: VN; stateProvince: Lang Son; decimalLatitude: 22.25292; decimalLongitude: 106.4735; geodeticDatum: WGS84; georeferenceProtocol: GPS; **Identification:** identifiedBy: Do Van Tu; dateIdentified: 2020; **Event:** samplingProtocol: hand net; eventDate: 2019-4-27; year: 2019; month: 4; day: 27; **Record Level:** type: PhysicalObject; institutionCode: ZMB; collectionCode: ZMB; basisOfRecord: PreservedSpecimen
- **Type status:**Other material. **Occurrence:** catalogNumber: ZMB 30662; recordedBy: Thomas von Rintelen, Do Van Tu; disposition: in collection; **Taxon:** scientificName: *Caridinalanceifrons* Yu, 1936; kingdom: Animal; phylum: Arthropoda; class: Malacostraca; order: Decapoda; family: Atyidae; genus: Caridina; specificEpithet: *lanceifrons*; taxonRank: Species; scientificNameAuthorship: Yu, 1936; vernacularName: atyid shrimp; nomenclaturalCode: ICZN; taxonomicStatus: accepted; **Location:** country: Vietnam; countryCode: VN; stateProvince: Ninh Binh; locality: River in limestone area Northeast of Cuc Phuong National Park; decimalLatitude: 20.27252; decimalLongitude: 105.91127; geodeticDatum: WGS84; georeferenceProtocol: GPS; **Identification:** identifiedBy: Do Van Tu; dateIdentified: 2020; **Event:** samplingProtocol: hand net; eventDate: 2017-3-4; year: 2017; month: 3; day: 4; **Record Level:** type: PhysicalObject; institutionCode: ZMB; collectionCode: ZMB; basisOfRecord: PreservedSpecimen
- **Type status:**Other material. **Occurrence:** catalogNumber: ZMB 30672; recordedBy: Jens Kühne; disposition: in collection; **Taxon:** scientificName: *Caridinalanceifrons* Yu, 1936; kingdom: Animal; phylum: Arthropoda; class: Malacostraca; order: Decapoda; family: Atyidae; genus: Caridina; specificEpithet: *lanceifrons*; taxonRank: Species; scientificNameAuthorship: Yu, 1936; vernacularName: atyid shrimp; nomenclaturalCode: ICZN; taxonomicStatus: accepted; **Location:** country: Vietnam; countryCode: VN; stateProvince: Da Nang; locality: Small cave pool behind hot spring, off road Da Nang - Prao; decimalLatitude: 15.96194; decimalLongitude: 108.01972; geodeticDatum: WGS84; georeferenceProtocol: GPS; **Identification:** identifiedBy: Do Van Tu; dateIdentified: 2020; **Event:** samplingProtocol: hand net; **Record Level:** type: PhysicalObject; institutionCode: ZMB; collectionCode: ZMB; basisOfRecord: PreservedSpecimen
- **Type status:**Other material. **Occurrence:** catalogNumber: ZMB 30725; recordedBy: Tran Anh Duc; disposition: in collection; **Taxon:** scientificName: *Caridinalanceifrons* Yu, 1936; kingdom: Animal; phylum: Arthropoda; class: Malacostraca; order: Decapoda; family: Atyidae; genus: Caridina; specificEpithet: *lanceifrons*; taxonRank: Species; scientificNameAuthorship: Yu, 1936; vernacularName: atyid shrimp; nomenclaturalCode: ICZN; taxonomicStatus: accepted; **Location:** country: Vietnam; countryCode: VN; stateProvince: Phu Tho; locality: Xuân Sơn National Park, Đồng Sơn, Thân stream, site 2; decimalLatitude: 21.18762; decimalLongitude: 104.87303; geodeticDatum: WGS84; georeferenceProtocol: GPS; **Identification:** identifiedBy: Do Van Tu; dateIdentified: 2020; **Event:** samplingProtocol: hand net; eventDate: 2013-8-29; year: 2013; month: 8; day: 29; **Record Level:** type: PhysicalObject; institutionCode: ZMB; collectionCode: ZMB; basisOfRecord: PreservedSpecimen
- **Type status:**Other material. **Occurrence:** catalogNumber: ZMB 30726; recordedBy: Ngo Xuan Quang; disposition: in collection; **Taxon:** scientificName: *Caridinalanceifrons* Yu, 1936; kingdom: Animal; phylum: Arthropoda; class: Malacostraca; order: Decapoda; family: Atyidae; genus: Caridina; specificEpithet: *lanceifrons*; taxonRank: Species; scientificNameAuthorship: Yu, 1936; vernacularName: atyid shrimp; nomenclaturalCode: ICZN; taxonomicStatus: accepted; **Location:** country: Vietnam; countryCode: VN; stateProvince: Nghe An; county: Con Cuong; locality: Tùng Hương village, Khe Thơi stream; decimalLatitude: 19.08928; decimalLongitude: 104.66718; geodeticDatum: WGS84; georeferenceProtocol: GPS; **Identification:** identifiedBy: Do Van Tu; dateIdentified: 2020; **Event:** samplingProtocol: hand net; eventDate: 2012-12-21; year: 2012; month: 12; day: 21; **Record Level:** type: PhysicalObject; institutionCode: ZMB; collectionCode: ZMB; basisOfRecord: PreservedSpecimen
- **Type status:**Other material. **Occurrence:** catalogNumber: ZMB 30727; recordedBy: Ngo Xuan Quang; disposition: in collection; **Taxon:** scientificName: *Caridinalanceifrons* Yu, 1936; kingdom: Animal; phylum: Arthropoda; class: Malacostraca; order: Decapoda; family: Atyidae; genus: Caridina; specificEpithet: *lanceifrons*; taxonRank: Species; scientificNameAuthorship: Yu, 1936; vernacularName: atyid shrimp; nomenclaturalCode: ICZN; taxonomicStatus: accepted; **Location:** country: Vietnam; countryCode: VN; stateProvince: Nghe An; locality: Pů Mát National Park, Khe Kčm stream, site 4; decimalLatitude: 18.9389; decimalLongitude: 104.80595; geodeticDatum: WGS84; georeferenceProtocol: GPS; **Identification:** identifiedBy: Do Van Tu; dateIdentified: 2020; **Event:** samplingProtocol: hand net; eventDate: 2012-12-20; year: 2012; month: 12; day: 20; **Record Level:** type: PhysicalObject; institutionCode: ZMB; collectionCode: ZMB; basisOfRecord: PreservedSpecimen
- **Type status:**Other material. **Occurrence:** catalogNumber: ZMB 31576; recordedBy: Do Van Tu; disposition: in collection; **Taxon:** scientificName: *Caridinalanceifrons* Yu, 1936; kingdom: Animal; phylum: Arthropoda; class: Malacostraca; order: Decapoda; family: Atyidae; genus: Caridina; specificEpithet: *lanceifrons*; taxonRank: Species; scientificNameAuthorship: Yu, 1936; vernacularName: atyid shrimp; nomenclaturalCode: ICZN; taxonomicStatus: accepted; **Location:** country: Vietnam; countryCode: VN; stateProvince: Quang Ninh; county: Mong Cai; decimalLatitude: 21.54096; decimalLongitude: 107.73986; geodeticDatum: WGS84; georeferenceProtocol: GPS; **Identification:** identifiedBy: Do Van Tu; dateIdentified: 2020; **Event:** samplingProtocol: hand net; eventDate: 2018-1-24; year: 2018; month: 1; day: 24; **Record Level:** type: PhysicalObject; institutionCode: ZMB; collectionCode: ZMB; basisOfRecord: PreservedSpecimen
- **Type status:**Other material. **Occurrence:** catalogNumber: ZMB 31587; recordedBy: Do Van Tu; disposition: in collection; **Taxon:** scientificName: *Caridinalanceifrons* Yu, 1936; kingdom: Animal; phylum: Arthropoda; class: Malacostraca; order: Decapoda; family: Atyidae; genus: Caridina; specificEpithet: *lanceifrons*; taxonRank: Species; scientificNameAuthorship: Yu, 1936; vernacularName: atyid shrimp; nomenclaturalCode: ICZN; taxonomicStatus: accepted; **Location:** country: Vietnam; countryCode: VN; stateProvince: Lai Chau; decimalLatitude: 22.27960; decimalLongitude: 103.38803; geodeticDatum: WGS84; georeferenceProtocol: GPS; **Identification:** identifiedBy: Do Van Tu; dateIdentified: 2020; **Event:** samplingProtocol: hand net; eventDate: 2018-4-29; year: 2018; month: 4; day: 29; **Record Level:** type: PhysicalObject; institutionCode: ZMB; collectionCode: ZMB; basisOfRecord: PreservedSpecimen
- **Type status:**Other material. **Occurrence:** catalogNumber: ZMB 31589; recordedBy: Do Van Tu; disposition: in collection; **Taxon:** scientificName: *Caridinalanceifrons* Yu, 1936; kingdom: Animal; phylum: Arthropoda; class: Malacostraca; order: Decapoda; family: Atyidae; genus: Caridina; specificEpithet: *lanceifrons*; taxonRank: Species; scientificNameAuthorship: Yu, 1936; vernacularName: atyid shrimp; nomenclaturalCode: ICZN; taxonomicStatus: accepted; **Location:** country: Vietnam; countryCode: VN; stateProvince: Lai Chau; decimalLatitude: 22.32786; decimalLongitude: 103.35493; geodeticDatum: WGS84; georeferenceProtocol: GPS; **Identification:** identifiedBy: Do Van Tu; dateIdentified: 2020; **Event:** samplingProtocol: hand net; eventDate: 2018-4-30; year: 2018; month: 4; day: 30; **Record Level:** type: PhysicalObject; institutionCode: ZMB; collectionCode: ZMB; basisOfRecord: PreservedSpecimen
- **Type status:**Other material. **Occurrence:** catalogNumber: ZMB 31594; recordedBy: Do Van Tu; disposition: in collection; **Taxon:** scientificName: *Caridinalanceifrons* Yu, 1936; kingdom: Animal; phylum: Arthropoda; class: Malacostraca; order: Decapoda; family: Atyidae; genus: Caridina; specificEpithet: *lanceifrons*; taxonRank: Species; scientificNameAuthorship: Yu, 1936; vernacularName: atyid shrimp; nomenclaturalCode: ICZN; taxonomicStatus: accepted; **Location:** country: Vietnam; countryCode: VN; stateProvince: Lang Son; decimalLatitude: 21.47528; decimalLongitude: 107.16556; geodeticDatum: WGS84; georeferenceProtocol: GPS; **Identification:** identifiedBy: Do Van Tu; dateIdentified: 2020; **Event:** samplingProtocol: hand net; eventDate: 2018-3-18; year: 2018; month: 3; day: 18; **Record Level:** type: PhysicalObject; institutionCode: ZMB; collectionCode: ZMB; basisOfRecord: PreservedSpecimen
- **Type status:**Other material. **Occurrence:** catalogNumber: ZMB 31597; recordedBy: Do Van Tu; disposition: in collection; **Taxon:** scientificName: *Caridinalanceifrons* Yu, 1936; kingdom: Animal; phylum: Arthropoda; class: Malacostraca; order: Decapoda; family: Atyidae; genus: Caridina; specificEpithet: *lanceifrons*; taxonRank: Species; scientificNameAuthorship: Yu, 1936; vernacularName: atyid shrimp; nomenclaturalCode: ICZN; taxonomicStatus: accepted; **Location:** country: Vietnam; countryCode: VN; stateProvince: Quang Ninh; county: Tien Yen; locality: Khe Cau; decimalLatitude: 21.38944; decimalLongitude: 107.27306; geodeticDatum: WGS84; georeferenceProtocol: GPS; **Identification:** identifiedBy: Do Van Tu; dateIdentified: 2020; **Event:** samplingProtocol: hand net; eventDate: 2018-3-19; year: 2018; month: 3; day: 19; **Record Level:** type: PhysicalObject; institutionCode: ZMB; collectionCode: ZMB; basisOfRecord: PreservedSpecimen
- **Type status:**Other material. **Occurrence:** catalogNumber: ZMB 31598; recordedBy: Do Van Tu; disposition: in collection; **Taxon:** scientificName: *Caridinalanceifrons* Yu, 1936; kingdom: Animal; phylum: Arthropoda; class: Malacostraca; order: Decapoda; family: Atyidae; genus: Caridina; specificEpithet: *lanceifrons*; taxonRank: Species; scientificNameAuthorship: Yu, 1936; vernacularName: atyid shrimp; nomenclaturalCode: ICZN; taxonomicStatus: accepted; **Location:** country: Vietnam; countryCode: VN; stateProvince: Bac Kan; county: Cho Moi; decimalLatitude: 21.89644; decimalLongitude: 105.79489; geodeticDatum: WGS84; georeferenceProtocol: GPS; **Identification:** identifiedBy: Do Van Tu; dateIdentified: 2020; **Event:** samplingProtocol: hand net; eventDate: 2018-3-16; year: 2018; month: 3; day: 16; **Record Level:** type: PhysicalObject; institutionCode: ZMB; collectionCode: ZMB; basisOfRecord: PreservedSpecimen
- **Type status:**Other material. **Occurrence:** catalogNumber: ZMB 31609; recordedBy: Do Van Tu; disposition: in collection; **Taxon:** scientificName: *Caridinalanceifrons* Yu, 1936; kingdom: Animal; phylum: Arthropoda; class: Malacostraca; order: Decapoda; family: Atyidae; genus: Caridina; specificEpithet: *lanceifrons*; taxonRank: Species; scientificNameAuthorship: Yu, 1936; vernacularName: atyid shrimp; nomenclaturalCode: ICZN; taxonomicStatus: accepted; **Location:** country: Vietnam; countryCode: VN; stateProvince: Thua Thien-Hue; locality: Bach Ma National Park, Khe Phen Tren; decimalLatitude: 16.21725; decimalLongitude: 107.87695; geodeticDatum: WGS84; georeferenceProtocol: GPS; **Identification:** identifiedBy: Do Van Tu; dateIdentified: 2020; **Event:** samplingProtocol: hand net; eventDate: 2018-6-6; year: 2018; month: 6; day: 6; **Record Level:** type: PhysicalObject; institutionCode: ZMB; collectionCode: ZMB; basisOfRecord: PreservedSpecimen
- **Type status:**Other material. **Occurrence:** catalogNumber: ZMB 31611; recordedBy: Virginia Duwe; disposition: in collection; **Taxon:** scientificName: *Caridinalanceifrons* Yu, 1936; kingdom: Animal; phylum: Arthropoda; class: Malacostraca; order: Decapoda; family: Atyidae; genus: Caridina; specificEpithet: *lanceifrons*; taxonRank: Species; scientificNameAuthorship: Yu, 1936; vernacularName: atyid shrimp; nomenclaturalCode: ICZN; taxonomicStatus: accepted; **Location:** country: Vietnam; countryCode: VN; stateProvince: Thua Thien-Hue; locality: Bach Ma National Park, Khe Su; decimalLatitude: 16.22428; decimalLongitude: 107.882; geodeticDatum: WGS84; georeferenceProtocol: GPS; **Identification:** identifiedBy: Do Van Tu; dateIdentified: 2020; **Event:** samplingProtocol: hand net; eventDate: 2018-6-11; year: 2018; month: 6; day: 11; **Record Level:** type: PhysicalObject; institutionCode: ZMB; collectionCode: ZMB; basisOfRecord: PreservedSpecimen
- **Type status:**Other material. **Occurrence:** catalogNumber: ZMB 31624; recordedBy: Andreas Karge; disposition: in collection; **Taxon:** scientificName: *Caridinalanceifrons* Yu, 1936; kingdom: Animal; phylum: Arthropoda; class: Malacostraca; order: Decapoda; family: Atyidae; genus: Caridina; specificEpithet: *lanceifrons*; taxonRank: Species; scientificNameAuthorship: Yu, 1936; vernacularName: atyid shrimp; nomenclaturalCode: ICZN; taxonomicStatus: accepted; **Location:** country: Vietnam; countryCode: VN; stateProvince: Da Nang; locality: Hai Van Pass (Southeast); decimalLatitude: 16.16935; decimalLongitude: 108.12638; geodeticDatum: WGS84; georeferenceProtocol: GPS; **Identification:** identifiedBy: Do Van Tu; dateIdentified: 2020; **Event:** samplingProtocol: hand net; eventDate: 2009-7-16; year: 2009; month: 7; day: 16; **Record Level:** type: PhysicalObject; institutionCode: ZMB; collectionCode: ZMB; basisOfRecord: PreservedSpecimen
- **Type status:**Other material. **Occurrence:** catalogNumber: ZMB 32285; recordedBy: Do Van Tu; disposition: in collection; **Taxon:** scientificName: *Caridinalanceifrons* Yu, 1936; kingdom: Animal; phylum: Arthropoda; class: Malacostraca; order: Decapoda; family: Atyidae; genus: Caridina; specificEpithet: *lanceifrons*; taxonRank: Species; scientificNameAuthorship: Yu, 1936; vernacularName: atyid shrimp; nomenclaturalCode: ICZN; taxonomicStatus: accepted; **Location:** country: Vietnam; countryCode: VN; stateProvince: Ha Nam; county: Kim Bang; decimalLatitude: 20.57350; decimalLongitude: 105.83371; geodeticDatum: WGS84; georeferenceProtocol: GPS; **Identification:** identifiedBy: Do Van Tu; dateIdentified: 2020; **Event:** samplingProtocol: hand net; eventDate: 2020-5-31; year: 2020; month: 5; day: 31; **Record Level:** type: PhysicalObject; institutionCode: ZMB; collectionCode: ZMB; basisOfRecord: PreservedSpecimen
- **Type status:**Other material. **Occurrence:** catalogNumber: ZMB 33800; recordedBy: Do Van Tu; disposition: in collection; **Taxon:** scientificName: *Caridinalanceifrons* Yu, 1936; kingdom: Animal; phylum: Arthropoda; class: Malacostraca; order: Decapoda; family: Atyidae; genus: Caridina; specificEpithet: *lanceifrons*; taxonRank: Species; scientificNameAuthorship: Yu, 1936; vernacularName: atyid shrimp; nomenclaturalCode: ICZN; taxonomicStatus: accepted; **Location:** country: Vietnam; countryCode: VN; stateProvince: Nam Dinh; county: Nghia Hung; decimalLatitude: 20.19045; decimalLongitude: 106.18031; geodeticDatum: WGS84; georeferenceProtocol: GPS; **Identification:** identifiedBy: Do Van Tu; dateIdentified: 2020; **Event:** samplingProtocol: hand net; eventDate: 2020-5-31; year: 2020; month: 5; day: 31; **Record Level:** type: PhysicalObject; institutionCode: ZMB; collectionCode: ZMB; basisOfRecord: PreservedSpecimen
- **Type status:**Other material. **Occurrence:** catalogNumber: ZMB 33811; recordedBy: Do Van Tu; disposition: in collection; **Taxon:** scientificName: *Caridinalanceifrons* Yu, 1936; kingdom: Animal; phylum: Arthropoda; class: Malacostraca; order: Decapoda; family: Atyidae; genus: Caridina; specificEpithet: *lanceifrons*; taxonRank: Species; scientificNameAuthorship: Yu, 1936; vernacularName: atyid shrimp; nomenclaturalCode: ICZN; taxonomicStatus: accepted; **Location:** country: Vietnam; countryCode: VN; stateProvince: Ha Giang; county: Bac Quang; verbatimElevation: 342 m; decimalLatitude: 22.51194; decimalLongitude: 104.84938; geodeticDatum: WGS84; georeferenceProtocol: GPS; **Identification:** identifiedBy: Do Van Tu; dateIdentified: 2020; **Event:** samplingProtocol: hand net; eventDate: 2020-10-12; year: 2020; month: 10; day: 12; **Record Level:** type: PhysicalObject; institutionCode: ZMB; collectionCode: ZMB; basisOfRecord: PreservedSpecimen
- **Type status:**Other material. **Occurrence:** catalogNumber: ZMB 33812; recordedBy: Do Van Tu; disposition: in collection; **Taxon:** scientificName: *Caridinalanceifrons* Yu, 1936; kingdom: Animal; phylum: Arthropoda; class: Malacostraca; order: Decapoda; family: Atyidae; genus: Caridina; specificEpithet: *lanceifrons*; taxonRank: Species; scientificNameAuthorship: Yu, 1936; vernacularName: atyid shrimp; nomenclaturalCode: ICZN; taxonomicStatus: accepted; **Location:** country: Vietnam; countryCode: VN; stateProvince: Ha Giang; county: Bac Quang; verbatimElevation: 262 m a.s.l; decimalLatitude: 22.50507; decimalLongitude: 104.85352; geodeticDatum: WGS84; georeferenceProtocol: GPS; **Identification:** identifiedBy: Do Van Tu; dateIdentified: 2020; **Event:** samplingProtocol: hand net; eventDate: 2020-10-12; year: 2020; month: 10; day: 12; **Record Level:** type: PhysicalObject; institutionCode: ZMB; collectionCode: ZMB; basisOfRecord: PreservedSpecimen
- **Type status:**Other material. **Occurrence:** catalogNumber: ZMB 33795; recordedBy: Do Van Tu, Dang Van Dong, Nguyen Tong Cuong'; disposition: in collection; **Taxon:** scientificName: *Caridinalanceifrons* Yu, 1936; kingdom: Animal; phylum: Arthropoda; class: Malacostraca; order: Decapoda; family: Atyidae; genus: Caridina; specificEpithet: *lanceifrons*; taxonRank: Species; scientificNameAuthorship: Yu, 1936; vernacularName: atyid shrimp; nomenclaturalCode: ICZN; taxonomicStatus: accepted; **Location:** country: Vietnam; countryCode: VN; stateProvince: Bac Kan; county: Cho Don; verbatimElevation: 277 m a.s.l.; decimalLatitude: 22.21619; decimalLongitude: 105.49067; geodeticDatum: WGS84; georeferenceProtocol: GPS; **Identification:** identifiedBy: Do Van Tu; dateIdentified: 2020; **Event:** samplingProtocol: hand net; eventDate: 2020-7-26; year: 2020; month: 7; day: 26; **Record Level:** type: PhysicalObject; institutionCode: ZMB; collectionCode: ZMB; basisOfRecord: PreservedSpecimen
- **Type status:**Other material. **Occurrence:** catalogNumber: ZMB 33794; recordedBy: Do Van Tu, Dang Van Dong, Nguyen Tong Cuong'; disposition: in collection; **Taxon:** scientificName: *Caridinalanceifrons* Yu, 1936; kingdom: Animal; phylum: Arthropoda; class: Malacostraca; order: Decapoda; family: Atyidae; genus: Caridina; specificEpithet: *lanceifrons*; taxonRank: Species; scientificNameAuthorship: Yu, 1936; vernacularName: atyid shrimp; nomenclaturalCode: ICZN; taxonomicStatus: accepted; **Location:** country: Vietnam; countryCode: VN; stateProvince: Bac Kan; county: Cho Don; verbatimElevation: 300 m a.s.l.; decimalLatitude: 22.29353; decimalLongitude: 105.55729; geodeticDatum: WGS84; georeferenceProtocol: GPS; **Identification:** identifiedBy: Do Van Tu; dateIdentified: 2020; **Event:** samplingProtocol: hand net; eventDate: 2020-7-29; year: 2020; month: 7; day: 29; **Record Level:** type: PhysicalObject; institutionCode: ZMB; collectionCode: ZMB; basisOfRecord: PreservedSpecimen
- **Type status:**Other material. **Occurrence:** catalogNumber: ZMB 33796; recordedBy: Nguyen Dinh Tao; disposition: in collection; **Taxon:** scientificName: *Caridinalanceifrons* Yu, 1936; kingdom: Animal; phylum: Arthropoda; class: Malacostraca; order: Decapoda; family: Atyidae; genus: Caridina; specificEpithet: *lanceifrons*; taxonRank: Species; scientificNameAuthorship: Yu, 1936; vernacularName: atyid shrimp; nomenclaturalCode: ICZN; taxonomicStatus: accepted; **Location:** country: Vietnam; countryCode: VN; stateProvince: Dien Bien; locality: Muong Cha; decimalLatitude: 21.90375; decimalLongitude: 103.27797; geodeticDatum: WGS84; georeferenceProtocol: GPS; **Identification:** identifiedBy: Do Van Tu; dateIdentified: 2020; **Event:** samplingProtocol: hand net; eventDate: 2021-1-13; year: 2021; month: 1; day: 13; **Record Level:** type: PhysicalObject; institutionCode: ZMB; collectionCode: ZMB; basisOfRecord: PreservedSpecimen
- **Type status:**Other material. **Occurrence:** catalogNumber: HUS; recordedBy: Do Van Tu; disposition: in collection; **Taxon:** scientificName: *Caridinalanceifrons* Yu, 1936; kingdom: Animal; phylum: Arthropoda; class: Malacostraca; order: Decapoda; family: Atyidae; genus: Caridina; specificEpithet: *lanceifrons*; taxonRank: Species; scientificNameAuthorship: Yu, 1936; vernacularName: atyid shrimp; nomenclaturalCode: ICZN; taxonomicStatus: accepted; **Location:** country: Vietnam; countryCode: VN; stateProvince: Ha Noi; county: Phu Xuyen; decimalLatitude: 20.78683; decimalLongitude: 105.92394; geodeticDatum: WGS84; georeferenceProtocol: GPS; **Identification:** identifiedBy: Do Van Tu; dateIdentified: 2020; **Event:** samplingProtocol: hand net; eventDate: 2020-7; year: 2020; month: 7; **Record Level:** type: PhysicalObject; institutionCode: HUS; collectionCode: HUS; basisOfRecord: PreservedSpecimen
- **Type status:**Other material. **Occurrence:** catalogNumber: ZMB 33814; recordedBy: Do Van Tu; disposition: in collection; **Taxon:** scientificName: *Caridinalanceifrons* Yu, 1936; kingdom: Animal; phylum: Arthropoda; class: Malacostraca; order: Decapoda; family: Atyidae; genus: Caridina; specificEpithet: *lanceifrons*; taxonRank: Species; scientificNameAuthorship: Yu, 1936; vernacularName: atyid shrimp; nomenclaturalCode: ICZN; taxonomicStatus: accepted; **Location:** country: Vietnam; countryCode: VN; stateProvince: Hung Yen; county: Khoai Chau; decimalLatitude: 20.84383; decimalLongitude: 105.92075; geodeticDatum: WGS84; georeferenceProtocol: GPS; **Identification:** identifiedBy: Do Van Tu; dateIdentified: 2020; **Event:** samplingProtocol: hand net; eventDate: 2020-7; year: 2020; month: 7; **Record Level:** type: PhysicalObject; institutionCode: ZMB; collectionCode: ZMB; basisOfRecord: PreservedSpecimen
- **Type status:**Other material. **Occurrence:** catalogNumber: ZMB 33816; recordedBy: Do Van Tu; disposition: in collection; **Taxon:** scientificName: *Caridinalanceifrons* Yu, 1936; kingdom: Animal; phylum: Arthropoda; class: Malacostraca; order: Decapoda; family: Atyidae; genus: Caridina; specificEpithet: *lanceifrons*; taxonRank: Species; scientificNameAuthorship: Yu, 1936; vernacularName: atyid shrimp; nomenclaturalCode: ICZN; taxonomicStatus: accepted; **Location:** country: Vietnam; countryCode: VN; stateProvince: Ha Noi; county: Son Tay; decimalLatitude: 21.15839; decimalLongitude: 105.48942; geodeticDatum: WGS84; georeferenceProtocol: GPS; **Identification:** identifiedBy: Do Van Tu; dateIdentified: 2020; **Event:** samplingProtocol: hand net; eventDate: 2020-7; year: 2020; month: 7; **Record Level:** type: PhysicalObject; institutionCode: ZMB; collectionCode: ZMB; basisOfRecord: PreservedSpecimen
- **Type status:**Other material. **Occurrence:** catalogNumber: HUS; recordedBy: Do Van Tu; disposition: in collection; **Taxon:** scientificName: *Caridinalanceifrons* Yu, 1936; kingdom: Animal; phylum: Arthropoda; class: Malacostraca; order: Decapoda; family: Atyidae; genus: Caridina; specificEpithet: *lanceifrons*; taxonRank: Species; scientificNameAuthorship: Yu, 1936; vernacularName: atyid shrimp; nomenclaturalCode: ICZN; taxonomicStatus: accepted; **Location:** country: Vietnam; countryCode: VN; stateProvince: Yen Bai; county: Van Yen; decimalLatitude: 21.88031; decimalLongitude: 104.67986; geodeticDatum: WGS84; georeferenceProtocol: GPS; **Identification:** identifiedBy: Do Van Tu; dateIdentified: 2020; **Event:** samplingProtocol: hand net; eventDate: 2020-7; year: 2020; month: 7; **Record Level:** type: PhysicalObject; institutionCode: HUS; collectionCode: HUS; basisOfRecord: PreservedSpecimen
- **Type status:**Other material. **Occurrence:** catalogNumber: ZMB 33818; recordedBy: Do Van Tu; disposition: in collection; **Taxon:** scientificName: *Caridinalanceifrons* Yu, 1936; kingdom: Animal; phylum: Arthropoda; class: Malacostraca; order: Decapoda; family: Atyidae; genus: Caridina; specificEpithet: *lanceifrons*; taxonRank: Species; scientificNameAuthorship: Yu, 1936; vernacularName: atyid shrimp; nomenclaturalCode: ICZN; taxonomicStatus: accepted; **Location:** country: Vietnam; countryCode: VN; stateProvince: Lao Cai; county: Bao Thang; decimalLatitude: 22.45364; decimalLongitude: 104.02578; geodeticDatum: WGS84; georeferenceProtocol: GPS; **Identification:** identifiedBy: Do Van Tu; dateIdentified: 2020; **Event:** samplingProtocol: hand net; eventDate: 2020-7-16; year: 2020; month: 7; day: 16; **Record Level:** type: PhysicalObject; institutionCode: ZMB; collectionCode: ZMB; basisOfRecord: PreservedSpecimen
- **Type status:**Other material. **Occurrence:** catalogNumber: ZMB 33819; recordedBy: Do Van Tu; disposition: in collection; **Taxon:** scientificName: *Caridinalanceifrons* Yu, 1936; kingdom: Animal; phylum: Arthropoda; class: Malacostraca; order: Decapoda; family: Atyidae; genus: Caridina; specificEpithet: *lanceifrons*; taxonRank: Species; scientificNameAuthorship: Yu, 1936; vernacularName: atyid shrimp; nomenclaturalCode: ICZN; taxonomicStatus: accepted; **Location:** country: Vietnam; countryCode: VN; stateProvince: Lao Cai; decimalLatitude: 22.48606; decimalLongitude: 103.97786; geodeticDatum: WGS84; georeferenceProtocol: GPS; **Identification:** identifiedBy: Do Van Tu; dateIdentified: 2020; **Event:** samplingProtocol: hand net; eventDate: 2020-7; year: 2020; month: 7; **Record Level:** type: PhysicalObject; institutionCode: ZMB; collectionCode: ZMB; basisOfRecord: PreservedSpecimen
- **Type status:**Other material. **Occurrence:** catalogNumber: ZMB 33821; recordedBy: Do Van Tu; disposition: in collection; **Taxon:** scientificName: *Caridinalanceifrons* Yu, 1936; kingdom: Animal; phylum: Arthropoda; class: Malacostraca; order: Decapoda; family: Atyidae; genus: Caridina; specificEpithet: *lanceifrons*; taxonRank: Species; scientificNameAuthorship: Yu, 1936; vernacularName: atyid shrimp; nomenclaturalCode: ICZN; taxonomicStatus: accepted; **Location:** country: Vietnam; countryCode: VN; stateProvince: Yen Bai; county: Van Yen; decimalLatitude: 21.87769; decimalLongitude: 104.67942; geodeticDatum: WGS84; georeferenceProtocol: GPS; **Identification:** identifiedBy: Do Van Tu; dateIdentified: 2020; **Event:** samplingProtocol: hand net; eventDate: 2020-7-17; year: 2020; month: 7; day: 17; **Record Level:** type: PhysicalObject; institutionCode: ZMB; collectionCode: ZMB; basisOfRecord: PreservedSpecimen
- **Type status:**Other material. **Occurrence:** catalogNumber: HUS; recordedBy: Do Van Tu; disposition: in collection; **Taxon:** scientificName: *Caridinalanceifrons* Yu, 1936; kingdom: Animal; phylum: Arthropoda; class: Malacostraca; order: Decapoda; family: Atyidae; genus: Caridina; specificEpithet: *lanceifrons*; taxonRank: Species; scientificNameAuthorship: Yu, 1936; vernacularName: atyid shrimp; nomenclaturalCode: ICZN; taxonomicStatus: accepted; **Location:** country: Vietnam; countryCode: VN; stateProvince: Lao Cai; decimalLatitude: 22.48533; decimalLongitude: 103.97714; geodeticDatum: WGS84; georeferenceProtocol: GPS; **Identification:** identifiedBy: Do Van Tu; dateIdentified: 2020; **Event:** samplingProtocol: hand net; eventDate: 2020-7-15; year: 2020; month: 7; day: 15; **Record Level:** type: PhysicalObject; institutionCode: HUS; collectionCode: HUS; basisOfRecord: PreservedSpecimen
- **Type status:**Other material. **Occurrence:** catalogNumber: ZMB 33823; recordedBy: Do Van Tu; disposition: in collection; **Taxon:** scientificName: *Caridinalanceifrons* Yu, 1936; kingdom: Animal; phylum: Arthropoda; class: Malacostraca; order: Decapoda; family: Atyidae; genus: Caridina; specificEpithet: *lanceifrons*; taxonRank: Species; scientificNameAuthorship: Yu, 1936; vernacularName: atyid shrimp; nomenclaturalCode: ICZN; taxonomicStatus: accepted; **Location:** country: Vietnam; countryCode: VN; stateProvince: Vinh Phuc; county: Vinh Tuong; decimalLatitude: 21.25083; decimalLongitude: 105.44683; geodeticDatum: WGS84; georeferenceProtocol: GPS; **Identification:** identifiedBy: Do Van Tu; dateIdentified: 2020; **Event:** samplingProtocol: hand net; eventDate: 2020-7-19; year: 2020; month: 7; day: 19; **Record Level:** type: PhysicalObject; institutionCode: ZMB; collectionCode: ZMB; basisOfRecord: PreservedSpecimen
- **Type status:**Other material. **Occurrence:** catalogNumber: HUS; recordedBy: Do Van Tu; disposition: in collection; **Taxon:** scientificName: *Caridinalanceifrons* Yu, 1936; kingdom: Animal; phylum: Arthropoda; class: Malacostraca; order: Decapoda; family: Atyidae; genus: Caridina; specificEpithet: *lanceifrons*; taxonRank: Species; scientificNameAuthorship: Yu, 1936; vernacularName: atyid shrimp; nomenclaturalCode: ICZN; taxonomicStatus: accepted; **Location:** country: Vietnam; countryCode: VN; stateProvince: Yen Bai; county: Van Yen; decimalLatitude: 22.00508; decimalLongitude: 104.54206; geodeticDatum: WGS84; georeferenceProtocol: GPS; **Identification:** identifiedBy: Do Van Tu; dateIdentified: 2020; **Event:** samplingProtocol: hand net; eventDate: 2020-7-16; year: 2020; month: 7; day: 16; **Record Level:** type: PhysicalObject; institutionCode: HUS; collectionCode: HUS; basisOfRecord: PreservedSpecimen
- **Type status:**Other material. **Occurrence:** catalogNumber: ZMB 33825; recordedBy: Do Van Tu; disposition: in collection; **Taxon:** scientificName: *Caridinalanceifrons* Yu, 1936; kingdom: Animal; phylum: Arthropoda; class: Malacostraca; order: Decapoda; family: Atyidae; genus: Caridina; specificEpithet: *lanceifrons*; taxonRank: Species; scientificNameAuthorship: Yu, 1936; vernacularName: atyid shrimp; nomenclaturalCode: ICZN; taxonomicStatus: accepted; **Location:** country: Vietnam; countryCode: VN; stateProvince: Ha Noi; county: Dan Phuong; decimalLatitude: 21.10503; decimalLongitude: 105.73303; geodeticDatum: WGS84; georeferenceProtocol: GPS; **Identification:** identifiedBy: Do Van Tu; dateIdentified: 2020; **Event:** samplingProtocol: hand net; eventDate: 2020-7; year: 2020; month: 7; **Record Level:** type: PhysicalObject; institutionCode: ZMB; collectionCode: ZMB; basisOfRecord: PreservedSpecimen
- **Type status:**Other material. **Occurrence:** catalogNumber: ZMB 33826; recordedBy: Do Van Tu; disposition: in collection; **Taxon:** scientificName: *Caridinalanceifrons* Yu, 1936; kingdom: Animal; phylum: Arthropoda; class: Malacostraca; order: Decapoda; family: Atyidae; genus: Caridina; specificEpithet: *lanceifrons*; taxonRank: Species; scientificNameAuthorship: Yu, 1936; vernacularName: atyid shrimp; nomenclaturalCode: ICZN; taxonomicStatus: accepted; **Location:** country: Vietnam; countryCode: VN; stateProvince: Yen Bai; county: Van Yen; locality: Au Lau; decimalLatitude: 21.70664; decimalLongitude: 104.86636; geodeticDatum: WGS84; georeferenceProtocol: GPS; **Identification:** identifiedBy: Do Van Tu; dateIdentified: 2020; **Event:** samplingProtocol: hand net; eventDate: 2020-7; year: 2020; month: 7; **Record Level:** type: PhysicalObject; institutionCode: ZMB; collectionCode: ZMB; basisOfRecord: PreservedSpecimen
- **Type status:**Other material. **Occurrence:** catalogNumber: ZMB 32691; recordedBy: Marco Endruweit, Wang Jing; disposition: in collection; **Taxon:** scientificName: *Caridinalanceifrons* Yu, 1936; kingdom: Animal; phylum: Arthropoda; class: Malacostraca; order: Decapoda; family: Atyidae; genus: Caridina; specificEpithet: *lanceifrons*; taxonRank: Species; scientificNameAuthorship: Yu, 1936; vernacularName: atyid shrimp; nomenclaturalCode: ICZN; taxonomicStatus: accepted; **Location:** country: China; countryCode: CN; stateProvince: Hainan; locality: Shilinghe River, 1 km North of Bacun (upstream), off the major road 40 Baoting - Lingshui, Wuzhishan Mountain South face, confluence of two streams, streams left and right of road, Lingshuihe River basin, Baoting County; decimalLatitude: 18.75328; decimalLongitude: 109.73838; geodeticDatum: WGS84; georeferenceProtocol: GPS; **Identification:** identifiedBy: Do Van Tu; dateIdentified: 2020; **Event:** samplingProtocol: hand net; eventDate: 2011-7-27; year: 2011; month: 7; day: 27; **Record Level:** type: PhysicalObject; institutionCode: ZMB; collectionCode: ZMB; basisOfRecord: PreservedSpecimen
- **Type status:**Other material. **Occurrence:** catalogNumber: ZMB 29689; recordedBy: Marco Endruweit, Wang Jing; disposition: in collection; **Taxon:** scientificName: *Caridinalanceifrons* Yu, 1936; kingdom: Animal; phylum: Arthropoda; class: Malacostraca; order: Decapoda; family: Atyidae; genus: Caridina; specificEpithet: lanceifrons; taxonRank: Species; scientificNameAuthorship: Yu, 1936; vernacularName: atyid shrimp; nomenclaturalCode: ICZN; taxonomicStatus: accepted; **Location:** country: China; countryCode: CN; stateProvince: Hainan; locality: Hillstream at road Baisha - Nankai, crossing road from right to left, between Nanmeicun and Nanlancun, tributary to Nankaihe River, a southern tributary of the large Songtao Reservoir, Nandujiang River basin; decimalLatitude: 19.10608; decimalLongitude: 109.42007; geodeticDatum: WGS84; georeferenceProtocol: GPS; **Identification:** identifiedBy: Do Van Tu; dateIdentified: 2020; **Event:** samplingProtocol: hand net; eventDate: 2011-7-30; year: 2011; month: 7; day: 30; **Record Level:** type: PhysicalObject; institutionCode: ZMB; collectionCode: ZMB; basisOfRecord: PreservedSpecimen
- **Type status:**Other material. **Occurrence:** catalogNumber: ZMB 29755; recordedBy: Chris Lukhaup; disposition: in collection; **Taxon:** scientificName: *Caridinalanceifrons* Yu, 1936; kingdom: Animal; phylum: Arthropoda; class: Malacostraca; order: Decapoda; family: Atyidae; genus: Caridina; specificEpithet: *lanceifrons*; taxonRank: Species; scientificNameAuthorship: Yu, 1936; vernacularName: atyid shrimp; nomenclaturalCode: ICZN; taxonomicStatus: accepted; **Location:** country: China; countryCode: CN; stateProvince: Guangdong; locality: Mountains nr Tangshan; decimalLatitude: 23.66126; decimalLongitude: 114.02508; geodeticDatum: WGS84; georeferenceProtocol: GPS; **Identification:** identifiedBy: Do Van Tu; dateIdentified: 2020; **Event:** samplingProtocol: hand net; eventDate: 2015-4-1; year: 2015; month: 4; day: 1; **Record Level:** type: PhysicalObject; institutionCode: ZMB; collectionCode: ZMB; basisOfRecord: PreservedSpecimen
- **Type status:**Other material. **Occurrence:** catalogNumber: ZMB 30684; recordedBy: Jens Kühne; disposition: in collection; **Taxon:** scientificName: *Caridinalanceifrons* Yu, 1936; kingdom: Animal; phylum: Arthropoda; class: Malacostraca; order: Decapoda; family: Atyidae; genus: Caridina; specificEpithet: *lanceifrons*; taxonRank: Species; scientificNameAuthorship: Yu, 1936; vernacularName: atyid shrimp; nomenclaturalCode: ICZN; taxonomicStatus: accepted; **Location:** country: Thailand; countryCode: TH; stateProvince: Nakhon Si Thammarat; locality: Sichon, main river; decimalLatitude: 9.00778; decimalLongitude: 99.82167; geodeticDatum: WGS84; georeferenceProtocol: GPS; **Identification:** identifiedBy: Do Van Tu; dateIdentified: 2020; **Event:** samplingProtocol: hand net; eventDate: 2017-2-1; year: 2017; month: 2; day: 1; **Record Level:** type: PhysicalObject; institutionCode: ZMB; collectionCode: ZMB; basisOfRecord: PreservedSpecimen
- **Type status:**Other material. **Occurrence:** catalogNumber: ZMB 32403; recordedBy: Jens Kühne; disposition: in collection; **Taxon:** scientificName: *Caridinalanceifrons* Yu, 1936; kingdom: Animal; phylum: Arthropoda; class: Malacostraca; order: Decapoda; family: Atyidae; genus: Caridina; specificEpithet: *lanceifrons*; taxonRank: Species; scientificNameAuthorship: Yu, 1936; vernacularName: atyid shrimp; nomenclaturalCode: ICZN; taxonomicStatus: accepted; **Location:** country: Thailand; countryCode: TH; stateProvince: Nakhon Si Thammarat; locality: River after limestone cave (-200m), Ban Wat Menao Wan (Ban Chang Klang), Amper Chandi; decimalLatitude: 8.33417; decimalLongitude: 99.65139; geodeticDatum: WGS84; georeferenceProtocol: GPS; **Identification:** identifiedBy: Do Van Tu; dateIdentified: 2020; **Event:** samplingProtocol: hand net; eventDate: 2015-3-1; year: 2015; month: 3; day: 1; **Record Level:** type: PhysicalObject; institutionCode: ZMB; collectionCode: ZMB; basisOfRecord: PreservedSpecimen
- **Type status:**Other material. **Occurrence:** catalogNumber: ZMB 32405; recordedBy: Jens Kühne; disposition: in collection; **Taxon:** scientificName: *Caridinalanceifrons* Yu, 1936; kingdom: Animal; phylum: Arthropoda; class: Malacostraca; order: Decapoda; family: Atyidae; genus: Caridina; specificEpithet: *lanceifrons*; taxonRank: Species; scientificNameAuthorship: Yu, 1936; vernacularName: atyid shrimp; nomenclaturalCode: ICZN; taxonomicStatus: accepted; **Location:** country: Thailand; countryCode: TH; stateProvince: Satun; locality: Amphoe Langhuu, near Manang; decimalLatitude: 6.9; decimalLongitude: 99.8; geodeticDatum: WGS84; georeferenceProtocol: GPS; **Identification:** identifiedBy: Do Van Tu; dateIdentified: 2020; **Event:** samplingProtocol: hand net; eventDate: 2012-1-1; year: 2012; month: 1; day: 1; **Record Level:** type: PhysicalObject; institutionCode: ZMB; collectionCode: ZMB; basisOfRecord: PreservedSpecimen

#### Description

**Material examined for description**. 1 female, cl 3.5 (ZMB 33800), Vietnam: Nam Dinh Province, Nghia Hung District, Nghia Son Commune, Quan Lieu, 20°11'25.619''N 106°10'49.109'' E, [= type locality of *Caridinaflavilineata* Dang, 1975], coll. Do Van Tu, 31 May 2020; 2 males, cl 3.5–3.6, 3 females, cl 4.6–5.6 (ZMB 33823), Vietnam: Vinh Phuc Province, Vinh Tuong District, 21°15'03.0"N 105°26'48.6"E, [= type locality of *Caridinavietriensis* Dang & Do, 2007], coll. Do Van Tu, 19 July 2020; 5 males, cl 2.6–5.0, 12 females, cl 2.8–5.3 (ZMB 30264), Vietnam: Da Nang City, Lang Van Commune, 16°10'4''N 108°8'32''E, [= near the type locality of *Caridinapseudoflavilineata* Do & Dang, 2010], coll. Do Van Tu, 5 May 2017.

**Cephalothorax and cephalic appendages.** Carapace length 2.6–5.3 mm. Rostrum straight, reaching near to or slightly beyond end of antennular peduncle, 0.45–0.96 (median 0.6) times as long as carapace, rostral formula: 4–6+10–16/1–5 (n = 10), teeth normal (Fig. 5a). Suborbital angle acute, completely fused with antennal spine; pterygostomian margin rounded (Fig. 5a). Eyes well developed with globular cornea, anterior end reaching to 0.7 times length of basal segment of antennular peduncle (Fig. 5a). Antennular peduncle 0.6–0.63 (median 0.61) times as long as carapace; basal segment 1.56–2.33 (median 2.0) times as long as second segment, second segment 1.2–1.67 (median 1.4) times as long as third segment (Fig. 5b). Stylocerite reaching to 0.72 times length of basal segment of antennular peduncle (Fig. 5b). Scaphocerite reaching beyond distal end of antennular peduncle, 3.0–4.33 (median 3.41) times as long as wide (Fig. 5c).

**Abdominal somites, telson and uropods.** Sixth abdominal somite 0.38–0.62 (median 0.44) times length of carapace, 1.36–1.64 (median 1.62) times as long as fifth abdominal somite, 0.6–1.0 (median 0.86) times length of telson. Telson length 2.33–3.85 (median 2.81) times as long as proximal width, distal margin triangular, terminating in a short median projection, with 5–6 pairs of dorsal spiniform setae and one pair of dorso-subdistal spiniform setae; distal end with 4–5 pairs of spiniform setae, lateral pair longer than intermediate pairs (Fig. 5d). Preanal carina high, with few setae, lacking spine. Uropodal diaeresis with 14–22 (median 16) movable spiniform setae, outermost one slightly shorter than lateral angle (Fig. 5e).

**Pereiopods.** Epipod present on first four pereiopods. First pereiopod short, robust, reaching to distal end of eyestalk; chela 1.89–2.4 (median 2.19) times as long as wide, 1.24–2.09 (median 1.44) times length of carpus; tips of fingers rounded, with hook; dactylus slightly longer than palm, 1.0–1.33 (median 1.11) times as long as palm; carpus excavated anteriorly, 1.22–2.0 (median 1.85) times as long as wide, 0.65–0.86 (median 0.8) times length of merus; merus 2.57–3.25 (median 2.86) times as long as wide, longer than ischium (Fig. 6a). Second pereiopod long, slender, reaching to middle of second segment of antennular peduncle; chela 2.46–3.29 (median 2.56) times as long as wide, 0.73–0.96 (median 0.85) times length of carpus; tips of fingers rounded, without hook; dactylus 1.09–1.89 (median 1.36) times as long as palm; carpus 4.0–5.0 (median 4.5) times as long as wide, 1.0–1.05 (median 1.04) times as long as merus; merus 4.2–5.25 (median 5.2) times as long as wide, longer than ischium (Fig. 6b). Third pereiopod slender, reaching to end of scaphocerite by its dactylus, terminating in two claws, with 5–7 accessory spiniform setae on flexor margin, dactylus 2.75–3.5 (median 2.9) times as long as wide (terminal claws and spiniform setae on flexor margin included), propodus 8.0–10.29 (median 9.0) times as long as wide, 3.25–5.29 (median 4.62) times as long as dactylus; carpus 3.75–5.0 (median 4.42) times as long as wide, 0.5–0.69 (median 0.58) times as long as propodus, 0.36–0.53 (median 0.47) times as long as merus; merus 5.83–7.33 (median 6.69) times as long as wide, bearing 3–5 strong, movable spiniform setae on posterior margin of outer surface; ischium with one movable spiniform seta (Fig. 6c). Fifth pereiopod slender, reaching to end of second segment of antennular peduncle, dactylus 2.33–3.5 (median 2.9) times as long as wide (terminal claw and spiniform setae on flexor margin included), terminating in one large claw, with 22–38 spiniform setae on flexor margin; propodus 12.0–14.0 (median 13.1) times as long as wide, 4.14–5.71 (median 4.8) times length of dactylus; carpus 4.33–6.25 (median 5.0) times as long as wide, 0.47–0.56 (median 0.49) times as long as propodus, 0.54–0.69 (median 0.64) times as long as merus; merus 5.33–6.73 (median 5.92) times as long as wide, bearing 3–4 strong, movable spiniform setae on posterior margin of outer surface (Fig. 6d).

**Pleopods.** Endopod of male first pleopod extending to 0.4 times length of exopod, subtriangular in shape, 2.09–2.88 (median 2.32) times as long as proximal width, rounded distally, long plumose setae on outer and distal margins, medium-length setae on inner margin; with appendix interna exceeding terminal margin of endopod by 0.5 times its length (Fig. 6e). Appendix masculina of male second pleopod slender, sub-cylindrical, reaching to proximal 0.6 times endopod length, 6.67 times as long as distal width, finger-shaped, with some short spiniform setae on outer surface and some long spiniform setae on distal surface; appendix interna at the middle of appendix masculina, narrow, small, extending about 0.5 times length of appendix masculina (Fig. 6f).

**Colouration.** Body yellowish (Fig. 7a), females sometimes black or dark green with dorsal yellow stripe (Fig. 7b).

#### Distribution

This species is widely distributed in northern Vietnam (no further south than Quang Nam Province in central Vietnam). In China, the species were found in Fujian, Guangdong, Guangxi, Hainan and Hunan (Liang 2004). Our study also provides the first record for *Caridinalanceifrons* from Thailand, where it has been found in the southern Provinces of Nakhon Si Thammarat and Satun (Fig. 1b). This apparently disjunct distribution seems indicative of a major sampling gap in Thailand, Laos and potentially Cambodia. However, the absence of this species from southern Vietnam is unlikely to be an artefact, as this part of the country has been surveyed extensively by the first author. This is the species with the widest distribution range when compared to other land-locked species in Vietnam (Fig. 1b).

#### Ecology

This species inhabits mountain streams and rivers. We could not collect any specimens of this species from estuarine environments. In addition, based on egg-size (Table 1), we consider it as a land-locked species.

#### Taxon discussion

The populations in Vietnam showed no significant difference when compared to the original description of *Caridinalanceifrons* Yu, 1936 in Yu (1936) or the re-descriptions by Liang (2004) and Cai (2014).

Three species described from Vietnam have been considered synonyms of *Caridinalanceifrons*:

According to Li and Liang (2002), *Caridinaflavilineata* Dang, 1975 is a junior synonym of *Caridinalanceifrons* Yu, 1936. Dang and Do (2008) and Dang and Ho (2012) commented that, although *C.flavilineata* is very close to *C.lanceifrons*, they can be distinguished by differences in some morphological characteristics, especially in body colour, egg size, appendix masculina, appendix interna and rostrum. By examination of many specimens of *C.flavilineata* collected throughout North Vietnam, including from the area of the type locality (ZMB 30390, ZMB 33800), we found that a black or dark green body colour, especially a yellow stripe, is only present in females and is not consistently found in every population; the egg size is similar to *C.lanceifrons* (0.9–0.95 × 0.5–0.6 compared to 0.84–0.9 × 0.56–0.62), the appendix masculina, the appendix interna and the rostrum did not show any significant differences. After checking many specimens from Vietnam, Liang (2004) also stated that there is no difference between these species. *Caridinalanceifrons* is very common in south China, especially in Hainan, Guangdong, Guangxi and (presumably) Fujian (Liang 2004). In the Longzhou River that flows through North Vietnam, this species is abundant. Therefore, it can move upstream and is widely distributed throughout North Vietnam. Based on specimens collected from many sites in Vietnam and the descriptions by Dang (1975) and Yu (1936), we agree with Li and Liang (2002) and Liang (2004) and Cai (2014) that *C.flavilineata* Dang, 1975 is a junior synonym of *Caridinalanceifrons* Yu, 1936.

Dang and Do (2007a) described *Caridinavietriensis* from North Vietnam. According to these authors, this species differs from *C.flavilineata* in the rostrum, the endopod of the male’s first pleopod and body colour. However, they did not point out these differences clearly. They also did not compare *Caridinavietriensis* with *C.lanceifrons*. Based on the descriptions given by Dang and Do (2007a) and examination of specimens (ZMB 33823) collected from Phu Tho Province, the area of the type locality and surrounding zones, we here consider *C.vietriensis* as a synonym of *C.lanceifrons*.

Do and Dang (2010) also described *Caridinapseudoflavilineata* from the same locality as *C.haivanensis* Do & Dang, 2010 at Hai Van mountain pass. Unfortunately, we could not find this species again at the type locality during two of our surveys. These authors did not compare *C.pseudoflavilineata* with *C.lanceifrons*, but they compared it with *C.flavilineata.* They suggested that *C.pseudoflavilineata* can be distinguished from *C.flavilineata* by the shorter and sharper rostrum, the higher number of spiniform setae on the dactylus of the fifth pereiopod and uropodal diaeresis, the shape of endopod of male first pleopod and the larger eggs. However, based on the description and illustrations by these authors and the examination of specimens collected at some distance from the type localities, Thua Thien-Hue, Da Nang and Quang Nam, we here conclude that *Caridinapseudoflavilineata* Do & Dang, 2010 is identical to *C.lanceifrons*. The rostrum reaching to the end of the third segment of antennular peduncle with a sharpened tip, the subtriangular endopod of male first pleopod, the dactylus of the fifth pereiopod with 25–28 spiniform setae and uropodal diaeresis with 14–15 movable spiniform setae are the characteristics of *C.lanceifrons* (Do and Dang (2010): figs. 3–4, cf. Yu (1936): figs. 5–6, Liang (2004): fig. 128, Cai (2014): Fig. 6). There is not any obvious character distinguishing these two species, except that the eggs of *C.lanceifrons* are slightly smaller than those of *C.pseudoflavilineata* (0.9–0.8 × 0.5–0.6 mm vs. 1.1 × 0.5 mm).

### Caridina serrata

Stimpson, 1860

DEB3F040-71F0-5270-8CE2-AA6D745099A9

Caridina serrata Stimpson, 1860: 29. [Type locality: hill above Bekhers, Hong Kong Island, Hong Kong, China; neotype designation by Cai and Ng (1999)].
Caridina serrata — Ortmann (1894): 406; Bouvier (1905): 76; Bouvier (1925): 258, fig. 593; Kemp (1918): 289, fig. 12; Cai and Ng (1999): figs. 2, 3, 6B and 6G.
Caridina serrata *Caridina serrata serrata *Dang et al. (1980)
Caridina serrata *Caridina serrata *Liang and Yan (1983)Liang and Yan (1985)Liang and Yan (1986)Dudgeon (1985)Dudgeon (1987)Hung et al. (1993)Shy and Yu (1998)

#### Materials

- **Type status:**Other material. **Occurrence:** catalogNumber: ZMB 30306; recordedBy: Pham The Cuong; disposition: in collection; **Taxon:** scientificName: *Caridinaserrata* Stimpson, 1860; kingdom: Animal; phylum: Arthropoda; class: Malacostraca; order: Decapoda; family: Atyidae; genus: Caridina; specificEpithet: *serrata*; taxonRank: Species; scientificNameAuthorship: Stimpson, 1860; vernacularName: atyid shrimp; nomenclaturalCode: ICZN; taxonomicStatus: accepted; **Location:** island: Cu Lao Cham; country: Vietnam; countryCode: VN; stateProvince: Quang Nam; county: Hoi An; locality: Stream running into reservoir, Cu Lao Cham Island; decimalLatitude: 15.94303; decimalLongitude: 108.52297; geodeticDatum: WGS84; georeferenceProtocol: GPS; **Identification:** identifiedBy: Do Van Tu; dateIdentified: 2020; **Event:** samplingProtocol: hand net; eventDate: 2017-5-10; year: 2017; month: 5; day: 10; **Record Level:** type: PhysicalObject; institutionCode: ZMB; collectionCode: ZMB; basisOfRecord: PreservedSpecimen
- **Type status:**Other material. **Occurrence:** catalogNumber: OUMNH 201307023; recordedBy: Werner & Maria Klotz; disposition: in collection; **Taxon:** scientificName: *Caridinaserrata* Stimpson, 1860; kingdom: Animal; phylum: Arthropoda; class: Malacostraca; order: Decapoda; family: Atyidae; genus: Caridina; specificEpithet: *serrata*; taxonRank: Species; scientificNameAuthorship: Stimpson, 1860; vernacularName: atyid shrimp; nomenclaturalCode: ICZN; taxonomicStatus: accepted; **Location:** island: Hong Kong; country: China; countryCode: CN; stateProvince: ﻿Hong Kong; locality: Lantau Island, middle course of river; decimalLatitude: 22.2267; decimalLongitude: 113.8801; geodeticDatum: WGS84; georeferenceProtocol: GPS; **Identification:** identifiedBy: Do Van Tu; dateIdentified: 2020; **Event:** samplingProtocol: hand net; eventDate: 2011-3-18; year: 2011; month: 3; day: 18; **Record Level:** type: PhysicalObject; institutionCode: OUMNH; collectionCode: OUMNH; basisOfRecord: PreservedSpecimen
- **Type status:**Other material. **Occurrence:** catalogNumber: ZMB 31764; recordedBy: Christoph D. Schubart, Larissa Kawasch, Brian H.Y. Ip; disposition: in collection; **Taxon:** scientificName: *Caridinaserrata* Stimpson, 1860; kingdom: Animal; phylum: Arthropoda; class: Malacostraca; order: Decapoda; family: Atyidae; genus: Caridina; specificEpithet: *serrata*; taxonRank: Species; scientificNameAuthorship: Stimpson, 1860; vernacularName: atyid shrimp; nomenclaturalCode: ICZN; taxonomicStatus: accepted; **Location:** island: Hong Kong; country: China; countryCode: CN; stateProvince: ﻿Hong Kong; locality: Lantau Island, Pui O Sanwai Tsuen; decimalLatitude: 22.2452; decimalLongitude: 113.9769; geodeticDatum: WGS84; georeferenceProtocol: GPS; **Identification:** identifiedBy: Do Van Tu; dateIdentified: 2020; **Event:** samplingProtocol: hand net; eventDate: 2013-2-26; year: 2013; month: 2; day: 26; **Record Level:** type: PhysicalObject; institutionCode: ZMB; collectionCode: ZMB; basisOfRecord: PreservedSpecimen
- **Type status:**Other material. **Occurrence:** catalogNumber: ZMB 32189; recordedBy: Werner Klotz; disposition: in collection; **Taxon:** scientificName: *Caridinaserrata* Stimpson, 1860; kingdom: Animal; phylum: Arthropoda; class: Malacostraca; order: Decapoda; family: Atyidae; genus: Caridina; specificEpithet: *serrata*; taxonRank: Species; scientificNameAuthorship: Stimpson, 1860; vernacularName: atyid shrimp; nomenclaturalCode: ICZN; taxonomicStatus: accepted; **Location:** island: Hong Kong; country: China; countryCode: CN; stateProvince: ﻿Hong Kong; locality: Tributary of the Pok Fu Lam Reservoir, close to mouth into reservoir; decimalLatitude: 22.2662; decimalLongitude: 114.1422; geodeticDatum: WGS84; georeferenceProtocol: GPS; **Identification:** identifiedBy: Do Van Tu; dateIdentified: 2020; **Event:** samplingProtocol: hand net; eventDate: 2009-4-6; year: 2009; month: 4; day: 6; **Record Level:** type: PhysicalObject; institutionCode: ZMB; collectionCode: ZMB; basisOfRecord: PreservedSpecimen
- **Type status:**Other material. **Occurrence:** catalogNumber: ZMB 29473; recordedBy: Yixiong Cai; disposition: in collection; **Taxon:** scientificName: *Caridinaserrata* Stimpson, 1860; kingdom: Animal; phylum: Arthropoda; class: Malacostraca; order: Decapoda; family: Atyidae; genus: Caridina; specificEpithet: *serrata*; taxonRank: Species; scientificNameAuthorship: Stimpson, 1860; vernacularName: atyid shrimp; nomenclaturalCode: ICZN; taxonomicStatus: accepted; **Location:** country: China; countryCode: CN; stateProvince: Guangdong; locality: Xiasliao stream, near Xishan Town, Nan'ao County; decimalLatitude: 23.44215; decimalLongitude: 116.99551; geodeticDatum: WGS84; georeferenceProtocol: GPS; **Identification:** identifiedBy: Do Van Tu; dateIdentified: 2020; **Event:** samplingProtocol: hand net; eventDate: 2005-2-5; year: 2005; month: 2; day: 5; **Record Level:** type: PhysicalObject; institutionCode: ZMB; collectionCode: ZMB; basisOfRecord: PreservedSpecimen
- **Type status:**Other material. **Occurrence:** catalogNumber: ZMB 32190; recordedBy: Chris Lukhaup; disposition: in collection; **Taxon:** scientificName: *Caridinaserrata* Stimpson, 1860; kingdom: Animal; phylum: Arthropoda; class: Malacostraca; order: Decapoda; family: Atyidae; genus: Caridina; specificEpithet: *serrata*; taxonRank: Species; scientificNameAuthorship: Stimpson, 1860; vernacularName: atyid shrimp; nomenclaturalCode: ICZN; taxonomicStatus: accepted; **Location:** island: Hong Kong; country: China; countryCode: CN; stateProvince: ﻿Hong Kong; locality: Lantau Island, North side; decimalLatitude: 22.26679; decimalLongitude: 113.93352; geodeticDatum: WGS84; georeferenceProtocol: GPS; **Identification:** identifiedBy: Do Van Tu; dateIdentified: 2020; **Event:** samplingProtocol: hand net; eventDate: 2009-8-9; year: 2009; month: 8; day: 9; **Record Level:** type: PhysicalObject; institutionCode: ZMB; collectionCode: ZMB; basisOfRecord: PreservedSpecimen
- **Type status:**Other material. **Occurrence:** catalogNumber: ZMB 32306; recordedBy: Shen, C.J. (1948); disposition: in collection; **Taxon:** scientificName: *Caridinaserrata* Stimpson, 1860; kingdom: Animal; phylum: Arthropoda; class: Malacostraca; order: Decapoda; family: Atyidae; genus: Caridina; specificEpithet: *serrata*; taxonRank: Species; scientificNameAuthorship: Stimpson, 1860; vernacularName: atyid shrimp; nomenclaturalCode: ICZN; taxonomicStatus: accepted; **Location:** island: Hong Kong; country: China; countryCode: CN; stateProvince: ﻿Hong Kong; locality: Lantau Island, upper course of river; decimalLatitude: 22.26214; decimalLongitude: 113.96688; geodeticDatum: WGS84; georeferenceProtocol: GPS; **Identification:** identifiedBy: Do Van Tu; dateIdentified: 2020; **Event:** samplingProtocol: hand net; eventDate: 2011-1-11; year: 2011; month: 1; day: 11; **Record Level:** type: PhysicalObject; institutionCode: ZMB; collectionCode: ZMB; basisOfRecord: PreservedSpecimen

#### Description

**Material examined for description**. 10 males, cl 3.1–4.7, 5 females, cl 4.0–4.6 (ZMB 30306), Vietnam: Quang Nam Province, Hoi An City, Cu Lao Cham Island, a small stream running into the reservoir, 15°56'34.9''N 108°31'22.7''E, coll. Pham The Cuong, 10 May 2017.

**Cephalothorax and cephalic appendages.** Carapace length 3.1–4.7 mm. Rostrum short, straight or slightly curved downwards, reaching to the end of basal segment of antennular peduncle, 0.38–0.46 (median 0.43) times as long as carapace, rostral formula: 0–4+3–10/0–2 (n = 10), teeth normal (Fig. 8a). Suborbital angle acute, completely fused with antennal spine; pterygostomian margin rounded (Fig. 8a). Eyes well developed with globular cornea, anterior end reaching to 0.6 times length of basal segment of antennular peduncle (Fig. 8a). Antennular peduncle 0.48–0.51 (median 0.5) times as long as carapace; basal segment 1.83–2.0 (median 1.83) times as long as second segment, second segment 1.2–1.5 (median 1.33) times as long as third segment (Fig. 8b). Stylocerite reaching to about middle (0.4 times length) of second segment of antennular peduncle (Fig. 8b). Scaphocerite reaching beyond distal end of antennular peduncle, 3.57–3.64 (median 3.57) times as long as wide (Fig. 8c).

**Abdominal somites, telson and uropods.** Sixth abdominal somite 0.41–0.49 (median 0.42) times length of carapace, 1.44–1.67 (median 1.52) times as long as fifth abdominal somite, 0.85–1.0 (median 0.9) times length of telson. Telson length 2.14–2.5 (median 2.36) times as long as proximal width, distal margin triangular, terminating in a short median projection, with 4–5 pairs of dorsal spiniform setae and one pair of dorso-subdistal spiniform setae; distal end with 4 pairs of spiniform setae, lateral pair slightly longer than intermediate pairs (Fig. 8d). Pre-anal carina rounded, with few setae, lacking tooth or spine. Uropodal diaeresis with 20–22 movable spiniform setae, outermost one slightly shorter than lateral angle (Fig. 8e).

**Pereiopods.** Epipod present on first four pereiopods. First pereiopod short, robust, reaching beyond end of basal segment of antennular peduncle; chela 1.69–2.0 (median 1.92) times as long as wide, 1.39–1.56 (median 1.47) times length of carpus; tips of fingers rounded, with hook; dactylus shorter than palm, 0.67–0.92 (median 0.75) times as long as palm; carpus excavated anteriorly, 1.67–1.80 (median 1.73) times as long as wide, 0.78–0.9 (median 0.79) times length of merus; merus 2.11–2.56 (median 2.22) times as long as wide, longer than ischium (Fig. 9a). Second pereiopod long, slender, reaching beyond end of scaphocerite; chela 2.25–2.79 (median 2.56) times as long as wide, 0.58–0.9 (median 0.64) times length of carpus; tips of fingers rounded, without hook; dactylus 1.33–1.57 (median 1.5) times as long as palm; carpus 5.0–6.67 (median 5.78) times as long as wide, 1.14–1.13 (median 1.14) times as long as merus; merus 5.11–5.83 (median 5.75) times as long as wide, longer than ischium (Fig. 9b). Third pereiopod slender, reaching beyond end of scaphocerite by its dactylus, terminating in two claws, with four to five accessory spiniform setae on flexor margin, dactylus 3.0–4.0 (median 3.5) times as long as wide (terminal claw and spiniform setae on flexor margin included), propodus 9.0–9.7 (median 9.0) times as long as wide, 3.75–4.86 (median 3.75) times as long as dactylus; carpus 4.2–5.0 (median 4.43) times as long as wide, 0.62–0.78 (median 0.69) times as long as propodus, 0.46–0.64 (median 0.57) times as long as merus; merus 5.5–6.57 (median 6.0) times as long as wide, bearing four strong, movable spiniform setae on posterior margin of outer surface; ischium with one movable spiniform seta (Fig. 9c). Fifth pereiopod slender, reaching to end of basal segment of antennular peduncle, dactylus 2.17–2.67 (median 2.42) times as long as wide (terminal claw and spiniform setae on flexor margin included), terminating in one large claw, with 37–42 spiniform setae on flexor margin; propodus 10–12 (median 10.72) times as long as wide, 6.25–8.31 (median 7.17) times length of dactylus; carpus 3.8–5.0 (median 4.5) times as long as wide, 0.36–0.46 (median 0.42) times as long as propodus, 0.51–0.65 (median 0.61) times as long as merus; merus 5.83–6.2 (median 5.59) times as long as wide, bearing 3–4 strong, movable spiniform setae on posterior margin of outer surface (Fig. 9d).

**Pleopods.** Endopod of male first pleopod extending to 0.7 times length of exopod, sub-rectangular in shape, 2.45–2.75 (median 2.6) times as long as proximal width, inner margin concave, outer margin slightly convex, rounded distally, long plumose setae on outer and distal margins, medium-length setae on inner margin; with appendix interna exceeding terminal margin of endopod by 0.4 times its length (Fig. 9e). Appendix masculina of male second pleopod slender, finger-shaped, not widened distally, reaching to proximal 0.7 times endopod length, 3.8 times as long as distal width, some short spiniform setae on outer surface and some long spiniform setae on distal surface; appendix interna at the middle of appendix masculina, extending about 0.5 times length of appendix masculina (Fig. 9f).

#### Diagnosis

##### Distribution

*Caridinaserrata* Stimpson, 1860 was recorded from Hong Kong Island only by Cai and Ng (1999), but was later also reported from Lantau island, Hong Kong and (as *C.nanaoensis*) from Nanao Island, China (Klotz and von Rintelen 2014). Here, we present the first record of this species in Cu Lao Cham Island from central Vietnam (Fig. 1c).

##### Ecology

Although this species up to now was only recorded from streams on small islands (which are on the continental shelf, i.e. would have been connected to the mainland during glacial maxima; see, for example, Voris 2000), it can be considered a land-locked species, based on egg-size (Table 1).

#### Taxon discussion

Dang et al. (1980) recorded two subspecies of *Caridinaserrata*, namely *C.serrataserrata* and *C.serratacucphuongensis*. In 1999, Cai and Ng revised the *C.serrata* species group and stated that *Caridinaserrataserrata* Dang, 1980 does not belong to this species because of the length of the rostrum and it is also not a member of the *C.serrata* species group as the stylocerite is not over-reaching the basal segment of the antennular peduncle. *Caridinaserratacucphuongensis* was discussed as being different from *C.serrata* (Cai and Ng 1999) and was elevated to full species rank (Cai et al. 1999). Dang and Do (2007a) described *C.pseudoserrata* from North Vietnam and assigned the specimens identified as *C.serrataserrata* by Dang et al. (1980) to their new species.

### Neocaridina palmata palmata

(Shen, 1948)

69587305-2D33-5B3F-A5D2-502005AC763D

Caridina palmata Shen, 1948: 120, plate 12, fig. j. [Type locality: Sha-ping-pa, Chungking, China].
Caridina elongata Shen, 1948: 121, plate 13, figs. a–e. [Kweilin, China]
Caridina denticulata vietnamensis Dang, 1967: 157, fig. 4. [Cao Bang and Lang Son provinces, Vietnam].
Caridina vietnamensis — Dang (1975): 72; Dang in Dang et al. (1980): 410–412, fig. 234.
Caridina denticulata — Liang and Yan (1986): 197; Liang et al. (1993): 41; Liang and Zhou (1993): 231.
Neocaridina palmata palmata — Cai (1996): 144.
Neocaridina palmata — Li (1997): 455; Li and Liang (2002): 708.
Neocaridina vietnamensis — Dang and Do (2008): 8; Dang and Ho (2012): 159–163, fig. 55.

#### Materials

- **Type status:**Other material. **Occurrence:** catalogNumber: ZMB 29649; recordedBy: Do Van Tu; disposition: in collection; **Taxon:** scientificName: Neocaridinapalmatapalmata (Shen, 1948); kingdom: Animal; phylum: Arthropoda; class: Malacostraca; order: Decapoda; family: Atyidae; genus: Neocaridina; specificEpithet: palmata; infraspecificEpithet: palmata; taxonRank: Subspecies; scientificNameAuthorship: (Shen, 1948); vernacularName: atyid shrimp; nomenclaturalCode: ICZN; taxonomicStatus: accepted; **Location:** country: Vietnam; countryCode: VN; stateProvince: Cao Bang; county: Hoa An; municipality: Hoa An; decimalLatitude: 22.66519; decimalLongitude: 106.17389; geodeticDatum: WGS84; georeferenceProtocol: GPS; **Identification:** identifiedBy: Do Van Tu; dateIdentified: 2020; **Event:** samplingProtocol: hand net; eventDate: 11/13/2012; year: 2012; month: 11; day: 13; **Record Level:** type: PhysicalObject; institutionCode: ZMB; collectionCode: ZMB; basisOfRecord: PreservedSpecimen
- **Type status:**Other material. **Occurrence:** catalogNumber: ZMB 29653; recordedBy: Do Van Tu; disposition: in collection; **Taxon:** scientificName: Neocaridinapalmatapalmata (Shen, 1948); kingdom: Animal; phylum: Arthropoda; class: Malacostraca; order: Decapoda; family: Atyidae; genus: Neocaridina; specificEpithet: palmata; infraspecificEpithet: palmata; taxonRank: Subspecies; scientificNameAuthorship: (Shen, 1948); vernacularName: atyid shrimp; nomenclaturalCode: ICZN; taxonomicStatus: accepted; **Location:** country: Vietnam; countryCode: VN; stateProvince: Cao Bang; county: Ha Quang; municipality: Truong Ha; decimalLatitude: 22.947; decimalLongitude: 106.04167; geodeticDatum: WGS84; georeferenceProtocol: GPS; **Identification:** identifiedBy: Do Van Tu; dateIdentified: 2020; **Event:** samplingProtocol: hand net; eventDate: 11/13/2012; year: 2012; month: 11; day: 13; **Record Level:** type: PhysicalObject; institutionCode: ZMB; collectionCode: ZMB; basisOfRecord: PreservedSpecimen
- **Type status:**Other material. **Occurrence:** catalogNumber: ZMB 29685; recordedBy: Jens Kühne; disposition: in collection; **Taxon:** scientificName: Neocaridinapalmatapalmata (Shen, 1948); kingdom: Animal; phylum: Arthropoda; class: Malacostraca; order: Decapoda; family: Atyidae; genus: Neocaridina; specificEpithet: palmata; infraspecificEpithet: palmata; taxonRank: Subspecies; scientificNameAuthorship: (Shen, 1948); vernacularName: atyid shrimp; nomenclaturalCode: ICZN; taxonomicStatus: accepted; **Location:** country: Vietnam; countryCode: VN; stateProvince: Lang Son; decimalLatitude: 21.54556; decimalLongitude: 106.38611; geodeticDatum: WGS84; georeferenceProtocol: GPS; **Identification:** identifiedBy: Do Van Tu; dateIdentified: 2020; **Event:** samplingProtocol: hand net; **Record Level:** type: PhysicalObject; institutionCode: ZMB; collectionCode: ZMB; basisOfRecord: PreservedSpecimen
- **Type status:**Other material. **Occurrence:** catalogNumber: ZMB 30251; recordedBy: Do Van Tu; disposition: in collection; **Taxon:** scientificName: Neocaridinapalmatapalmata (Shen, 1948); kingdom: Animal; phylum: Arthropoda; class: Malacostraca; order: Decapoda; family: Atyidae; genus: Neocaridina; specificEpithet: palmata; infraspecificEpithet: palmata; taxonRank: Subspecies; scientificNameAuthorship: (Shen, 1948); vernacularName: atyid shrimp; nomenclaturalCode: ICZN; taxonomicStatus: accepted; **Location:** country: Vietnam; countryCode: VN; stateProvince: Cao Bang; county: Ha Quang; municipality: Truong Ha; locality: Pac Ma stream behind Hai Hoa motel, Pac Bo; decimalLatitude: 22.97331; decimalLongitude: 106.05269; geodeticDatum: WGS84; georeferenceProtocol: GPS; **Identification:** identifiedBy: Do Van Tu; dateIdentified: 2020; **Event:** samplingProtocol: hand net; eventDate: 05/24/2017; year: 2017; month: 5; day: 24; **Record Level:** type: PhysicalObject; institutionCode: ZMB; collectionCode: ZMB; basisOfRecord: PreservedSpecimen
- **Type status:**Other material. **Occurrence:** catalogNumber: ZMB 30256; recordedBy: Do Van Tu; disposition: in collection; **Taxon:** scientificName: Neocaridinapalmatapalmata (Shen, 1948); kingdom: Animal; phylum: Arthropoda; class: Malacostraca; order: Decapoda; family: Atyidae; genus: Neocaridina; specificEpithet: palmata; infraspecificEpithet: palmata; taxonRank: Subspecies; scientificNameAuthorship: (Shen, 1948); vernacularName: atyid shrimp; nomenclaturalCode: ICZN; taxonomicStatus: accepted; **Location:** country: Vietnam; countryCode: VN; stateProvince: Cao Bang; county: Ha Quang; municipality: Truong Ha; locality: Pac Ma stream behind Hai Hoa motel, Pac Bo; decimalLatitude: 22.97331; decimalLongitude: 106.05269; geodeticDatum: WGS84; georeferenceProtocol: GPS; **Identification:** identifiedBy: Do Van Tu; dateIdentified: 2020; **Event:** samplingProtocol: hand net; eventDate: 05/24/2017; year: 2017; month: 5; day: 24; **Record Level:** type: PhysicalObject; institutionCode: ZMB; collectionCode: ZMB; basisOfRecord: PreservedSpecimen
- **Type status:**Other material. **Occurrence:** catalogNumber: ZMB 30272; recordedBy: Do Van Tu; disposition: in collection; **Taxon:** scientificName: Neocaridinapalmatapalmata (Shen, 1948); kingdom: Animal; phylum: Arthropoda; class: Malacostraca; order: Decapoda; family: Atyidae; genus: Neocaridina; specificEpithet: palmata; infraspecificEpithet: palmata; taxonRank: Subspecies; scientificNameAuthorship: (Shen, 1948); vernacularName: atyid shrimp; nomenclaturalCode: ICZN; taxonomicStatus: accepted; **Location:** country: Vietnam; countryCode: VN; stateProvince: Cao Bang; county: Ha Quang; municipality: Lung Nam; locality: Stream on the road to Thin Tang village; decimalLatitude: 22.97611; decimalLongitude: 106.06863; geodeticDatum: WGS84; georeferenceProtocol: GPS; **Identification:** identifiedBy: Do Van Tu; dateIdentified: 2020; **Event:** samplingProtocol: hand net; eventDate: 05/25/2017; year: 2017; month: 5; day: 25; **Record Level:** type: PhysicalObject; institutionCode: ZMB; collectionCode: ZMB; basisOfRecord: PreservedSpecimen
- **Type status:**Other material. **Occurrence:** catalogNumber: ZMB 30278; recordedBy: Do Van Tu; disposition: in collection; **Taxon:** scientificName: Neocaridinapalmatapalmata (Shen, 1948); kingdom: Animal; phylum: Arthropoda; class: Malacostraca; order: Decapoda; family: Atyidae; genus: Neocaridina; specificEpithet: palmata; infraspecificEpithet: palmata; taxonRank: Subspecies; scientificNameAuthorship: (Shen, 1948); vernacularName: atyid shrimp; nomenclaturalCode: ICZN; taxonomicStatus: accepted; **Location:** country: Vietnam; countryCode: VN; stateProvince: Tuyen Quang; county: Lam Binh; municipality: Thuong Lam; decimalLatitude: 22.49585; decimalLongitude: 105.30359; geodeticDatum: WGS84; georeferenceProtocol: GPS; **Identification:** identifiedBy: Do Van Tu; dateIdentified: 2020; **Event:** samplingProtocol: hand net; eventDate: 08/31/2017; year: 2017; month: 8; day: 31; **Record Level:** type: PhysicalObject; institutionCode: ZMB; collectionCode: ZMB; basisOfRecord: PreservedSpecimen
- **Type status:**Other material. **Occurrence:** catalogNumber: ZMB 30281; recordedBy: Do Van Tu; disposition: in collection; **Taxon:** scientificName: Neocaridinapalmatapalmata (Shen, 1948); kingdom: Animal; phylum: Arthropoda; class: Malacostraca; order: Decapoda; family: Atyidae; genus: Neocaridina; specificEpithet: palmata; infraspecificEpithet: palmata; taxonRank: Subspecies; scientificNameAuthorship: (Shen, 1948); vernacularName: atyid shrimp; nomenclaturalCode: ICZN; taxonomicStatus: accepted; **Location:** country: Vietnam; countryCode: VN; stateProvince: Cao Bang; county: Ha Quang; municipality: Lung Nam; locality: Stream on the road to Thin Tang village; decimalLatitude: 22.97611; decimalLongitude: 106.06863; geodeticDatum: WGS84; georeferenceProtocol: GPS; **Identification:** identifiedBy: Do Van Tu; dateIdentified: 2020; **Event:** samplingProtocol: hand net; eventDate: 05/25/2017; year: 2017; month: 5; day: 25; **Record Level:** type: PhysicalObject; institutionCode: ZMB; collectionCode: ZMB; basisOfRecord: PreservedSpecimen
- **Type status:**Other material. **Occurrence:** catalogNumber: ZMB 30289; recordedBy: Do Van Tu; disposition: in collection; **Taxon:** scientificName: Neocaridinapalmatapalmata (Shen, 1948); kingdom: Animal; phylum: Arthropoda; class: Malacostraca; order: Decapoda; family: Atyidae; genus: Neocaridina; specificEpithet: palmata; infraspecificEpithet: palmata; taxonRank: Subspecies; scientificNameAuthorship: (Shen, 1948); vernacularName: atyid shrimp; nomenclaturalCode: ICZN; taxonomicStatus: accepted; **Location:** country: Vietnam; countryCode: VN; stateProvince: Tuyen Quang; county: Lam Binh; municipality: Thuong Lam; decimalLatitude: 22.49585; decimalLongitude: 105.30359; geodeticDatum: WGS84; georeferenceProtocol: GPS; **Identification:** identifiedBy: Do Van Tu; dateIdentified: 2020; **Event:** samplingProtocol: hand net; eventDate: 08/31/2017; year: 2017; month: 8; day: 31; **Record Level:** type: PhysicalObject; institutionCode: ZMB; collectionCode: ZMB; basisOfRecord: PreservedSpecimen
- **Type status:**Other material. **Occurrence:** catalogNumber: ZMB 30291; recordedBy: Do Van Tu; disposition: in collection; **Taxon:** scientificName: Neocaridinapalmatapalmata (Shen, 1948); kingdom: Animal; phylum: Arthropoda; class: Malacostraca; order: Decapoda; family: Atyidae; genus: Neocaridina; specificEpithet: palmata; infraspecificEpithet: palmata; taxonRank: Subspecies; scientificNameAuthorship: (Shen, 1948); vernacularName: atyid shrimp; nomenclaturalCode: ICZN; taxonomicStatus: accepted; **Location:** country: Vietnam; countryCode: VN; stateProvince: Cao Bang; county: Trung Khanh; locality: Ban Gioc waterfall; decimalLatitude: 22.85298; decimalLongitude: 106.72369; geodeticDatum: WGS84; georeferenceProtocol: GPS; **Identification:** identifiedBy: Do Van Tu; dateIdentified: 2020; **Event:** samplingProtocol: hand net; eventDate: 05/26/2017; year: 2017; month: 5; day: 26; **Record Level:** type: PhysicalObject; institutionCode: ZMB; collectionCode: ZMB; basisOfRecord: PreservedSpecimen
- **Type status:**Other material. **Occurrence:** catalogNumber: ZMB 30300; recordedBy: Do Van Tu; disposition: in collection; **Taxon:** scientificName: Neocaridinapalmatapalmata (Shen, 1948); kingdom: Animal; phylum: Arthropoda; class: Malacostraca; order: Decapoda; family: Atyidae; genus: Neocaridina; specificEpithet: palmata; infraspecificEpithet: palmata; taxonRank: Subspecies; scientificNameAuthorship: (Shen, 1948); vernacularName: atyid shrimp; nomenclaturalCode: ICZN; taxonomicStatus: accepted; **Location:** country: Vietnam; countryCode: VN; stateProvince: Cao Bang; county: Cao Bang; locality: Bang Rriver, in Cao Bang city; decimalLatitude: 22.66503; decimalLongitude: 106.26267; geodeticDatum: WGS84; georeferenceProtocol: GPS; **Identification:** identifiedBy: Do Van Tu; dateIdentified: 2020; **Event:** samplingProtocol: hand net; eventDate: 05/25/2017; year: 2017; month: 5; day: 25; **Record Level:** type: PhysicalObject; institutionCode: ZMB; collectionCode: ZMB; basisOfRecord: PreservedSpecimen
- **Type status:**Other material. **Occurrence:** catalogNumber: ZMB 30302; recordedBy: Do Van Tu; disposition: in collection; **Taxon:** scientificName: Neocaridinapalmatapalmata (Shen, 1948); kingdom: Animal; phylum: Arthropoda; class: Malacostraca; order: Decapoda; family: Atyidae; genus: Neocaridina; specificEpithet: palmata; infraspecificEpithet: palmata; taxonRank: Subspecies; scientificNameAuthorship: (Shen, 1948); vernacularName: atyid shrimp; nomenclaturalCode: ICZN; taxonomicStatus: accepted; **Location:** country: Vietnam; countryCode: VN; stateProvince: Cao Bang; county: Cao Bang; locality: Bang Rriver, in Cao Bang city; decimalLatitude: 22.66503; decimalLongitude: 106.26267; geodeticDatum: WGS84; georeferenceProtocol: GPS; **Identification:** identifiedBy: Do Van Tu; dateIdentified: 2020; **Event:** samplingProtocol: hand net; eventDate: 05/25/2017; year: 2017; month: 5; day: 25; **Record Level:** type: PhysicalObject; institutionCode: ZMB; collectionCode: ZMB; basisOfRecord: PreservedSpecimen
- **Type status:**Other material. **Occurrence:** catalogNumber: ZMB 30303; recordedBy: Do Van Tu; disposition: in collection; **Taxon:** scientificName: Neocaridinapalmatapalmata (Shen, 1948); kingdom: Animal; phylum: Arthropoda; class: Malacostraca; order: Decapoda; family: Atyidae; genus: Neocaridina; specificEpithet: palmata; infraspecificEpithet: palmata; taxonRank: Subspecies; scientificNameAuthorship: (Shen, 1948); vernacularName: atyid shrimp; nomenclaturalCode: ICZN; taxonomicStatus: accepted; **Location:** country: Vietnam; countryCode: VN; stateProvince: Cao Bang; county: Trung Khanh; municipality: Dam Thuy; locality: Stream running out of cave, Ban Gun-Khuoi Ky; decimalLatitude: 22.84524; decimalLongitude: 106.70173; geodeticDatum: WGS84; georeferenceProtocol: GPS; **Identification:** identifiedBy: Do Van Tu; dateIdentified: 2020; **Event:** samplingProtocol: hand net; eventDate: 05/26/2017; year: 2017; month: 5; day: 26; **Record Level:** type: PhysicalObject; institutionCode: ZMB; collectionCode: ZMB; basisOfRecord: PreservedSpecimen
- **Type status:**Other material. **Occurrence:** catalogNumber: ZMB 30305; recordedBy: Do Van Tu; disposition: in collection; **Taxon:** scientificName: Neocaridinapalmatapalmata (Shen, 1948); kingdom: Animal; phylum: Arthropoda; class: Malacostraca; order: Decapoda; family: Atyidae; genus: Neocaridina; specificEpithet: palmata; infraspecificEpithet: palmata; taxonRank: Subspecies; scientificNameAuthorship: (Shen, 1948); vernacularName: atyid shrimp; nomenclaturalCode: ICZN; taxonomicStatus: accepted; **Location:** country: Vietnam; countryCode: VN; stateProvince: Cao Bang; county: Trung Khanh; locality: Ban Gioc waterfall, Trung Khanh; decimalLatitude: 22.85298; decimalLongitude: 106.72369; geodeticDatum: WGS84; georeferenceProtocol: GPS; **Identification:** identifiedBy: Do Van Tu; dateIdentified: 2020; **Event:** samplingProtocol: hand net; eventDate: 05/26/2017; year: 2017; month: 5; day: 26; **Record Level:** type: PhysicalObject; institutionCode: ZMB; collectionCode: ZMB; basisOfRecord: PreservedSpecimen
- **Type status:**Other material. **Occurrence:** catalogNumber: ZMB 30315; recordedBy: Do Van Tu; disposition: in collection; **Taxon:** scientificName: Neocaridinapalmatapalmata (Shen, 1948); kingdom: Animal; phylum: Arthropoda; class: Malacostraca; order: Decapoda; family: Atyidae; genus: Neocaridina; specificEpithet: palmata; infraspecificEpithet: palmata; taxonRank: Subspecies; scientificNameAuthorship: (Shen, 1948); vernacularName: atyid shrimp; nomenclaturalCode: ICZN; taxonomicStatus: accepted; **Location:** country: Vietnam; countryCode: VN; stateProvince: Cao Bang; county: Ha Quang; municipality: Lung Nam; locality: Stream near the road to Thin Tang village; decimalLatitude: 22.97640; decimalLongitude: 106.05789; geodeticDatum: WGS84; georeferenceProtocol: GPS; **Identification:** identifiedBy: Do Van Tu; dateIdentified: 2020; **Event:** samplingProtocol: hand net; eventDate: 05/25/2017; year: 2017; month: 5; day: 25; **Record Level:** type: PhysicalObject; institutionCode: ZMB; collectionCode: ZMB; basisOfRecord: PreservedSpecimen
- **Type status:**Other material. **Occurrence:** catalogNumber: ZMB 30344; recordedBy: Do Van Tu; disposition: in collection; **Taxon:** scientificName: Neocaridinapalmatapalmata (Shen, 1948); kingdom: Animal; phylum: Arthropoda; class: Malacostraca; order: Decapoda; family: Atyidae; genus: Neocaridina; specificEpithet: palmata; infraspecificEpithet: palmata; taxonRank: Subspecies; scientificNameAuthorship: (Shen, 1948); vernacularName: atyid shrimp; nomenclaturalCode: ICZN; taxonomicStatus: accepted; **Location:** country: Vietnam; countryCode: VN; stateProvince: Cao Bang; county: Quang Yen; municipality: Tu Do; locality: Ban Moi village, 5 m from cave entrance; decimalLatitude: 22.66360; decimalLongitude: 106.39370; geodeticDatum: WGS84; georeferenceProtocol: GPS; **Identification:** identifiedBy: Do Van Tu; dateIdentified: 2020; **Event:** samplingProtocol: hand net; eventDate: 06/04/2019; year: 2019; month: 6; day: 4; **Record Level:** type: PhysicalObject; institutionCode: ZMB; collectionCode: ZMB; basisOfRecord: PreservedSpecimen
- **Type status:**Other material. **Occurrence:** catalogNumber: ZMB 30347; recordedBy: Do Van Tu; disposition: in collection; **Taxon:** scientificName: Neocaridinapalmatapalmata (Shen, 1948); kingdom: Animal; phylum: Arthropoda; class: Malacostraca; order: Decapoda; family: Atyidae; genus: Neocaridina; specificEpithet: palmata; infraspecificEpithet: palmata; taxonRank: Subspecies; scientificNameAuthorship: (Shen, 1948); vernacularName: atyid shrimp; nomenclaturalCode: ICZN; taxonomicStatus: accepted; **Location:** country: Vietnam; countryCode: VN; stateProvince: Cao Bang; county: Phuc Hoa; municipality: Luong Thien; locality: Lung Co, mouth of cave; decimalLatitude: 22.57913; decimalLongitude: 106.48076; geodeticDatum: WGS84; georeferenceProtocol: GPS; **Identification:** identifiedBy: Do Van Tu; dateIdentified: 2020; **Event:** samplingProtocol: hand net; eventDate: 06/03/2019; year: 2019; month: 6; day: 3; **Record Level:** type: PhysicalObject; institutionCode: ZMB; collectionCode: ZMB; basisOfRecord: PreservedSpecimen
- **Type status:**Other material. **Occurrence:** catalogNumber: ZMB 30348; recordedBy: Do Van Tu; disposition: in collection; **Taxon:** scientificName: Neocaridinapalmatapalmata (Shen, 1948); kingdom: Animal; phylum: Arthropoda; class: Malacostraca; order: Decapoda; family: Atyidae; genus: Neocaridina; specificEpithet: palmata; infraspecificEpithet: palmata; taxonRank: Subspecies; scientificNameAuthorship: (Shen, 1948); vernacularName: atyid shrimp; nomenclaturalCode: ICZN; taxonomicStatus: accepted; **Location:** country: Vietnam; countryCode: VN; stateProvince: Bac Kan; county: Ba Be; municipality: Cao Thuong; decimalLatitude: 22.46747; decimalLongitude: 105.63759; geodeticDatum: WGS84; georeferenceProtocol: GPS; **Identification:** identifiedBy: Do Van Tu; dateIdentified: 2020; **Event:** samplingProtocol: hand net; eventDate: 06/05/2019; year: 2019; month: 6; day: 5; **Record Level:** type: PhysicalObject; institutionCode: ZMB; collectionCode: ZMB; basisOfRecord: PreservedSpecimen
- **Type status:**Other material. **Occurrence:** catalogNumber: ZMB 30349; recordedBy: Do Van Tu; disposition: in collection; **Taxon:** scientificName: Neocaridinapalmatapalmata (Shen, 1948); kingdom: Animal; phylum: Arthropoda; class: Malacostraca; order: Decapoda; family: Atyidae; genus: Neocaridina; specificEpithet: palmata; infraspecificEpithet: palmata; taxonRank: Subspecies; scientificNameAuthorship: (Shen, 1948); vernacularName: atyid shrimp; nomenclaturalCode: ICZN; taxonomicStatus: accepted; **Location:** country: Vietnam; countryCode: VN; stateProvince: Cao Bang; county: Quang Yen; municipality: Tu Do; locality: Ban Moi village, 5 m from cave entrance; decimalLatitude: 22.66360; decimalLongitude: 106.39370; geodeticDatum: WGS84; georeferenceProtocol: GPS; **Identification:** identifiedBy: Do Van Tu; dateIdentified: 2020; **Event:** samplingProtocol: hand net; eventDate: 06/04/2019; year: 2019; month: 6; day: 4; **Record Level:** type: PhysicalObject; institutionCode: ZMB; collectionCode: ZMB; basisOfRecord: PreservedSpecimen
- **Type status:**Other material. **Occurrence:** catalogNumber: ZMB 30352; recordedBy: Do Van Tu; disposition: in collection; **Taxon:** scientificName: Neocaridinapalmatapalmata (Shen, 1948); kingdom: Animal; phylum: Arthropoda; class: Malacostraca; order: Decapoda; family: Atyidae; genus: Neocaridina; specificEpithet: palmata; infraspecificEpithet: palmata; taxonRank: Subspecies; scientificNameAuthorship: (Shen, 1948); vernacularName: atyid shrimp; nomenclaturalCode: ICZN; taxonomicStatus: accepted; **Location:** country: Vietnam; countryCode: VN; stateProvince: Lang Son; county: Huu Lung; municipality: Yen Thinh; decimalLatitude: 21.62204; decimalLongitude: 106.33551; geodeticDatum: WGS84; georeferenceProtocol: GPS; **Identification:** identifiedBy: Do Van Tu; dateIdentified: 2020; **Event:** samplingProtocol: hand net; eventDate: 06/08/2019; year: 2019; month: 6; day: 8; **Record Level:** type: PhysicalObject; institutionCode: ZMB; collectionCode: ZMB; basisOfRecord: PreservedSpecimen
- **Type status:**Other material. **Occurrence:** catalogNumber: ZMB 30359; recordedBy: Do Van Tu, Dang Van Dong, Le Hung Anh; disposition: in collection; **Taxon:** scientificName: Neocaridinapalmatapalmata (Shen, 1948); kingdom: Animal; phylum: Arthropoda; class: Malacostraca; order: Decapoda; family: Atyidae; genus: Neocaridina; specificEpithet: palmata; infraspecificEpithet: palmata; taxonRank: Subspecies; scientificNameAuthorship: (Shen, 1948); vernacularName: atyid shrimp; nomenclaturalCode: ICZN; taxonomicStatus: accepted; **Location:** country: Vietnam; countryCode: VN; stateProvince: Ha Giang; county: Bac Me; municipality: Minh Ngoc; locality: Stream in Lung Cang village (LC1); decimalLatitude: 22.71736; decimalLongitude: 105.18694; geodeticDatum: WGS84; georeferenceProtocol: GPS; **Identification:** identifiedBy: Do Van Tu; dateIdentified: 2020; **Event:** samplingProtocol: hand net; eventDate: 10/17/2019; year: 2019; month: 10; day: 17; **Record Level:** type: PhysicalObject; institutionCode: ZMB; collectionCode: ZMB; basisOfRecord: PreservedSpecimen
- **Type status:**Other material. **Occurrence:** catalogNumber: ZMB 30361; recordedBy: Do Van Tu, Dang Van Dong; disposition: in collection; **Taxon:** scientificName: Neocaridinapalmatapalmata (Shen, 1948); kingdom: Animal; phylum: Arthropoda; class: Malacostraca; order: Decapoda; family: Atyidae; genus: Neocaridina; specificEpithet: palmata; infraspecificEpithet: palmata; taxonRank: Subspecies; scientificNameAuthorship: (Shen, 1948); vernacularName: atyid shrimp; nomenclaturalCode: ICZN; taxonomicStatus: accepted; **Location:** country: Vietnam; countryCode: VN; stateProvince: Ha Giang; county: Bac Me; municipality: Lac Nong; locality: Bac Me, small stream (LN1); decimalLatitude: 22.73851; decimalLongitude: 105.26039; geodeticDatum: WGS84; georeferenceProtocol: GPS; **Identification:** identifiedBy: Do Van Tu; dateIdentified: 2020; **Event:** samplingProtocol: hand net; eventDate: 10/16/2019; year: 2019; month: 10; day: 16; **Record Level:** type: PhysicalObject; institutionCode: ZMB; collectionCode: ZMB; basisOfRecord: PreservedSpecimen
- **Type status:**Other material. **Occurrence:** catalogNumber: ZMB 30362; recordedBy: Do Van Tu, Dang Van Dong; disposition: in collection; **Taxon:** scientificName: Neocaridinapalmatapalmata (Shen, 1948); kingdom: Animal; phylum: Arthropoda; class: Malacostraca; order: Decapoda; family: Atyidae; genus: Neocaridina; specificEpithet: palmata; infraspecificEpithet: palmata; taxonRank: Subspecies; scientificNameAuthorship: (Shen, 1948); vernacularName: atyid shrimp; nomenclaturalCode: ICZN; taxonomicStatus: accepted; **Location:** country: Vietnam; countryCode: VN; stateProvince: Ha Giang; county: Bac Me; municipality: Lac Nong; locality: Very small stream (LN2); decimalLatitude: 22.74025; decimalLongitude: 105.23611; geodeticDatum: WGS84; georeferenceProtocol: GPS; **Identification:** identifiedBy: Do Van Tu; dateIdentified: 2020; **Event:** samplingProtocol: hand net; eventDate: 10/16/2019; year: 2019; month: 10; day: 16; **Record Level:** type: PhysicalObject; institutionCode: ZMB; collectionCode: ZMB; basisOfRecord: PreservedSpecimen
- **Type status:**Other material. **Occurrence:** catalogNumber: ZMB 30364; recordedBy: Do Van Tu, Dang Van Dong, Le Hung Anh; disposition: in collection; **Taxon:** scientificName: Neocaridinapalmatapalmata (Shen, 1948); kingdom: Animal; phylum: Arthropoda; class: Malacostraca; order: Decapoda; family: Atyidae; genus: Neocaridina; specificEpithet: palmata; infraspecificEpithet: palmata; taxonRank: Subspecies; scientificNameAuthorship: (Shen, 1948); vernacularName: atyid shrimp; nomenclaturalCode: ICZN; taxonomicStatus: accepted; **Location:** country: Vietnam; countryCode: VN; stateProvince: Ha Giang; county: Bac Me; municipality: Lac Nong; locality: Stream in Khen village; decimalLatitude: 22.75570; decimalLongitude: 105.24593; geodeticDatum: WGS84; georeferenceProtocol: GPS; **Identification:** identifiedBy: Do Van Tu; dateIdentified: 2020; **Event:** samplingProtocol: hand net; eventDate: 10/18/2019; year: 2019; month: 10; day: 18; **Record Level:** type: PhysicalObject; institutionCode: ZMB; collectionCode: ZMB; basisOfRecord: PreservedSpecimen
- **Type status:**Other material. **Occurrence:** catalogNumber: ZMB 30382; recordedBy: Dang Van Dong; disposition: in collection; **Taxon:** scientificName: Neocaridinapalmatapalmata (Shen, 1948); kingdom: Animal; phylum: Arthropoda; class: Malacostraca; order: Decapoda; family: Atyidae; genus: Neocaridina; specificEpithet: palmata; infraspecificEpithet: palmata; taxonRank: Subspecies; scientificNameAuthorship: (Shen, 1948); vernacularName: atyid shrimp; nomenclaturalCode: ICZN; taxonomicStatus: accepted; **Location:** country: Vietnam; countryCode: VN; stateProvince: Cao Bang; county: Thach An; municipality: Kim Dong; decimalLatitude: 22.56444; decimalLongitude: 106.31917; geodeticDatum: WGS84; georeferenceProtocol: GPS; **Identification:** identifiedBy: Do Van Tu; dateIdentified: 2020; **Event:** samplingProtocol: hand net; eventDate: 04/28/2019; year: 2019; month: 4; day: 28; **Record Level:** type: PhysicalObject; institutionCode: ZMB; collectionCode: ZMB; basisOfRecord: PreservedSpecimen
- **Type status:**Other material. **Occurrence:** catalogNumber: ZMB 30384; recordedBy: Dang Van Dong; disposition: in collection; **Taxon:** scientificName: Neocaridinapalmatapalmata (Shen, 1948); kingdom: Animal; phylum: Arthropoda; class: Malacostraca; order: Decapoda; family: Atyidae; genus: Neocaridina; specificEpithet: palmata; infraspecificEpithet: palmata; taxonRank: Subspecies; scientificNameAuthorship: (Shen, 1948); vernacularName: atyid shrimp; nomenclaturalCode: ICZN; taxonomicStatus: accepted; **Location:** country: Vietnam; countryCode: VN; stateProvince: Cao Bang; county: Hoa An; decimalLatitude: 22.69306; decimalLongitude: 106.20361; geodeticDatum: WGS84; georeferenceProtocol: GPS; **Identification:** identifiedBy: Do Van Tu; dateIdentified: 2020; **Event:** samplingProtocol: hand net; eventDate: 04/29/2019; year: 2019; month: 4; day: 29; **Record Level:** type: PhysicalObject; institutionCode: ZMB; collectionCode: ZMB; basisOfRecord: PreservedSpecimen
- **Type status:**Other material. **Occurrence:** catalogNumber: ZMB 30661; recordedBy: Do Van Tu; disposition: in collection; **Taxon:** scientificName: Neocaridinapalmatapalmata (Shen, 1948); kingdom: Animal; phylum: Arthropoda; class: Malacostraca; order: Decapoda; family: Atyidae; genus: Neocaridina; specificEpithet: palmata; infraspecificEpithet: palmata; taxonRank: Subspecies; scientificNameAuthorship: (Shen, 1948); vernacularName: atyid shrimp; nomenclaturalCode: ICZN; taxonomicStatus: accepted; **Location:** country: Vietnam; countryCode: VN; stateProvince: Yen Bai; county: Van Chan; decimalLatitude: 21.47856; decimalLongitude: 104.73222; geodeticDatum: WGS84; georeferenceProtocol: GPS; **Identification:** identifiedBy: Do Van Tu; dateIdentified: 2020; **Event:** samplingProtocol: hand net; eventDate: 11/20/2012; year: 2012; month: 11; day: 20; **Record Level:** type: PhysicalObject; institutionCode: ZMB; collectionCode: ZMB; basisOfRecord: PreservedSpecimen
- **Type status:**Other material. **Occurrence:** catalogNumber: ZMB 30728; recordedBy: Tran Anh Duc; disposition: in collection; **Taxon:** scientificName: Neocaridinapalmatapalmata (Shen, 1948); kingdom: Animal; phylum: Arthropoda; class: Malacostraca; order: Decapoda; family: Atyidae; genus: Neocaridina; specificEpithet: palmata; infraspecificEpithet: palmata; taxonRank: Subspecies; scientificNameAuthorship: (Shen, 1948); vernacularName: atyid shrimp; nomenclaturalCode: ICZN; taxonomicStatus: accepted; **Location:** country: Vietnam; countryCode: VN; stateProvince: Bac Kan; county: Na Ri; municipality: Cu Le; locality: Stream by the road; decimalLatitude: 22.1275; decimalLongitude: 106.14972; geodeticDatum: WGS84; georeferenceProtocol: GPS; **Identification:** identifiedBy: Do Van Tu; dateIdentified: 2020; **Event:** samplingProtocol: hand net; eventDate: 08/23/2012; year: 2012; month: 8; day: 23; **Record Level:** type: PhysicalObject; institutionCode: ZMB; collectionCode: ZMB; basisOfRecord: PreservedSpecimen
- **Type status:**Other material. **Occurrence:** catalogNumber: ZMB 31566; recordedBy: Do Van Tu; disposition: in collection; **Taxon:** scientificName: Neocaridinapalmatapalmata (Shen, 1948); kingdom: Animal; phylum: Arthropoda; class: Malacostraca; order: Decapoda; family: Atyidae; genus: Neocaridina; specificEpithet: palmata; infraspecificEpithet: palmata; taxonRank: Subspecies; scientificNameAuthorship: (Shen, 1948); vernacularName: atyid shrimp; nomenclaturalCode: ICZN; taxonomicStatus: accepted; **Location:** country: Vietnam; countryCode: VN; stateProvince: Cao Bang; county: Quang Yen; municipality: Hanh Phuc; locality: Stream in Thom Dan; decimalLatitude: 22.57448; decimalLongitude: 106.44124; geodeticDatum: WGS84; georeferenceProtocol: GPS; **Identification:** identifiedBy: Do Van Tu; dateIdentified: 2020; **Event:** samplingProtocol: hand net; eventDate: 05/27/2017; year: 2017; month: 5; day: 27; **Record Level:** type: PhysicalObject; institutionCode: ZMB; collectionCode: ZMB; basisOfRecord: PreservedSpecimen
- **Type status:**Other material. **Occurrence:** catalogNumber: ZMB 31568; recordedBy: Do Van Tu; disposition: in collection; **Taxon:** scientificName: Neocaridinapalmatapalmata (Shen, 1948); kingdom: Animal; phylum: Arthropoda; class: Malacostraca; order: Decapoda; family: Atyidae; genus: Neocaridina; specificEpithet: palmata; infraspecificEpithet: palmata; taxonRank: Subspecies; scientificNameAuthorship: (Shen, 1948); vernacularName: atyid shrimp; nomenclaturalCode: ICZN; taxonomicStatus: accepted; **Location:** country: Vietnam; countryCode: VN; stateProvince: Cao Bang; county: Phuc Hoa; municipality: My Hung; locality: Stream from Nguom Khuoi Khua Cave at mouth of cave, Na Rieng; decimalLatitude: 22.48633; decimalLongitude: 106.55091; geodeticDatum: WGS84; georeferenceProtocol: GPS; **Identification:** identifiedBy: Do Van Tu; dateIdentified: 2020; **Event:** samplingProtocol: hand net; eventDate: 05/27/2017; year: 2017; month: 5; day: 27; **Record Level:** type: PhysicalObject; institutionCode: ZMB; collectionCode: ZMB; basisOfRecord: PreservedSpecimen
- **Type status:**Other material. **Occurrence:** catalogNumber: ZMB 31572; recordedBy: Do Van Tu; disposition: in collection; **Taxon:** scientificName: Neocaridinapalmatapalmata (Shen, 1948); kingdom: Animal; phylum: Arthropoda; class: Malacostraca; order: Decapoda; family: Atyidae; genus: Neocaridina; specificEpithet: palmata; infraspecificEpithet: palmata; taxonRank: Subspecies; scientificNameAuthorship: (Shen, 1948); vernacularName: atyid shrimp; nomenclaturalCode: ICZN; taxonomicStatus: accepted; **Location:** country: Vietnam; countryCode: VN; stateProvince: Cao Bang; county: Quang Yen; municipality: Hanh Phuc; locality: Stream in Hanh Phuc, Quang Uyen; decimalLatitude: 22.60187; decimalLongitude: 106.43682; geodeticDatum: WGS84; georeferenceProtocol: GPS; **Identification:** identifiedBy: Do Van Tu; dateIdentified: 2020; **Event:** samplingProtocol: hand net; eventDate: 05/27/2017; year: 2017; month: 5; day: 27; **Record Level:** type: PhysicalObject; institutionCode: ZMB; collectionCode: ZMB; basisOfRecord: PreservedSpecimen
- **Type status:**Other material. **Occurrence:** catalogNumber: ZMB 31579; recordedBy: Do Van Tu; disposition: in collection; **Taxon:** scientificName: Neocaridinapalmatapalmata (Shen, 1948); kingdom: Animal; phylum: Arthropoda; class: Malacostraca; order: Decapoda; family: Atyidae; genus: Neocaridina; specificEpithet: palmata; infraspecificEpithet: palmata; taxonRank: Subspecies; scientificNameAuthorship: (Shen, 1948); vernacularName: atyid shrimp; nomenclaturalCode: ICZN; taxonomicStatus: accepted; **Location:** country: Vietnam; countryCode: VN; stateProvince: Lang Son; decimalLatitude: 21.98199; decimalLongitude: 106.67419997222; geodeticDatum: WGS84; georeferenceProtocol: GPS; **Identification:** identifiedBy: Do Van Tu; dateIdentified: 2020; **Event:** samplingProtocol: hand net; eventDate: 03/11/2018; year: 2018; month: 3; day: 11; **Record Level:** type: PhysicalObject; institutionCode: ZMB; collectionCode: ZMB; basisOfRecord: PreservedSpecimen
- **Type status:**Other material. **Occurrence:** catalogNumber: ZMB 31590; recordedBy: Do Van Tu; disposition: in collection; **Taxon:** scientificName: Neocaridinapalmatapalmata (Shen, 1948); kingdom: Animal; phylum: Arthropoda; class: Malacostraca; order: Decapoda; family: Atyidae; genus: Neocaridina; specificEpithet: palmata; infraspecificEpithet: palmata; taxonRank: Subspecies; scientificNameAuthorship: (Shen, 1948); vernacularName: atyid shrimp; nomenclaturalCode: ICZN; taxonomicStatus: accepted; **Location:** country: Vietnam; countryCode: VN; stateProvince: Cao Bang; county: Ha Lang; municipality: Duc Quang; locality: Coong village; decimalLatitude: 22.71833; decimalLongitude: 106.65583; geodeticDatum: WGS84; georeferenceProtocol: GPS; **Identification:** identifiedBy: Do Van Tu; dateIdentified: 2020; **Event:** samplingProtocol: hand net; eventDate: 07/28/2018; year: 2018; month: 7; day: 28; **Record Level:** type: PhysicalObject; institutionCode: ZMB; collectionCode: ZMB; basisOfRecord: PreservedSpecimen
- **Type status:**Other material. **Occurrence:** catalogNumber: ZMB 31591; recordedBy: Do Van Tu; disposition: in collection; **Taxon:** scientificName: Neocaridinapalmatapalmata (Shen, 1948); kingdom: Animal; phylum: Arthropoda; class: Malacostraca; order: Decapoda; family: Atyidae; genus: Neocaridina; specificEpithet: palmata; infraspecificEpithet: palmata; taxonRank: Subspecies; scientificNameAuthorship: (Shen, 1948); vernacularName: atyid shrimp; nomenclaturalCode: ICZN; taxonomicStatus: accepted; **Location:** country: Vietnam; countryCode: VN; stateProvince: Lang Son; decimalLatitude: 21.99478; decimalLongitude: 106.65314; geodeticDatum: WGS84; georeferenceProtocol: GPS; **Identification:** identifiedBy: Do Van Tu; dateIdentified: 2020; **Event:** samplingProtocol: hand net; eventDate: 03/11/2018; year: 2018; month: 3; day: 11; **Record Level:** type: PhysicalObject; institutionCode: ZMB; collectionCode: ZMB; basisOfRecord: PreservedSpecimen
- **Type status:**Other material. **Occurrence:** catalogNumber: ZMB 31593; recordedBy: Do Van Tu; disposition: in collection; **Taxon:** scientificName: Neocaridinapalmatapalmata (Shen, 1948); kingdom: Animal; phylum: Arthropoda; class: Malacostraca; order: Decapoda; family: Atyidae; genus: Neocaridina; specificEpithet: palmata; infraspecificEpithet: palmata; taxonRank: Subspecies; scientificNameAuthorship: (Shen, 1948); vernacularName: atyid shrimp; nomenclaturalCode: ICZN; taxonomicStatus: accepted; **Location:** country: Vietnam; countryCode: VN; stateProvince: Cao Bang; county: Ha Lang; municipality: Kim Loan; locality: Tung Kit village; decimalLatitude: 22.73806; decimalLongitude: 106.60222; geodeticDatum: WGS84; georeferenceProtocol: GPS; **Identification:** identifiedBy: Do Van Tu; dateIdentified: 2020; **Event:** samplingProtocol: hand net; eventDate: 07/31/2018; year: 2018; month: 7; day: 31; **Record Level:** type: PhysicalObject; institutionCode: ZMB; collectionCode: ZMB; basisOfRecord: PreservedSpecimen
- **Type status:**Other material. **Occurrence:** catalogNumber: ZMB 31596; recordedBy: Do Van Tu; disposition: in collection; **Taxon:** scientificName: Neocaridinapalmatapalmata (Shen, 1948); kingdom: Animal; phylum: Arthropoda; class: Malacostraca; order: Decapoda; family: Atyidae; genus: Neocaridina; specificEpithet: palmata; infraspecificEpithet: palmata; taxonRank: Subspecies; scientificNameAuthorship: (Shen, 1948); vernacularName: atyid shrimp; nomenclaturalCode: ICZN; taxonomicStatus: accepted; **Location:** country: Vietnam; countryCode: VN; stateProvince: Cao Bang; county: Quang Yen; municipality: Phuc Sen; decimalLatitude: 22.681; decimalLongitude: 106.41139; geodeticDatum: WGS84; georeferenceProtocol: GPS; **Identification:** identifiedBy: Do Van Tu; dateIdentified: 2020; **Event:** samplingProtocol: hand net; eventDate: 06/27/2018; year: 2018; month: 6; day: 27; **Record Level:** type: PhysicalObject; institutionCode: ZMB; collectionCode: ZMB; basisOfRecord: PreservedSpecimen
- **Type status:**Other material. **Occurrence:** catalogNumber: ZMB 31599; recordedBy: Do Van Tu; disposition: in collection; **Taxon:** scientificName: Neocaridinapalmatapalmata (Shen, 1948); kingdom: Animal; phylum: Arthropoda; class: Malacostraca; order: Decapoda; family: Atyidae; genus: Neocaridina; specificEpithet: palmata; infraspecificEpithet: palmata; taxonRank: Subspecies; scientificNameAuthorship: (Shen, 1948); vernacularName: atyid shrimp; nomenclaturalCode: ICZN; taxonomicStatus: accepted; **Location:** country: Vietnam; countryCode: VN; stateProvince: Bac Kan; county: Ngan Son; locality: Stream behind Ban Dam lake, Ban Chang; decimalLatitude: 22.45528; decimalLongitude: 106.02925; geodeticDatum: WGS84; georeferenceProtocol: GPS; **Identification:** identifiedBy: Do Van Tu; dateIdentified: 2020; **Event:** samplingProtocol: hand net; eventDate: 03/17/2018; year: 2018; month: 3; day: 17; **Record Level:** type: PhysicalObject; institutionCode: ZMB; collectionCode: ZMB; basisOfRecord: PreservedSpecimen
- **Type status:**Other material. **Occurrence:** catalogNumber: ZMB 31607; recordedBy: Do Van Tu; disposition: in collection; **Taxon:** scientificName: Neocaridinapalmatapalmata (Shen, 1948); kingdom: Animal; phylum: Arthropoda; class: Malacostraca; order: Decapoda; family: Atyidae; genus: Neocaridina; specificEpithet: palmata; infraspecificEpithet: palmata; taxonRank: Subspecies; scientificNameAuthorship: (Shen, 1948); vernacularName: atyid shrimp; nomenclaturalCode: ICZN; taxonomicStatus: accepted; **Location:** country: Vietnam; countryCode: VN; stateProvince: Lang Son; decimalLatitude: 21.98199; decimalLongitude: 106.67420; geodeticDatum: WGS84; georeferenceProtocol: GPS; **Identification:** identifiedBy: Do Van Tu; dateIdentified: 2020; **Event:** samplingProtocol: hand net; eventDate: 03/11/2018; year: 2018; month: 3; day: 11; **Record Level:** type: PhysicalObject; institutionCode: ZMB; collectionCode: ZMB; basisOfRecord: PreservedSpecimen
- **Type status:**Other material. **Occurrence:** catalogNumber: ZMB 30312; recordedBy: Do Van Tu; disposition: in collection; **Taxon:** scientificName: Neocaridinapalmatapalmata (Shen, 1948); kingdom: Animal; phylum: Arthropoda; class: Malacostraca; order: Decapoda; family: Atyidae; genus: Neocaridina; specificEpithet: palmata; infraspecificEpithet: palmata; taxonRank: Subspecies; scientificNameAuthorship: (Shen, 1948); vernacularName: atyid shrimp; nomenclaturalCode: ICZN; taxonomicStatus: accepted; **Location:** country: Vietnam; countryCode: VN; stateProvince: Cao Bang; county: Ha Quang; municipality: Lung Nam; locality: Stream near the road to Thin Tang village; decimalLatitude: 22.97640; decimalLongitude: 106.05789; geodeticDatum: WGS84; georeferenceProtocol: GPS; **Identification:** identifiedBy: Do Van Tu; dateIdentified: 2020; **Event:** samplingProtocol: hand net; eventDate: 05/25/2017; year: 2017; month: 5; day: 25; **Record Level:** type: PhysicalObject; institutionCode: ZMB; collectionCode: ZMB; basisOfRecord: PreservedSpecimen
- **Type status:**Other material. **Occurrence:** catalogNumber: ZMB 33829; recordedBy: Do Van Tu; disposition: in collection; **Taxon:** scientificName: Neocaridinapalmatapalmata (Shen, 1948); kingdom: Animal; phylum: Arthropoda; class: Malacostraca; order: Decapoda; family: Atyidae; genus: Neocaridina; specificEpithet: palmata; infraspecificEpithet: palmata; taxonRank: Subspecies; scientificNameAuthorship: (Shen, 1948); vernacularName: atyid shrimp; nomenclaturalCode: ICZN; taxonomicStatus: accepted; **Location:** country: Vietnam; countryCode: VN; stateProvince: Ha Giang; county: Quan Ba; verbatimElevation: 827 m a.s.l.; decimalLatitude: 23.08654; decimalLongitude: 104.96605; geodeticDatum: WGS84; georeferenceProtocol: GPS; **Identification:** identifiedBy: Do Van Tu; dateIdentified: 2020; **Event:** samplingProtocol: hand net; eventDate: 10/13/2020; year: 2020; month: 10; day: 13; **Record Level:** type: PhysicalObject; institutionCode: ZMB; collectionCode: ZMB; basisOfRecord: PreservedSpecimen
- **Type status:**Other material. **Occurrence:** catalogNumber: ZMB 33779; recordedBy: Do Van Tu; disposition: in collection; **Taxon:** scientificName: Neocaridinapalmatapalmata (Shen, 1948); kingdom: Animal; phylum: Arthropoda; class: Malacostraca; order: Decapoda; family: Atyidae; genus: Neocaridina; specificEpithet: palmata; infraspecificEpithet: palmata; taxonRank: Subspecies; scientificNameAuthorship: (Shen, 1948); vernacularName: atyid shrimp; nomenclaturalCode: ICZN; taxonomicStatus: accepted; **Location:** country: Vietnam; countryCode: VN; stateProvince: Cao Bang; county: Nguyen Binh; decimalLatitude: 22.5925; decimalLongitude: 105.85472; geodeticDatum: WGS84; georeferenceProtocol: GPS; **Identification:** identifiedBy: Do Van Tu; dateIdentified: 2020; **Event:** samplingProtocol: hand net; eventDate: 05/21/2020; year: 2020; month: 5; day: 21; **Record Level:** type: PhysicalObject; institutionCode: ZMB; collectionCode: ZMB; basisOfRecord: PreservedSpecimen
- **Type status:**Other material. **Occurrence:** catalogNumber: ZMB 33830; recordedBy: Do Van Tu; disposition: in collection; **Taxon:** scientificName: Neocaridinapalmatapalmata (Shen, 1948); kingdom: Animal; phylum: Arthropoda; class: Malacostraca; order: Decapoda; family: Atyidae; genus: Neocaridina; specificEpithet: palmata; infraspecificEpithet: palmata; taxonRank: Subspecies; scientificNameAuthorship: (Shen, 1948); vernacularName: atyid shrimp; nomenclaturalCode: ICZN; taxonomicStatus: accepted; **Location:** country: Vietnam; countryCode: VN; stateProvince: Cao Bang; county: Nguyen Binh; decimalLatitude: 22.65306; decimalLongitude: 105.92278; geodeticDatum: WGS84; georeferenceProtocol: GPS; **Identification:** identifiedBy: Do Van Tu; dateIdentified: 2020; **Event:** samplingProtocol: hand net; eventDate: 05/22/2020; year: 2020; month: 5; day: 22; **Record Level:** type: PhysicalObject; institutionCode: ZMB; collectionCode: ZMB; basisOfRecord: PreservedSpecimen
- **Type status:**Other material. **Occurrence:** catalogNumber: ZMB 33780; recordedBy: Do Van Tu, Dang Van Dong, Nguyen Tong Cuong'; disposition: in collection; **Taxon:** scientificName: Neocaridinapalmatapalmata (Shen, 1948); kingdom: Animal; phylum: Arthropoda; class: Malacostraca; order: Decapoda; family: Atyidae; genus: Neocaridina; specificEpithet: palmata; infraspecificEpithet: palmata; taxonRank: Subspecies; scientificNameAuthorship: (Shen, 1948); vernacularName: atyid shrimp; nomenclaturalCode: ICZN; taxonomicStatus: accepted; **Location:** country: Vietnam; countryCode: VN; stateProvince: Bac Kan; county: Cho Don; municipality: Xuan Lac; verbatimElevation: 760 m a.s.l.; decimalLatitude: 22.29314; decimalLongitude: 105.51742; geodeticDatum: WGS84; georeferenceProtocol: GPS; **Identification:** identifiedBy: Do Van Tu; dateIdentified: 2020; **Event:** samplingProtocol: hand net; eventDate: 07/24/2020; year: 2020; month: 7; day: 24; **Record Level:** type: PhysicalObject; institutionCode: ZMB; collectionCode: ZMB; basisOfRecord: PreservedSpecimen
- **Type status:**Other material. **Occurrence:** catalogNumber: ZMB 33781; recordedBy: Do Van Tu, Dang Van Dong, Nguyen Tong Cuong'; disposition: in collection; **Taxon:** scientificName: Neocaridinapalmatapalmata (Shen, 1948); kingdom: Animal; phylum: Arthropoda; class: Malacostraca; order: Decapoda; family: Atyidae; genus: Neocaridina; specificEpithet: palmata; infraspecificEpithet: palmata; taxonRank: Subspecies; scientificNameAuthorship: (Shen, 1948); vernacularName: atyid shrimp; nomenclaturalCode: ICZN; taxonomicStatus: accepted; **Location:** country: Vietnam; countryCode: VN; stateProvince: Bac Kan; county: Cho Don; verbatimElevation: 760 m a.s.l.; decimalLatitude: 22.29406; decimalLongitude: 105.51778; geodeticDatum: WGS84; georeferenceProtocol: GPS; **Identification:** identifiedBy: Do Van Tu; dateIdentified: 2020; **Event:** samplingProtocol: hand net; eventDate: 07/24/2020; year: 2020; month: 7; day: 24; **Record Level:** type: PhysicalObject; institutionCode: ZMB; collectionCode: ZMB; basisOfRecord: PreservedSpecimen
- **Type status:**Other material. **Occurrence:** catalogNumber: ZMB 33782; recordedBy: Do Van Tu, Dang Van Dong, Nguyen Tong Cuong'; disposition: in collection; **Taxon:** scientificName: Neocaridinapalmatapalmata (Shen, 1948); kingdom: Animal; phylum: Arthropoda; class: Malacostraca; order: Decapoda; family: Atyidae; genus: Neocaridina; specificEpithet: palmata; infraspecificEpithet: palmata; taxonRank: Subspecies; scientificNameAuthorship: (Shen, 1948); vernacularName: atyid shrimp; nomenclaturalCode: ICZN; taxonomicStatus: accepted; **Location:** country: Vietnam; countryCode: VN; stateProvince: Bac Kan; county: Cho Don; verbatimElevation: 342 m a.s.l.; decimalLatitude: 22.23546; decimalLongitude: 105.51070; geodeticDatum: WGS84; georeferenceProtocol: GPS; **Identification:** identifiedBy: Do Van Tu; dateIdentified: 2020; **Event:** samplingProtocol: hand net; eventDate: 07/25/2020; year: 2020; month: 7; day: 25; **Record Level:** type: PhysicalObject; institutionCode: ZMB; collectionCode: ZMB; basisOfRecord: PreservedSpecimen
- **Type status:**Other material. **Occurrence:** catalogNumber: ZMB 33783; recordedBy: Do Van Tu, Dang Van Dong, Nguyen Tong Cuong'; disposition: in collection; **Taxon:** scientificName: Neocaridinapalmatapalmata (Shen, 1948); kingdom: Animal; phylum: Arthropoda; class: Malacostraca; order: Decapoda; family: Atyidae; genus: Neocaridina; specificEpithet: palmata; infraspecificEpithet: palmata; taxonRank: Subspecies; scientificNameAuthorship: (Shen, 1948); vernacularName: atyid shrimp; nomenclaturalCode: ICZN; taxonomicStatus: accepted; **Location:** country: Vietnam; countryCode: VN; stateProvince: Bac Kan; county: Cho Don; decimalLatitude: 22.25667; decimalLongitude: 105.48694; geodeticDatum: WGS84; georeferenceProtocol: GPS; **Identification:** identifiedBy: Do Van Tu; dateIdentified: 2020; **Event:** samplingProtocol: hand net; eventDate: 07/25/2020; year: 2020; month: 7; day: 25; **Record Level:** type: PhysicalObject; institutionCode: ZMB; collectionCode: ZMB; basisOfRecord: PreservedSpecimen
- **Type status:**Other material. **Occurrence:** catalogNumber: ZMB 33784; recordedBy: Do Van Tu, Dang Van Dong, Nguyen Tong Cuong'; disposition: in collection; **Taxon:** scientificName: Neocaridinapalmatapalmata (Shen, 1948); kingdom: Animal; phylum: Arthropoda; class: Malacostraca; order: Decapoda; family: Atyidae; genus: Neocaridina; specificEpithet: palmata; infraspecificEpithet: palmata; taxonRank: Subspecies; scientificNameAuthorship: (Shen, 1948); vernacularName: atyid shrimp; nomenclaturalCode: ICZN; taxonomicStatus: accepted; **Location:** country: Vietnam; countryCode: VN; stateProvince: Bac Kan; county: Cho Don; verbatimElevation: 277 m a.s.l.; decimalLatitude: 22.21619; decimalLongitude: 105.49067; geodeticDatum: WGS84; georeferenceProtocol: GPS; **Identification:** identifiedBy: Do Van Tu; dateIdentified: 2020; **Event:** samplingProtocol: hand net; eventDate: 07/26/2020; year: 2020; month: 7; day: 26; **Record Level:** type: PhysicalObject; institutionCode: ZMB; collectionCode: ZMB; basisOfRecord: PreservedSpecimen
- **Type status:**Other material. **Occurrence:** catalogNumber: ZMB 33785; recordedBy: Do Van Tu, Dang Van Dong, Nguyen Tong Cuong'; disposition: in collection; **Taxon:** scientificName: Neocaridinapalmatapalmata (Shen, 1948); kingdom: Animal; phylum: Arthropoda; class: Malacostraca; order: Decapoda; family: Atyidae; genus: Neocaridina; specificEpithet: palmata; infraspecificEpithet: palmata; taxonRank: Subspecies; scientificNameAuthorship: (Shen, 1948); vernacularName: atyid shrimp; nomenclaturalCode: ICZN; taxonomicStatus: accepted; **Location:** country: Vietnam; countryCode: VN; stateProvince: Bac Kan; county: Cho Don; verbatimElevation: 307 m a.s.l.; decimalLatitude: 22.34364; decimalLongitude: 105.55027; geodeticDatum: WGS84; georeferenceProtocol: GPS; **Identification:** identifiedBy: Do Van Tu; dateIdentified: 2020; **Event:** samplingProtocol: hand net; eventDate: 07/27/2020; year: 2020; month: 7; day: 27; **Record Level:** type: PhysicalObject; institutionCode: ZMB; collectionCode: ZMB; basisOfRecord: PreservedSpecimen
- **Type status:**Other material. **Occurrence:** catalogNumber: ZMB 33786; recordedBy: Do Van Tu, Dang Van Dong, Nguyen Tong Cuong'; disposition: in collection; **Taxon:** scientificName: Neocaridinapalmatapalmata (Shen, 1948); kingdom: Animal; phylum: Arthropoda; class: Malacostraca; order: Decapoda; family: Atyidae; genus: Neocaridina; specificEpithet: palmata; infraspecificEpithet: palmata; taxonRank: Subspecies; scientificNameAuthorship: (Shen, 1948); vernacularName: atyid shrimp; nomenclaturalCode: ICZN; taxonomicStatus: accepted; **Location:** country: Vietnam; countryCode: VN; stateProvince: Bac Kan; county: Cho Don; verbatimElevation: 380 m a.s.l.; decimalLatitude: 22.32851; decimalLongitude: 105.52499; geodeticDatum: WGS84; georeferenceProtocol: GPS; **Identification:** identifiedBy: Do Van Tu; dateIdentified: 2020; **Event:** samplingProtocol: hand net; eventDate: 07/28/2020; year: 2020; month: 7; day: 28; **Record Level:** type: PhysicalObject; institutionCode: ZMB; collectionCode: ZMB; basisOfRecord: PreservedSpecimen
- **Type status:**Other material. **Occurrence:** catalogNumber: ZMB 33787; recordedBy: Do Van Tu, Dang Van Dong, Nguyen Tong Cuong'; disposition: in collection; **Taxon:** scientificName: Neocaridinapalmatapalmata (Shen, 1948); kingdom: Animal; phylum: Arthropoda; class: Malacostraca; order: Decapoda; family: Atyidae; genus: Neocaridina; specificEpithet: palmata; infraspecificEpithet: palmata; taxonRank: Subspecies; scientificNameAuthorship: (Shen, 1948); vernacularName: atyid shrimp; nomenclaturalCode: ICZN; taxonomicStatus: accepted; **Location:** country: Vietnam; countryCode: VN; stateProvince: Bac Kan; county: Cho Don; verbatimElevation: 300 m a.s.l.; decimalLatitude: 22.29353; decimalLongitude: 105.55729; geodeticDatum: WGS84; georeferenceProtocol: GPS; **Identification:** identifiedBy: Do Van Tu; dateIdentified: 2020; **Event:** samplingProtocol: hand net; eventDate: 07/29/2020; year: 2020; month: 7; day: 29; **Record Level:** type: PhysicalObject; institutionCode: ZMB; collectionCode: ZMB; basisOfRecord: PreservedSpecimen
- **Type status:**Other material. **Occurrence:** catalogNumber: ZMB 33778; recordedBy: Do Van Tu, Dang Van Dong, Nguyen Tong Cuong'; disposition: in collection; **Taxon:** scientificName: Neocaridinapalmatapalmata (Shen, 1948); kingdom: Animal; phylum: Arthropoda; class: Malacostraca; order: Decapoda; family: Atyidae; genus: Neocaridina; specificEpithet: palmata; infraspecificEpithet: palmata; taxonRank: Subspecies; scientificNameAuthorship: (Shen, 1948); vernacularName: atyid shrimp; nomenclaturalCode: ICZN; taxonomicStatus: accepted; **Location:** country: Vietnam; countryCode: VN; stateProvince: Cao Bang; county: Nguyen Binh; decimalLatitude: 22.5925; decimalLongitude: 105.85472; geodeticDatum: WGS84; georeferenceProtocol: GPS; **Identification:** identifiedBy: Do Van Tu; dateIdentified: 2020; **Event:** samplingProtocol: hand net; eventDate: 10/06/2020; year: 2020; month: 10; day: 6; **Record Level:** type: PhysicalObject; institutionCode: ZMB; collectionCode: ZMB; basisOfRecord: PreservedSpecimen
- **Type status:**Other material. **Occurrence:** catalogNumber: OUMNH.ZC 2013-07-042; recordedBy: Werner Klotz; disposition: in collection; **Taxon:** scientificName: Neocaridinapalmatapalmata (Shen, 1948); kingdom: Animal; phylum: Arthropoda; class: Malacostraca; order: Decapoda; family: Atyidae; genus: Neocaridina; specificEpithet: palmata; infraspecificEpithet: palmata; taxonRank: Subspecies; scientificNameAuthorship: (Shen, 1948); vernacularName: atyid shrimp; nomenclaturalCode: ICZN; taxonomicStatus: accepted; **Location:** island: Hong Kong; country: China; countryCode: CN; stateProvince: ﻿Hong Kong; locality: Nam Chung, below weir of Ping Nam stream; small trickle with mud and leaves; decimalLatitude: 22.5144; decimalLongitude: 114.2073; geodeticDatum: WGS84; georeferenceProtocol: GPS; **Identification:** identifiedBy: Do Van Tu; dateIdentified: 2020; **Event:** samplingProtocol: hand net; eventDate: 01/01/2011; year: 2011; month: 1; day: 1; **Record Level:** type: PhysicalObject; institutionCode: ZMB; collectionCode: ZMB; basisOfRecord: PreservedSpecimen
- **Type status:**Other material. **Occurrence:** catalogNumber: ZMB 28040; recordedBy: Werner & Maria Klotz, Yixiong Cai; disposition: in collection; **Taxon:** scientificName: Neocaridinapalmatapalmata (Shen, 1948); kingdom: Animal; phylum: Arthropoda; class: Malacostraca; order: Decapoda; family: Atyidae; genus: Neocaridina; specificEpithet: palmata; infraspecificEpithet: palmata; taxonRank: Subspecies; scientificNameAuthorship: (Shen, 1948); vernacularName: atyid shrimp; nomenclaturalCode: ICZN; taxonomicStatus: accepted; **Location:** country: China; countryCode: CN; stateProvince: Guangdong; locality: Gao Zhou Shi; decimalLatitude: 22.28532; decimalLongitude: 111.25777; geodeticDatum: WGS84; georeferenceProtocol: GPS; **Identification:** identifiedBy: Do Van Tu; dateIdentified: 2020; **Event:** samplingProtocol: hand net; eventDate: 03/29/2012; year: 2012; month: 3; day: 29; **Record Level:** type: PhysicalObject; institutionCode: ZMB; collectionCode: ZMB; basisOfRecord: PreservedSpecimen
- **Type status:**Other material. **Occurrence:** catalogNumber: ZMB 29762; recordedBy: Marco Endruweit; disposition: in collection; **Taxon:** scientificName: Neocaridinapalmatapalmata (Shen, 1948); kingdom: Animal; phylum: Arthropoda; class: Malacostraca; order: Decapoda; family: Atyidae; genus: Neocaridina; specificEpithet: palmata; infraspecificEpithet: palmata; taxonRank: Subspecies; scientificNameAuthorship: (Shen, 1948); vernacularName: atyid shrimp; nomenclaturalCode: ICZN; taxonomicStatus: accepted; **Location:** country: China; countryCode: CN; stateProvince: Guangxi; county: Napo; locality: Road after Delong Xiang, stream left of road, along road, at km marker 18, Gâm River subbasin, Red River basin; decimalLatitude: 23.23307; decimalLongitude: 105.87193; geodeticDatum: WGS84; georeferenceProtocol: GPS; **Identification:** identifiedBy: Do Van Tu; dateIdentified: 2020; **Event:** samplingProtocol: hand net; eventDate: 01/04/2012; year: 2012; month: 1; day: 4; **Record Level:** type: PhysicalObject; institutionCode: ZMB; collectionCode: ZMB; basisOfRecord: PreservedSpecimen

#### Description

**Material examined for description**. 5 males, cl 3.3–3.6, 5 females, cl 5.0–7.0 (ZMB 30303), Vietnam: Cao Bang Province, Trung Khanh District, Dam Thuy Commune, Ban Gun-Khuoi Ky Village, 22°50'42.88''N 106°42'6.22''E, [near type locality of *Caridinadenticulatavietnamensis* Dang, 1967], coll. Do Van Tu, 36 May 2017.

**Cephalothorax and cephalic appendages.** Carapace length 3.3–7.0 mm. Rostrum straight, slightly narrow, normally reaching the end of third segment of antennular peduncle, sometime reaching beyond the end of antennular peduncle, 0.58–0.74 (median 0.63) times as long as carapace, rostral formula: 3–4+8–13/3–6 (n = 10), teeth normal (Fig. 10a). Suborbital angle acute, completely fused with antennal spine; pterygostomian margin rounded, armed with a small spine (Fig. 10a). Eyes well developed with globular cornea, anterior end reaching to 0.7 times length of basal segment of antennular peduncle (Fig. 10a). Antennular peduncle 0.5–0.77 (median 0.68) times as long as carapace; basal segment 1.64–1.78 (median 1.71) times as long as second segment, anterolateral angle reaching 0.3 times length of the second segment, second segment 1.13–1.83 (median 1.43) times as long as third segment (Fig. 10b). Stylocerite reaching to beyond middle (0.8 times length) of basal segment of antennular peduncle (Fig. 10b). Scaphocerite reaching beyond distal end of antennular peduncle, 2.92–3.33 (median 3.15) times as long as wide (Fig. 10c).

**Abdominal somites, telson and uropods.** Sixth abdominal somite 0.46–0.54 (median 0.5) times length of carapace, 1.52–2.09 (median 1.67) times as long as fifth abdominal somite, shorter than telson, 0.7–0.91 (median 0.9) times length of telson. Telson length 2.69–3.67 (median 3.0) times as long as proximal width, distal margin obtuse angle, terminating in a short median projection, with 5–6 pairs of dorsal spiniform setae and one pair of dorso-subdistal spiniform setae; distal end with 4–5 pairs of spiniform setae, lateral pair slightly shorter than intermediate pairs (Fig. 10d). Pre-anal carina high, with few setae, lacking tooth or spine. Uropodal diaeresis with 12–14 movable spiniform setae, outermost one shorter than lateral angle (Fig. 10e).

**Pereiopods.** Epipod present on first four pereiopods. First pereiopod short, robust, reaching to distal end of basal segment of antennular peduncle; chela 1.91–2.21 (median 2.0) times as long as wide, 1.24–1.40 (median 1.36) times length of carpus; tips of fingers rounded, with hook; dactylus slightly longer than palm, 1.0–1.09 (median 1.04) times as long as palm; carpus excavated anteriorly, 1.50–1.67 (median 1.54) times as long as wide, 0.75–0.91 (median 0.79) times length of merus; merus 2.13–2.86 (median 2.37) times as long as wide, longer than ischium (Fig. 11a). Second pereiopod long, slender, reaching to middle of second segment of antennular peduncle; chela 2.4–3.0 (median 2.55) times as long as wide, 0.83–0.86 (median 0.85) times length of carpus; tips of fingers rounded, without hook; dactylus 1.2–1.5 (median 1.36) times as long as palm; carpus 4.38–4.67 (median 4.0) times as long as wide, slightly longer than merus, 1.0–1.06 (median 1.04) times as long as merus; merus 4.15–4.86 (median 4.5) times as long as wide, longer than ischium; coxa with a curved spine in male specimens (Fig. 11b). Third pereiopod slender, reaching to middle of third segment of antennular peduncle, with 6–8 accessory spiniform setae on flexor margin, dactylus 2.75–2.83 (median 2.78) times as long as wide (terminal claws and spiniform setae on flexor margin included), terminating in two claws, propodus 8.0–9.33 (median 8.17) times as long as wide, 2.59–3.42 (median 3.36) times as long as dactylus; dactylus and distal portion of propodus expanded in male specimens, propodus more strongly curved in males than in females; carpus 3.14–4.0 (median 3.67) times as long as wide, 0.61–0.88 (median 0.62) times as long as propodus, 0.44–0.61 (median 0.46) times as long as merus; merus 4.17–6.78 (median 4.59) times as long as wide, bearing three strong, movable spiniform setae on posterior margin of outer surface, merus stouter in males than in females; ischium with one movable spiniform setae (Fig. 11c, d). Fifth pereiopod slender, reaching to end of basal segment of antennular peduncle, dactylus 3.9–4.89 (median 4.4) times as long as wide (terminal claw and spiniform setae on flexor margin included), terminating in one large claw, with 45–63 spiniform setae on flexor margin; propodus 9.17–9.4 (median 9.2) times as long as wide, 2.2–3.0 (median 2.4) times length of dactylus; carpus 3.57–4.17 (median 4.0) times as long as wide, 0.45–0.54 (median 0.51) times as long as propodus, 0.60–0.67 (median 0.66) times as long as merus; merus 4.22–5.25 (median 4.5) times as long as wide, bearing 3 strong, movable spiniform setae on posterior margin of outer surface (Fig. 11e).

Endopod of male first pleopod extending to 0.8 times length of exopod, expanded into pear shape, 1.42–1.67 (median 1.45) times as long as proximal width, with tiny spiniform setae on distal margin of dorsal surface; outer margin lined with about 5–6 short setae; appendix interna reduced as a small protrusion or short finger at base of inflated part, without an arc of shorter process at the base of inner border (Fig. 12a, b). Appendix masculina of male second pleopod stout, taper-shaped, reaching to proximal 0.6 times endopod length, 1.5 times as long as distal width, inner and distal surface covered with many long, stout spiniform setae; appendix interna at the middle of appendix masculina, extending about 0.9 times length of appendix masculina (Fig. 12c, d).

**Figure 13.**
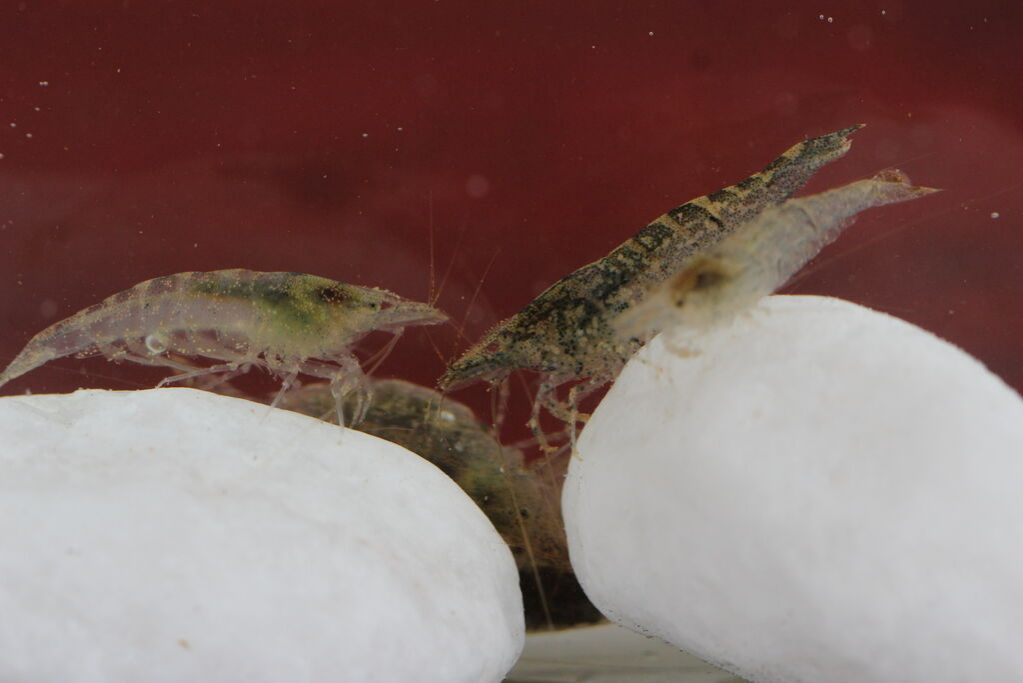
Live colouration of *Neocaridinapalmatapalmata* (Shen, 1948); collected from Phia Oac-Phia Den National Park, Cao Bang Province, details of the samples are given in the supplementary data.

**Colouration.** Body bluish or brown, few pale longitudinal stripes present on dorsal part of abdominal somites in large individuals (Fig. 13).

#### Distribution

In Vietnam, this species is distributed in the mountainous areas of the northeast and some parts of northwest Vietnam (Fig. 1d). In China, the species occurs in the following Provinces: Hubei, Jiangxi, Fujian, Guangdong, Guangxi, Guizhou, Anhui, Zhejiang (Liang 2004); and Hong Kong.

#### Ecology

Living in mountain streams and based on egg-size, this species can be considered a land-locked species (Table 1).

#### Taxon discussion

Dang (1967) described *Caridinadenticulatavietnamensis* from northern Vietnam. In subsequent publications, he raised this subspecies to species level as *C.vietnamensis* (Dang 1975, Dang et al. 1980). Cai (1996) synonymised it with *Neocaridinapalmatapalmata* (Shen, 1948). Subsequently, Dang and co-authors moved it to the genus *Neocaridina* (*N.vietnamensis*) (Dang and Do 2007b, Dang and Do 2008, Dang and Ho 2012). Li and Liang (2002) followed Cai (1996) to consider *C.denticulatavietnamensis* as junior synonyms of *Neocaridinapalmatapalmata* (Shen, 1948). However, Dang and Do (2008) and Dang and Ho (2012) stated that *N.vietnamensis* can be distinguished from *N.palmata* or a subspecies of this species by the endopod of the male first pleopod (round shape and stable vs. variable); appendix masculina of male second pleopod (perpendicular vs. rounded) and appendix interna (reaching nearly to the tip of appendix masculina vs. not reaching to the tip of appendix masculina) and larger egg size (1–1.25 × 0.65–0.85 mm) (Dang and Do 2008, Dang and Ho 2012). These authors also mentioned the differences in the endopod of the first pleopod and the appendices masculina and interna. Nevertheless, Liang (2004) stated that the shape of the endopod of the first pleopod is very variable in *N.palmata*, even in the specimens collected from the same localities. According to this author, *N.palmata* can be distinguished from *N.denticulata* De Haan, 1849 by the third pereiopod. In both sexes, it differs in length and shape (vs. length and shape of third pereiopod being similar between male and female); the dactylus and distal portion of the propodus of the third pereiopod of male is inflated (vs. the dactylus and distal portion of the propodus of the third pereiopod of the male is not inflated); the propodus of the third pereiopod of the male is curved (vs. the propodus of the third pereiopod of the male is straight). He also noted that this species lives in streams, rivers, reservoirs, ditches and small ponds. It is distributed in the middle reaches of the Yangtze River and central China, south and southwest China and North Vietnam. Due to its wide distribution, humid climate and different living environments, the appendix masculina often shows morphological variation and subspecies differentiation. Our specimens collected from different sampling sites in northeast Vietnam showed that all of them belong to the subspecies *N.palmatapalmata*.

## Analysis

We clarified four species of atyid freshwater shrimps in Vietnam, three from the genus *Caridina* (*C.cantonensis*, *C.lanceifrons* and *C.serrata*) and one from the genus *Neocaridina* (*N.palmatapalmata*) that were originally described from China (Table 1). We considered *Caridinaflavilineata* Dang, 1975, *C.vietriensis* Dang & Do, 2007 and *C.pseudoflavilineata* Do & Dang, 2010 as synonyms of *C.lanceifrons* Yu, 1936 and *Neocaridinavietnamensis* Dang, 1975 as a synonym of *N.palmatapalmata* (Shen, 1948). Egg-size and distribution data (Table 1) suggest abbreviated larval development and a complete life cycle in freshwater (Vogt 2013). In general, in Vietnam, all four species are confined to North Vietnam (Fig. 1). It is rather surprising that the only species amongst these extending further south (*Caridinalanceifrons* in southern Thailand) does not occur in southern Vietnam.

## Supplementary Material

7E90F4EE-3A38-5719-ADA7-7B9EEC058299 10.3897/BDJ.9.e70289.suppl1 Supplementary material 1 New occurrence data of four species of atyid shrimps in Vietnam. Data type Occurrences Brief description The data were created from the field surveys from 2003 to 2020. The collection dates and collectors of several samples could not be identified because their labels were lost. File: oo_523308.txt https://binary.pensoft.net/file/523308 Do Van Tu, Kristina von Rintelen, Werner Klotz, Le Hung Anh, Tran Anh Tuan, Dang Van Dong, Nguyen Tong Cuong, Phan Thi Yen, Hoang Ngoc Khac, Phan Doan Dang, Thomas von Rintelen

XML Treatment for Caridina cantonensis

XML Treatment for Caridina lanceifrons

XML Treatment for Caridina serrata

XML Treatment for Neocaridina palmata palmata

## Figures and Tables

**Figure 1a.**
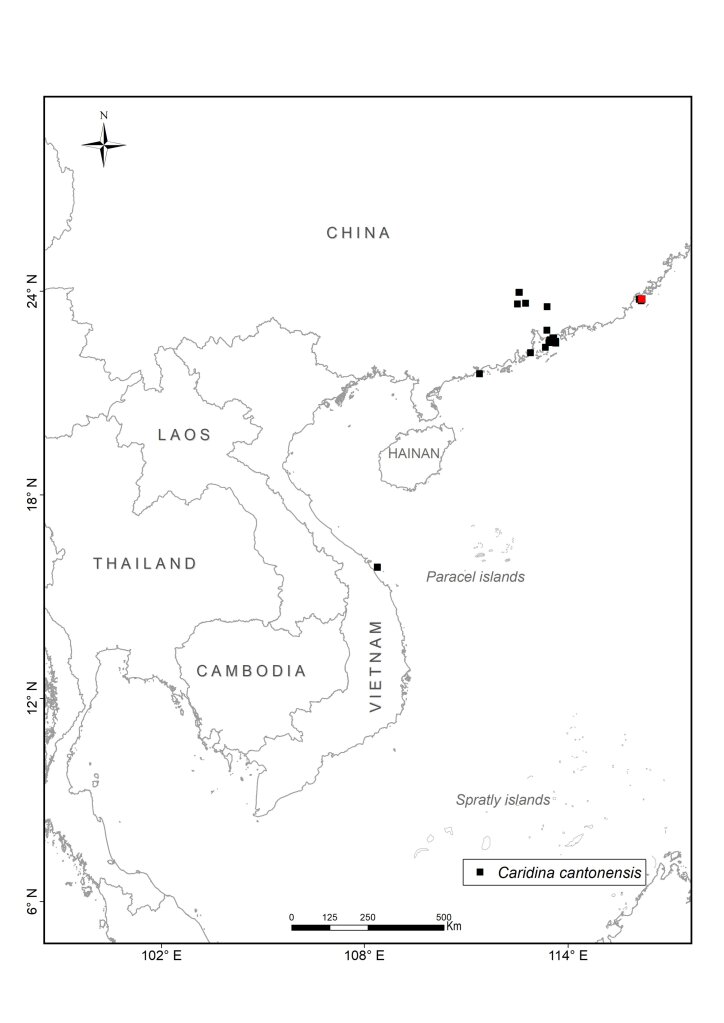
Caridina cantonensis

**Figure 1b.**
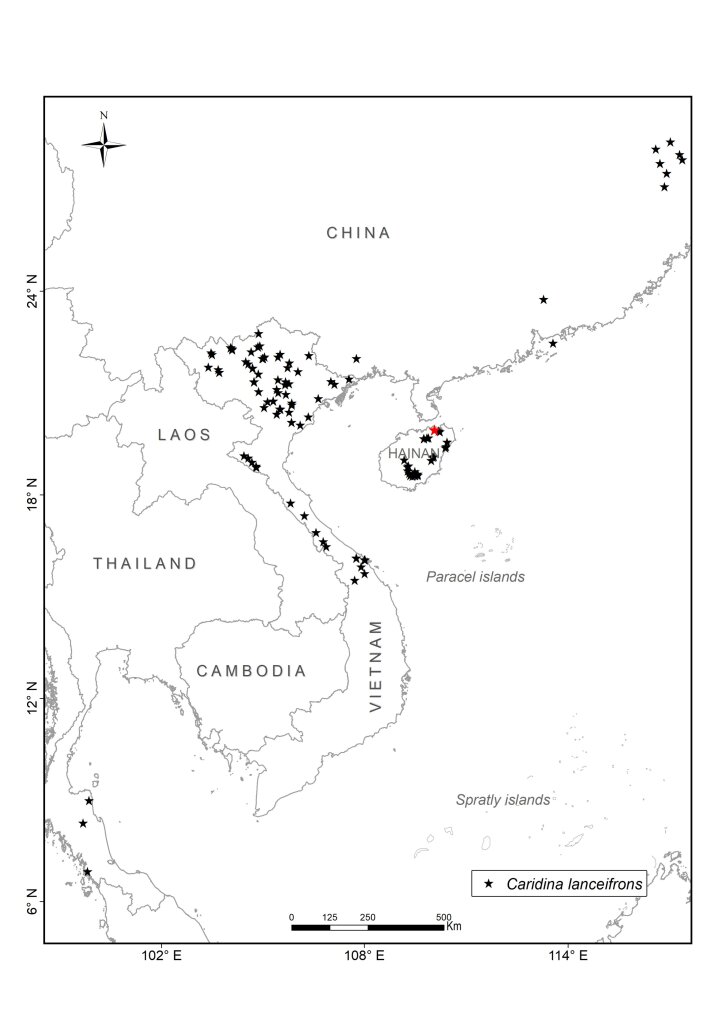
Caridina lanceifrons

**Figure 1c.**
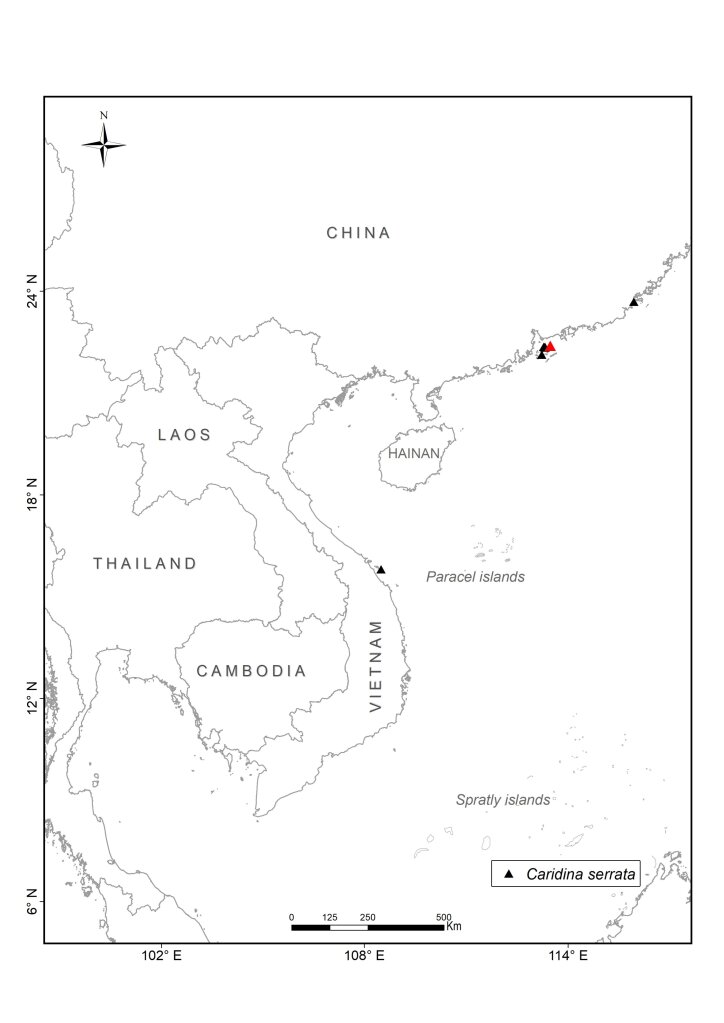
Caridina serrata

**Figure 1d.**
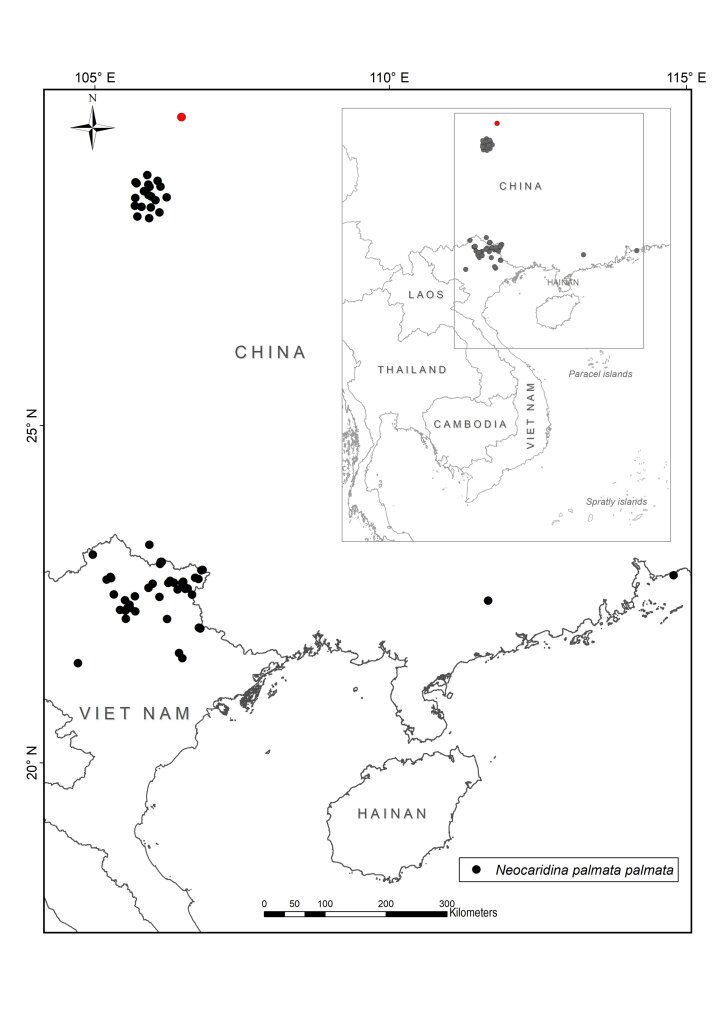
Neocaridina palmata palmata

**Figure 2a.**
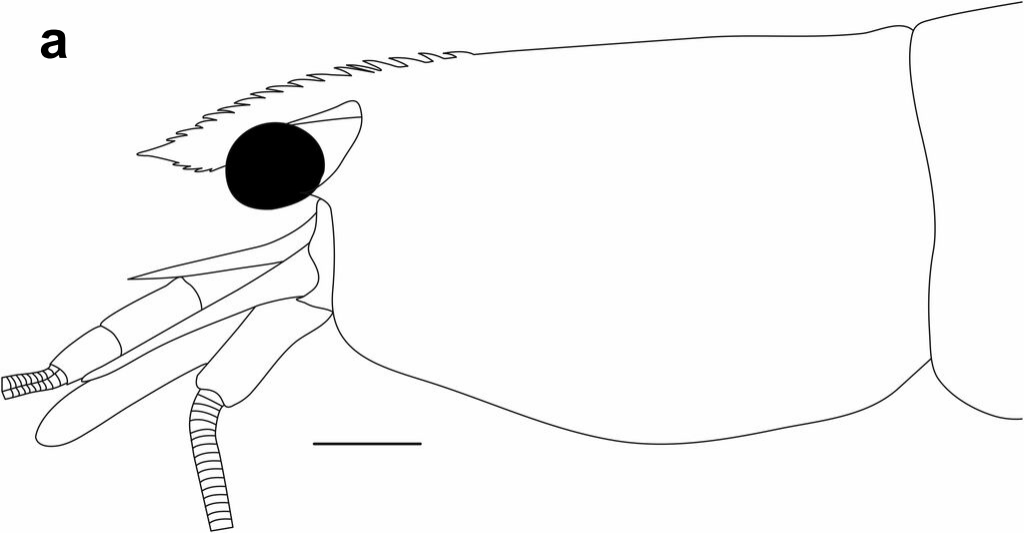
Cephalothorax and cephalic appendages

**Figure 2b.**
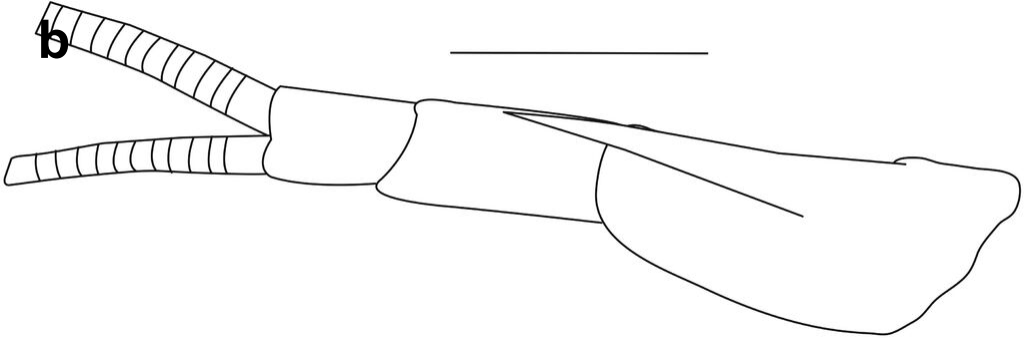
Antennular peduncle

**Figure 2c.**
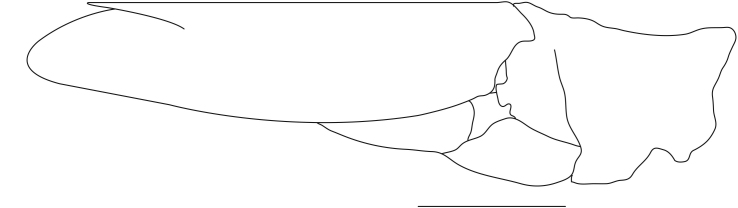
Scaphocerite

**Figure 2d.**
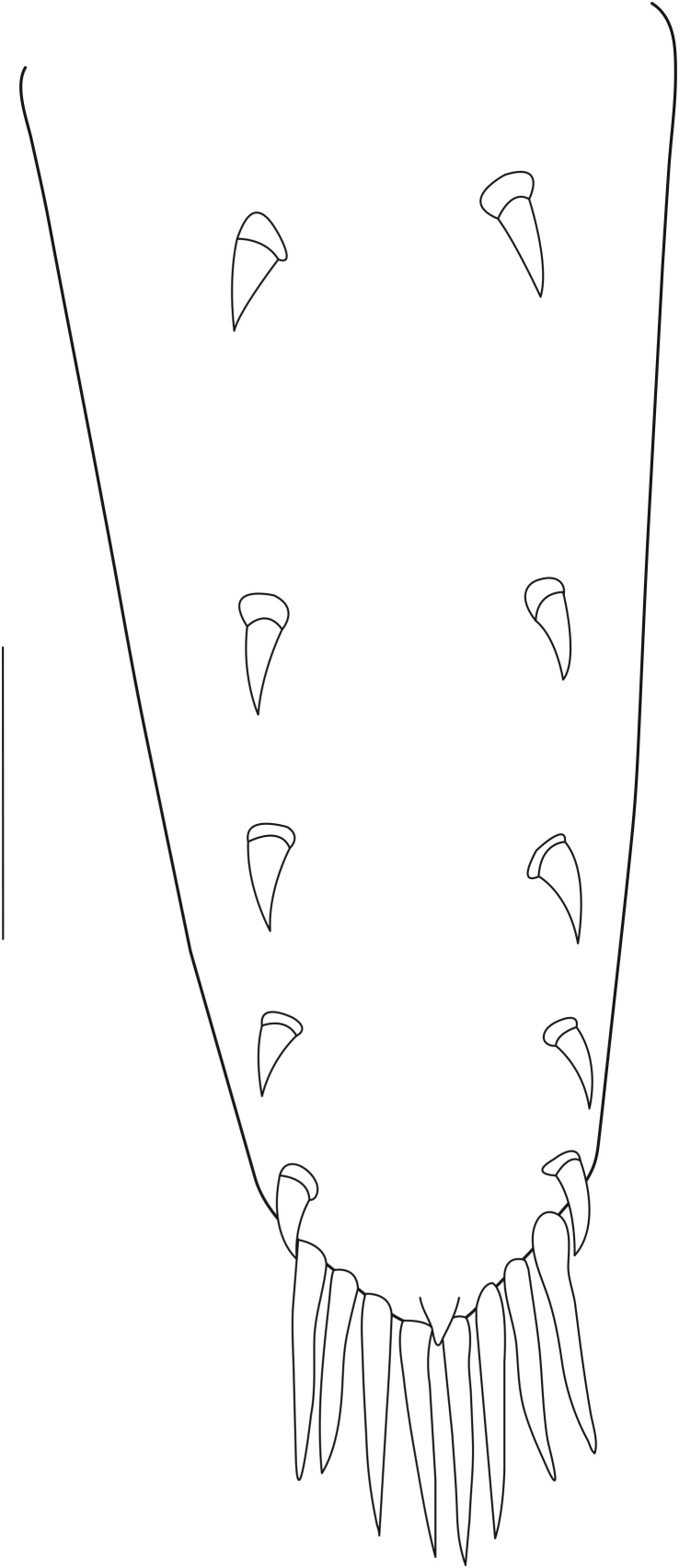
Telson

**Figure 2e.**
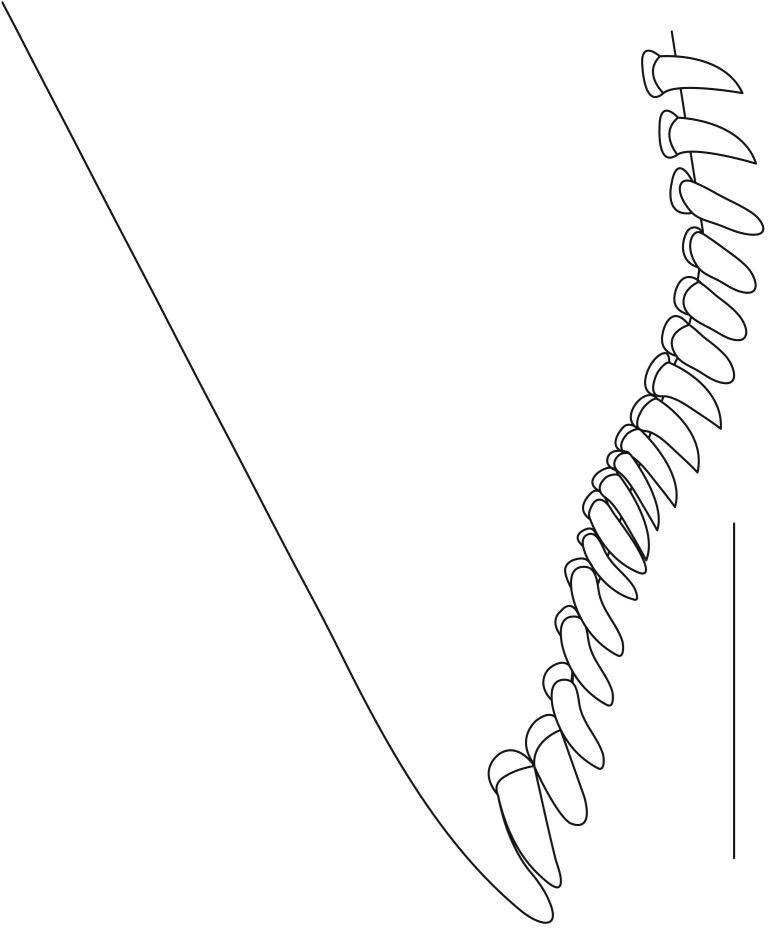
Uropodal diaeresis.

**Figure 3a.**
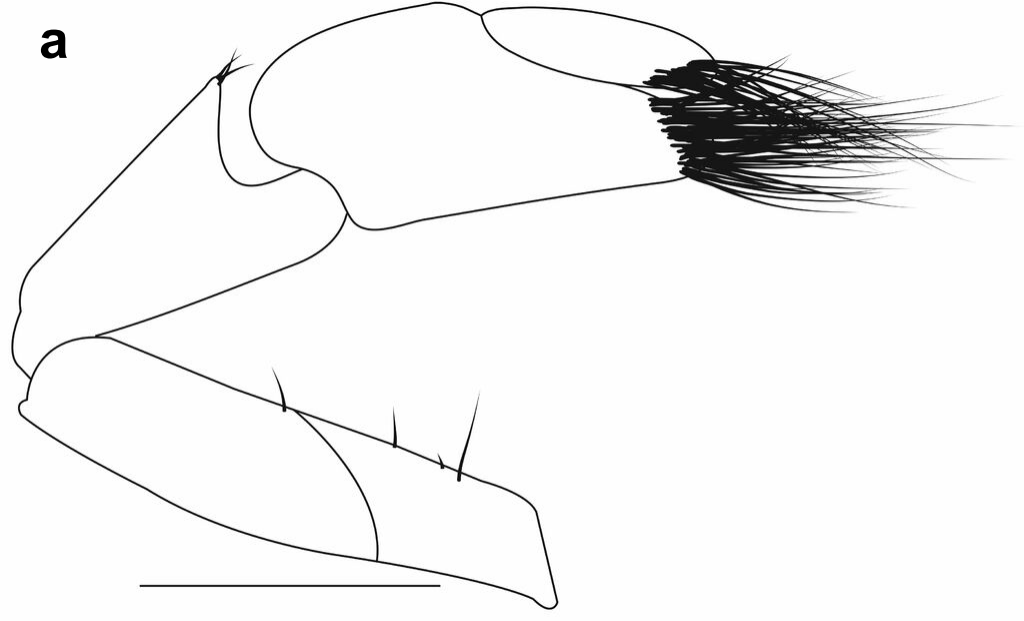
First pereiopod

**Figure 3b.**
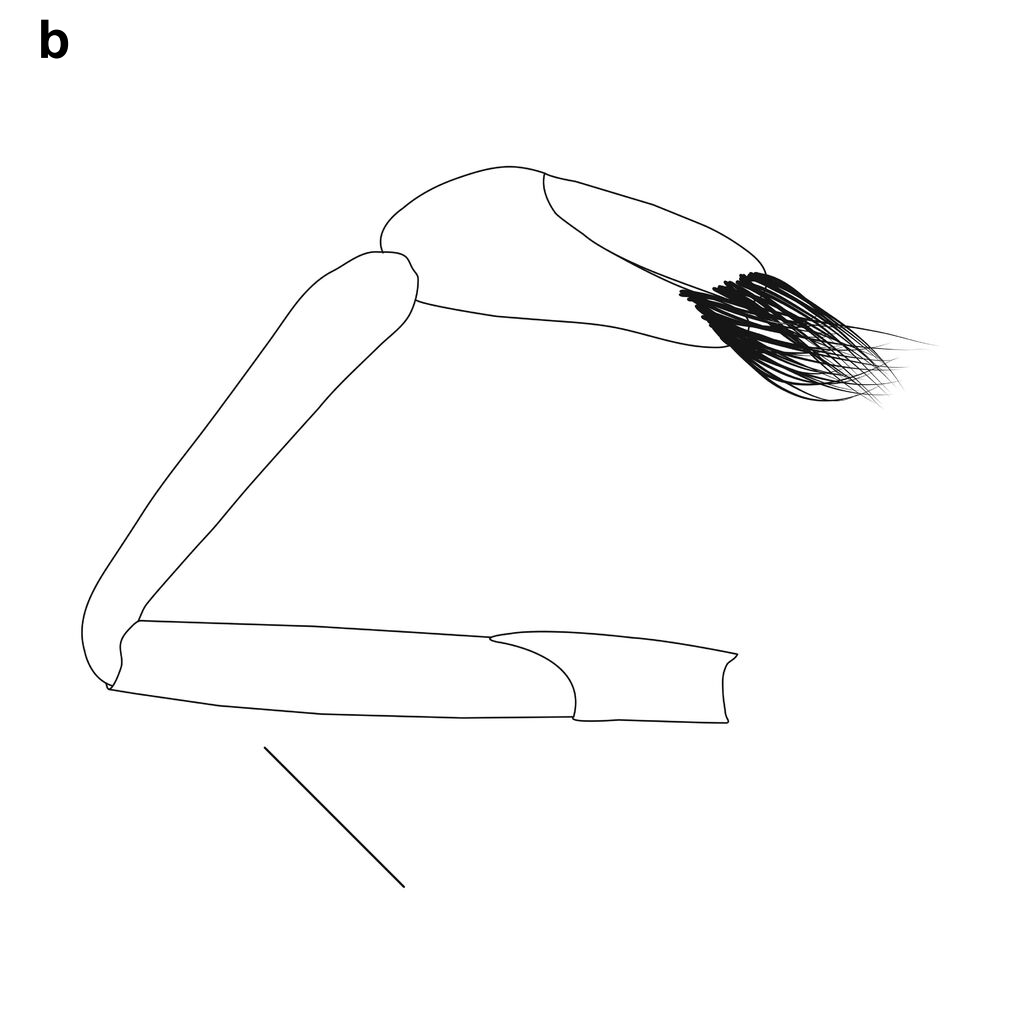
Second pereiopod

**Figure 3c.**
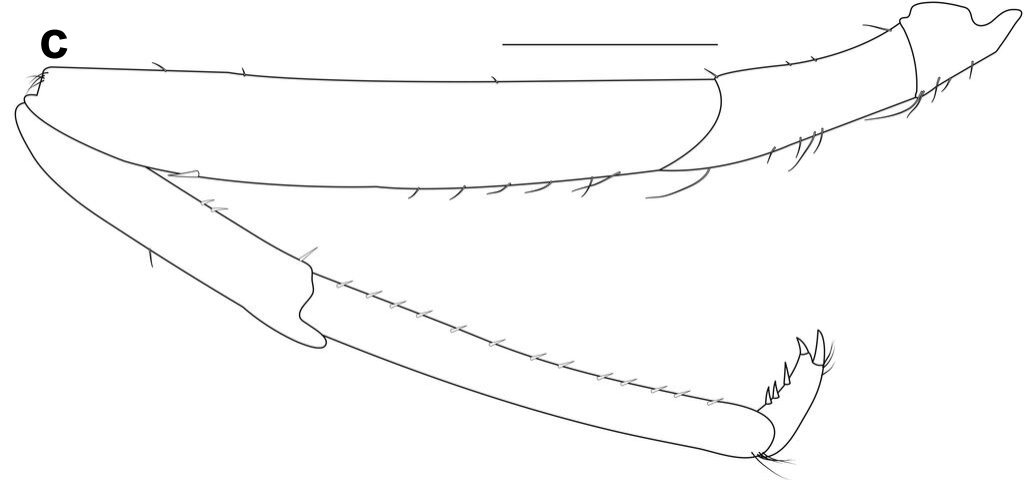
Third pereiopod

**Figure 3d.**
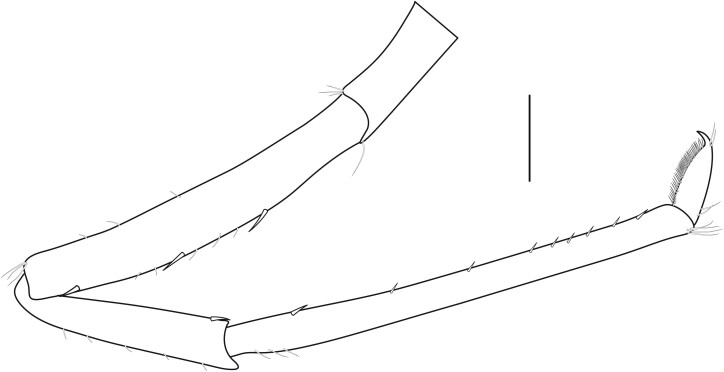
Fifth pereiopod

**Figure 3e.**
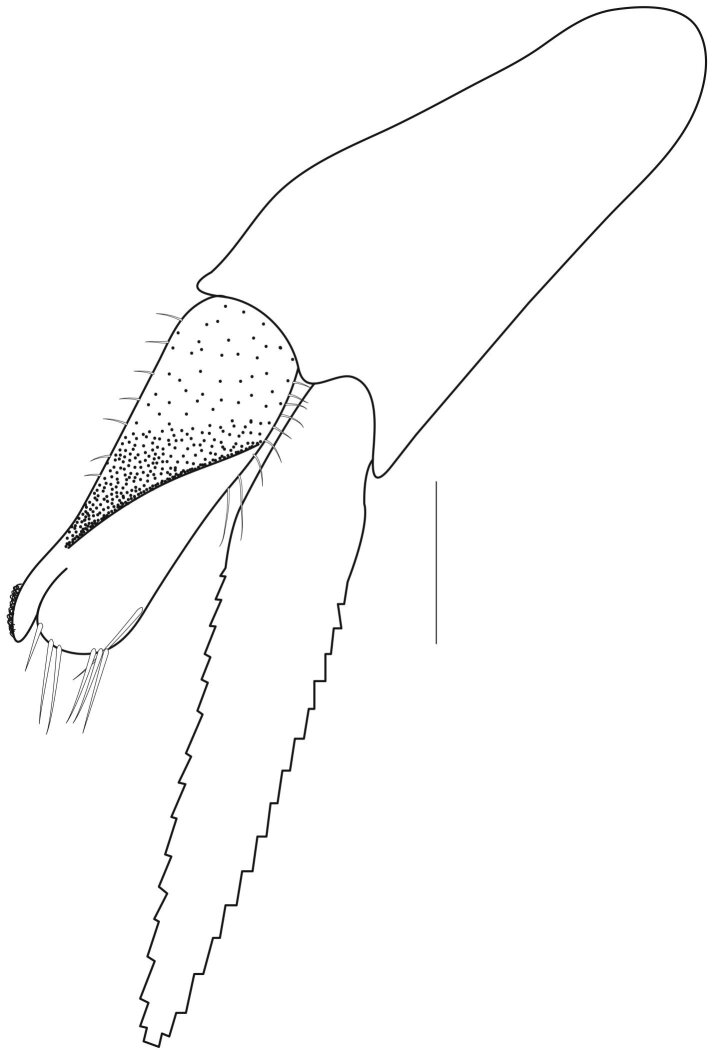
Male first pleopod

**Figure 3f.**
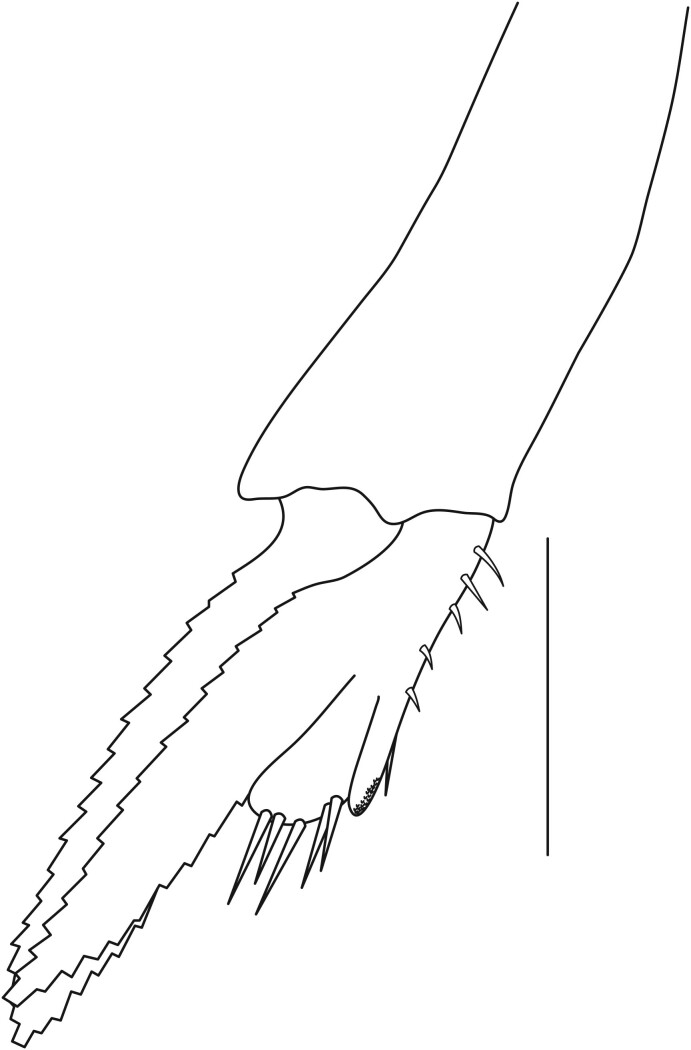
Male second pleopod.

**Figure 5a.**
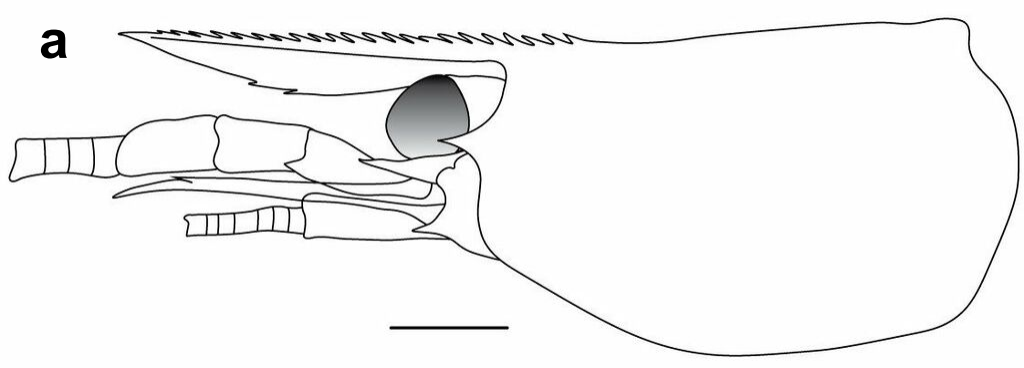
Cephalothorax and cephalic appendages, lateral view

**Figure 5b.**
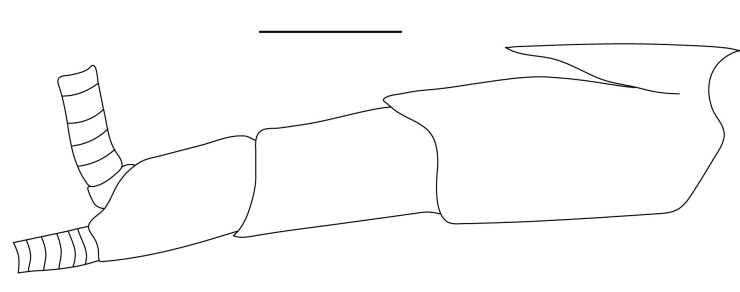
Antennular peduncle

**Figure 5c.**
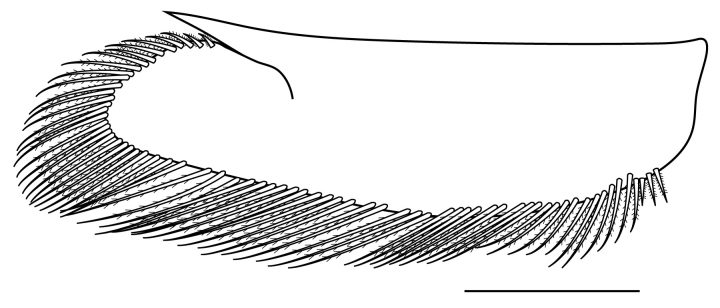
Scaphocerite

**Figure 5d.**
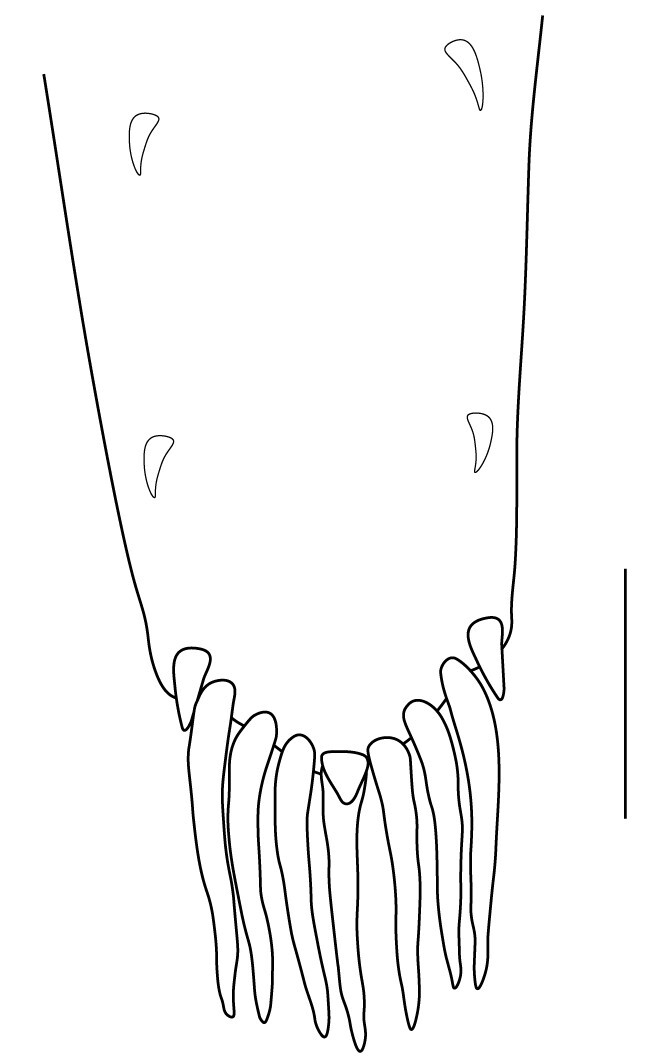
Telson

**Figure 5e.**
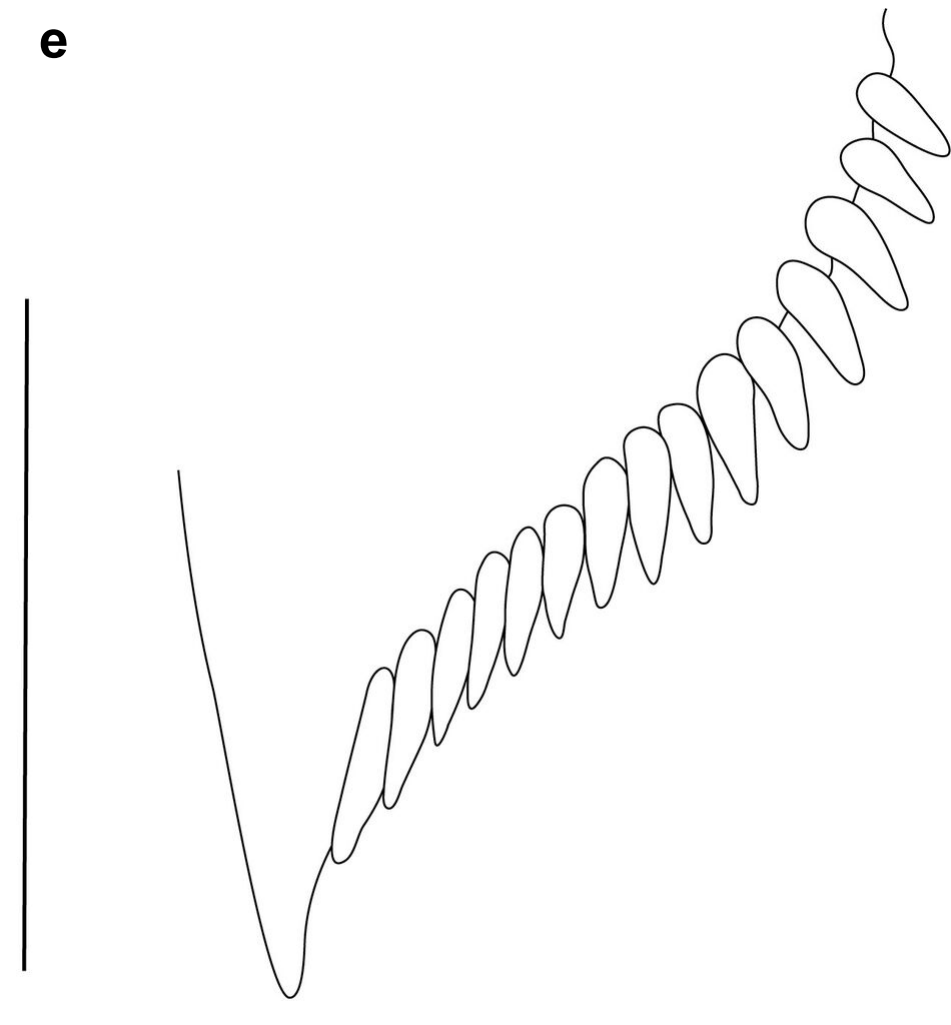
Uropodal diaeresis.

**Figure 6a.**
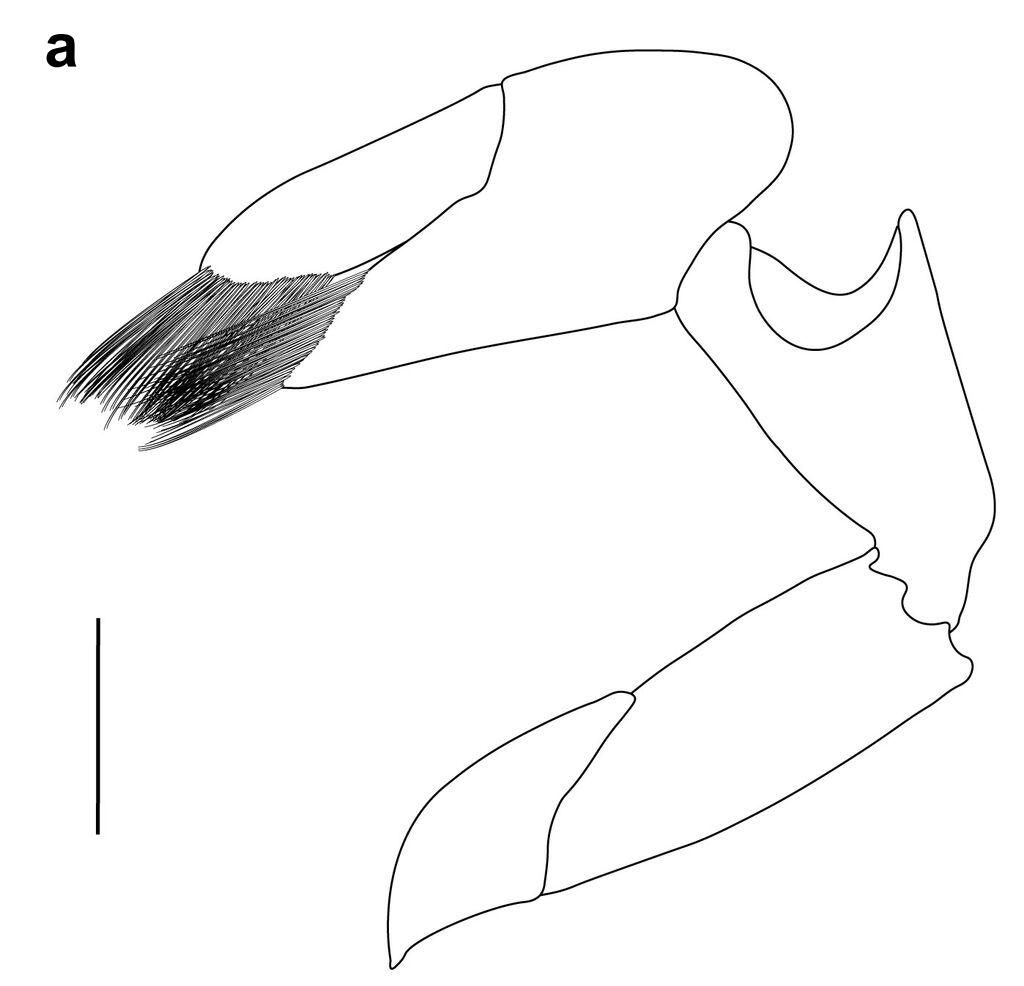
First pereiopod

**Figure 6b.**
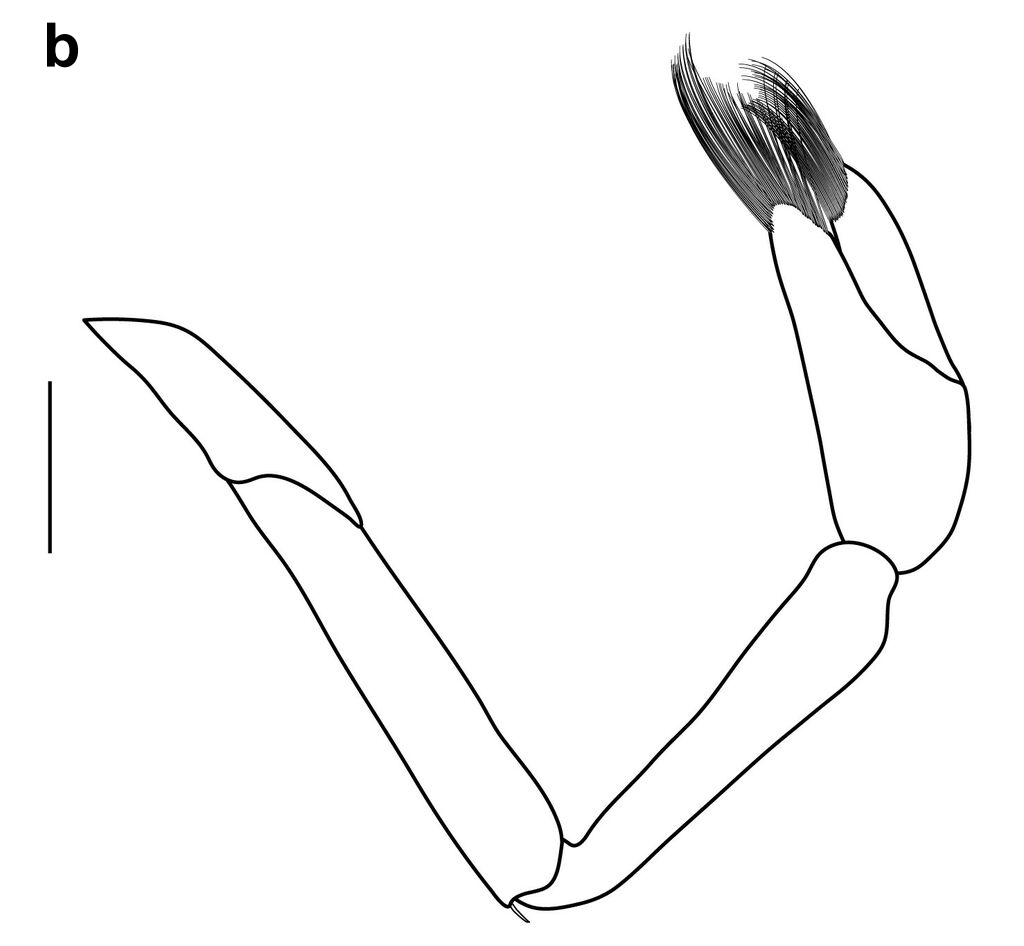
Second pereiopod

**Figure 6c.**
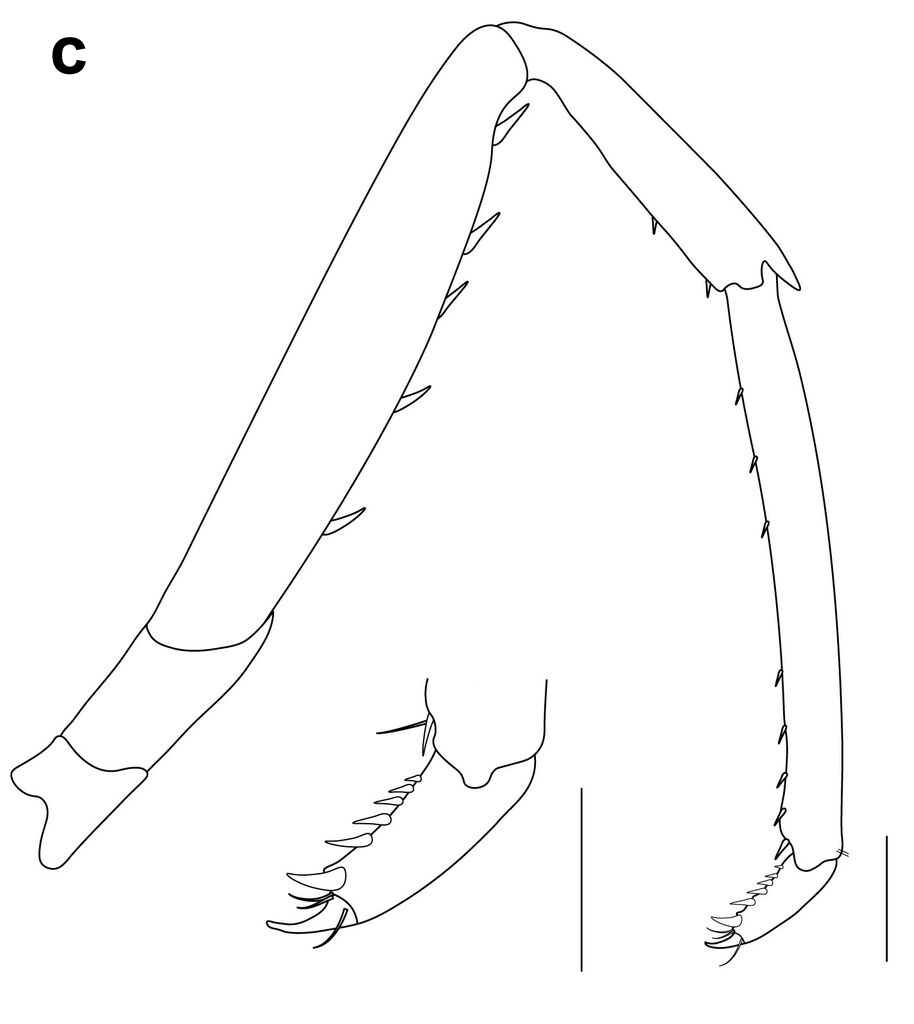
Third pereiopod dactylus of third pereiopod

**Figure 6d.**
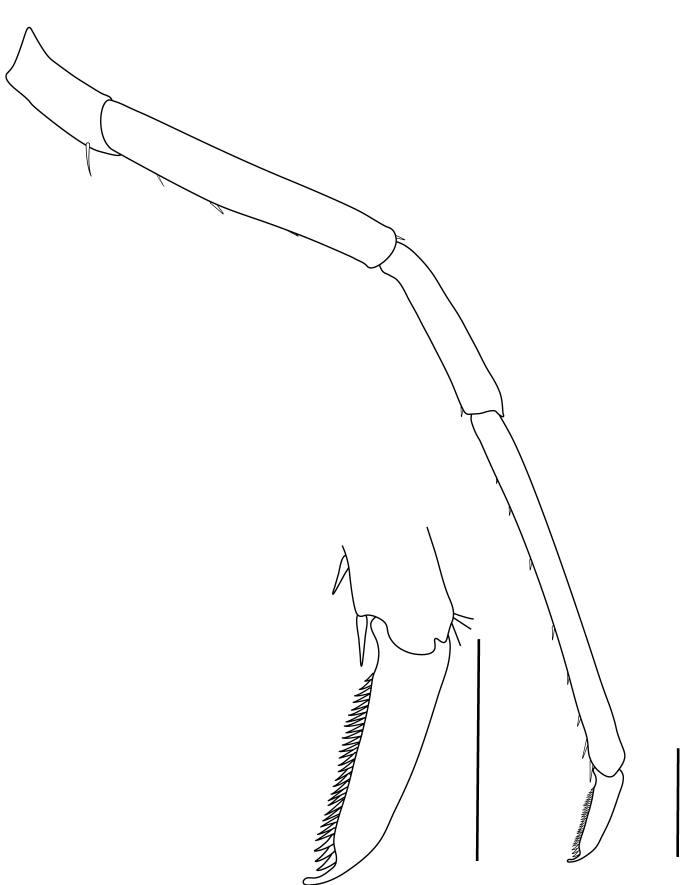
Fifth pereiopod and dactylus of fifth pereiopod

**Figure 6e.**
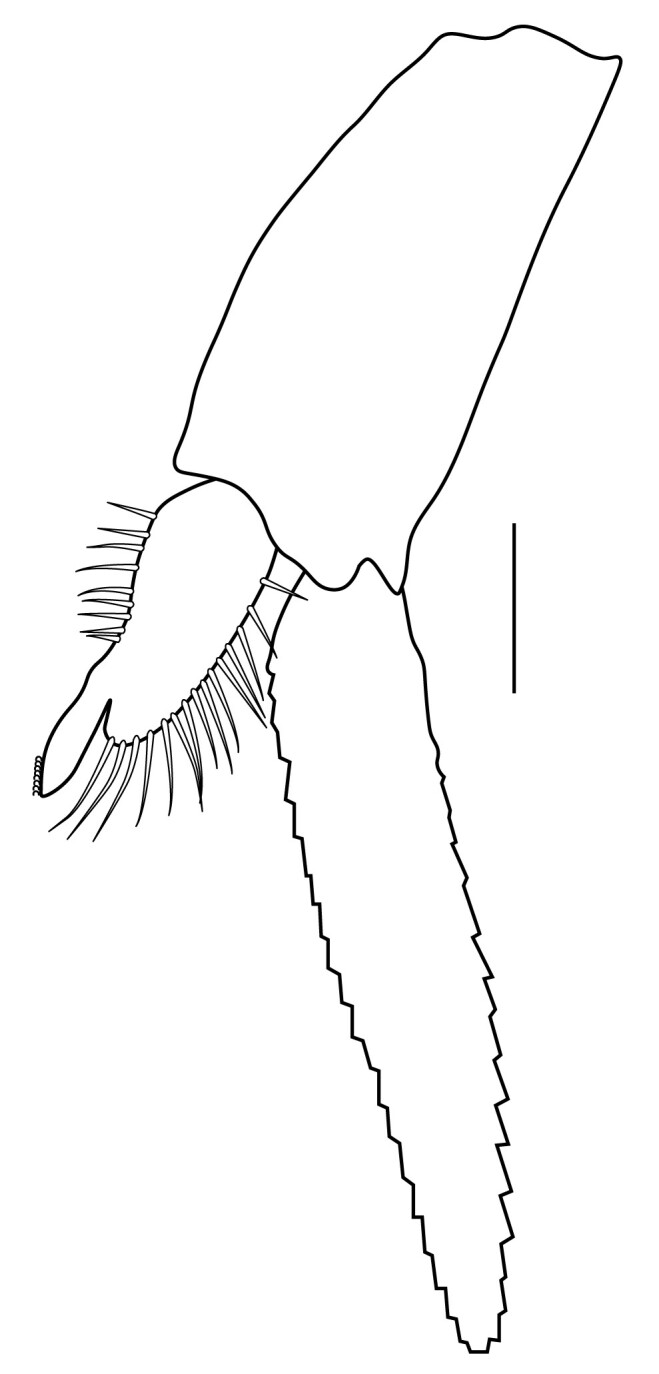
Male first pleopod

**Figure 6f.**
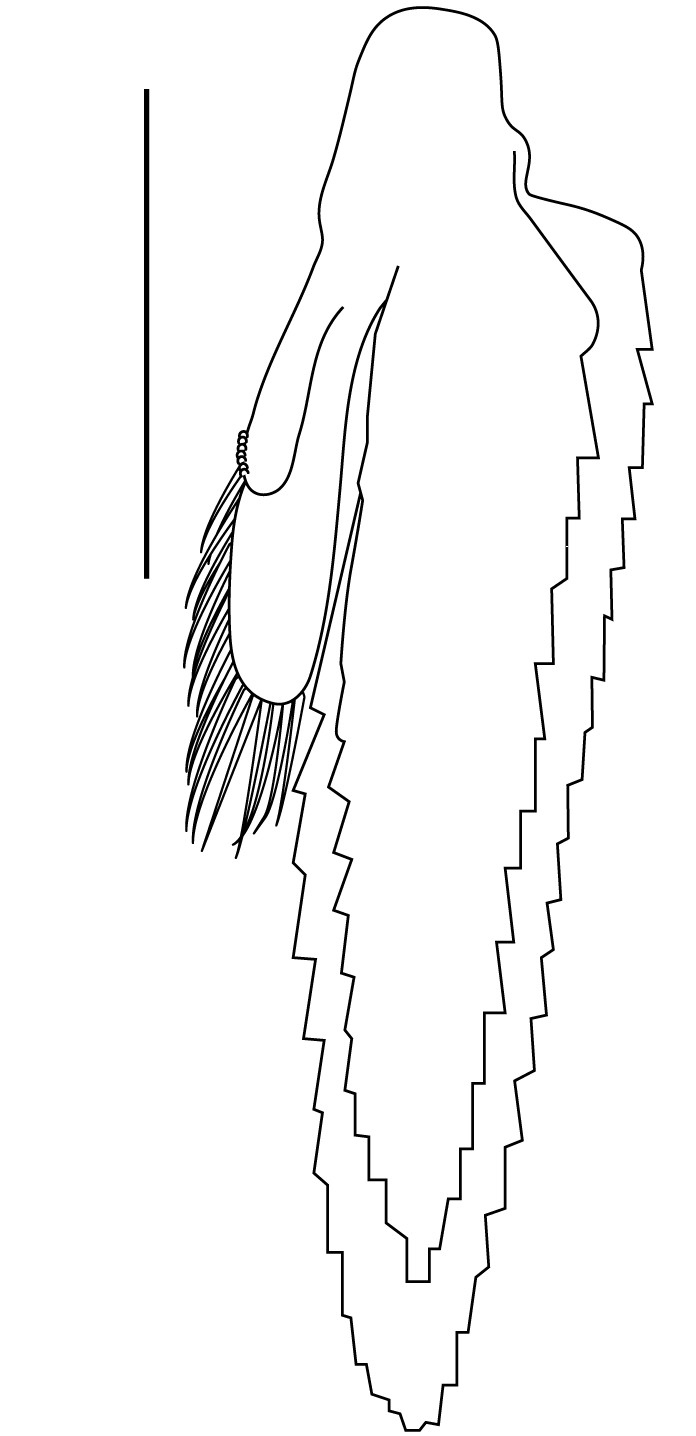
Male second pleopod.

**Figure 7a.**
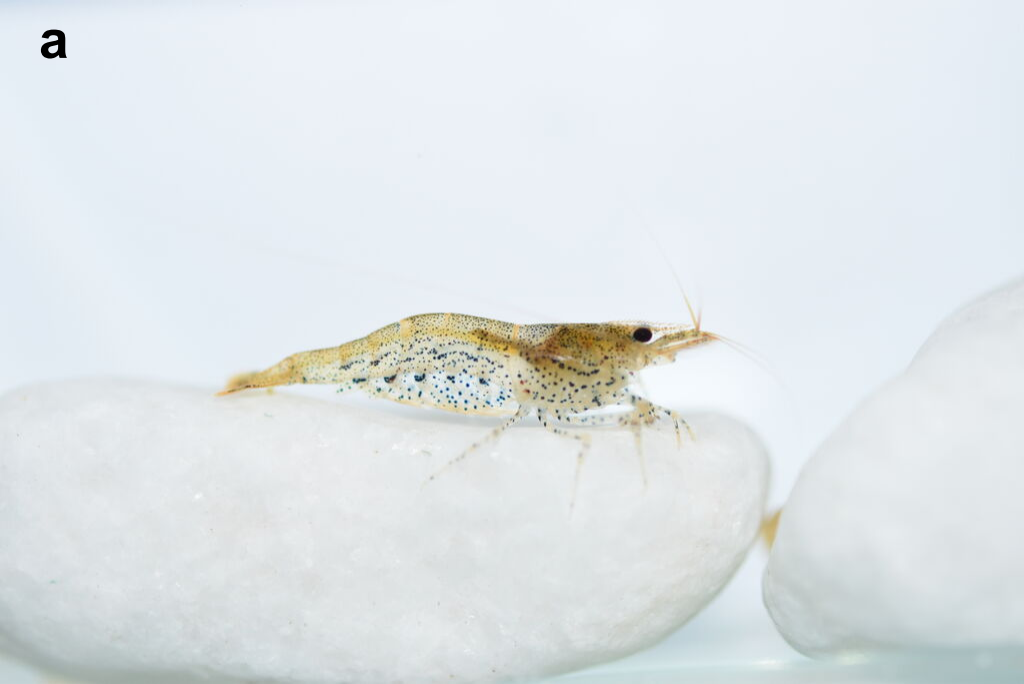
Body yellowish

**Figure 7b.**
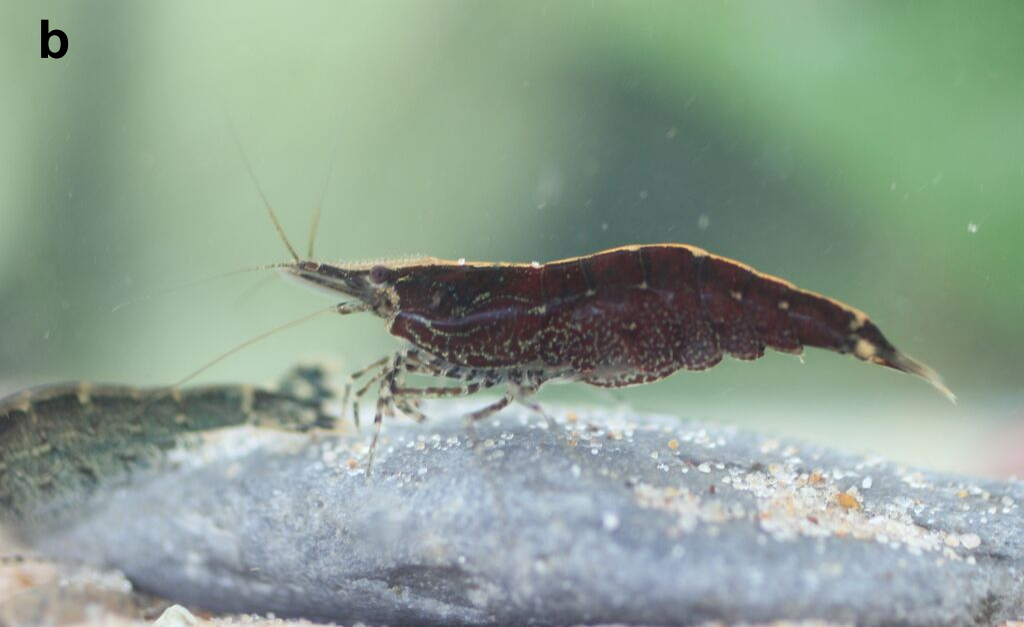
Female body dark green with dorsal yellow stripe.

**Figure 8a.**
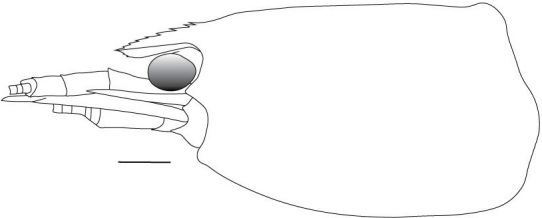
Cephalothorax and cephalic appendages, lateral view

**Figure 8b.**
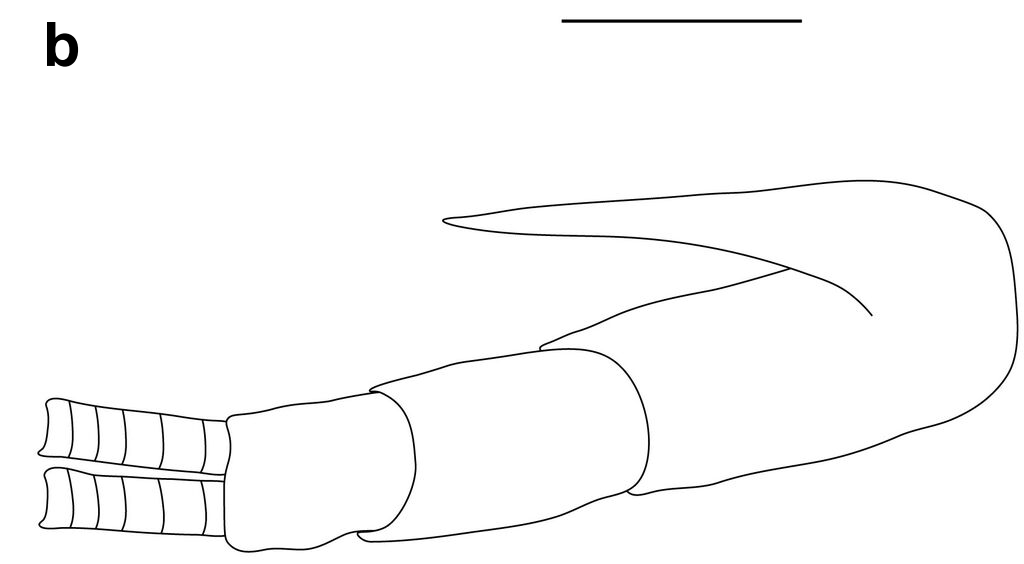
Antennular peduncle

**Figure 8c.**
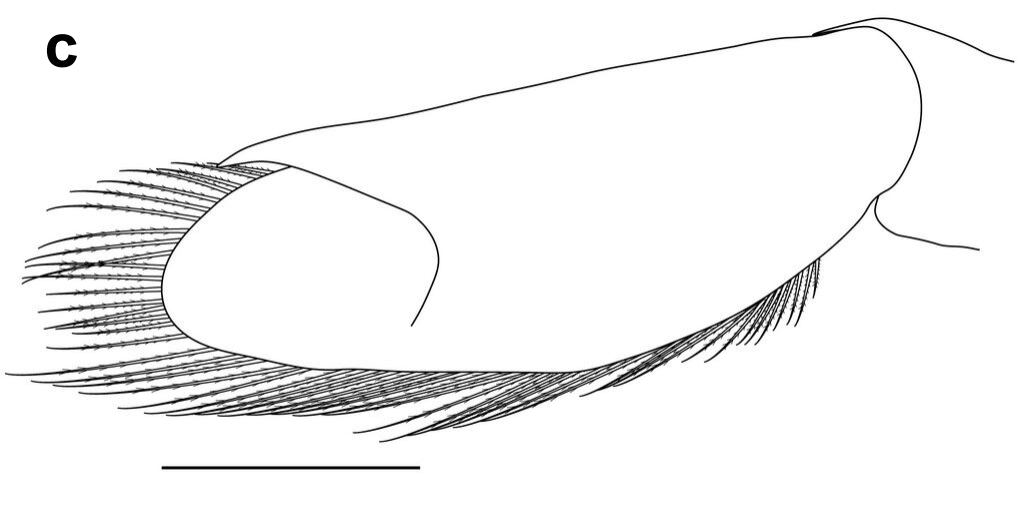
Scaphocerite

**Figure 8d.**
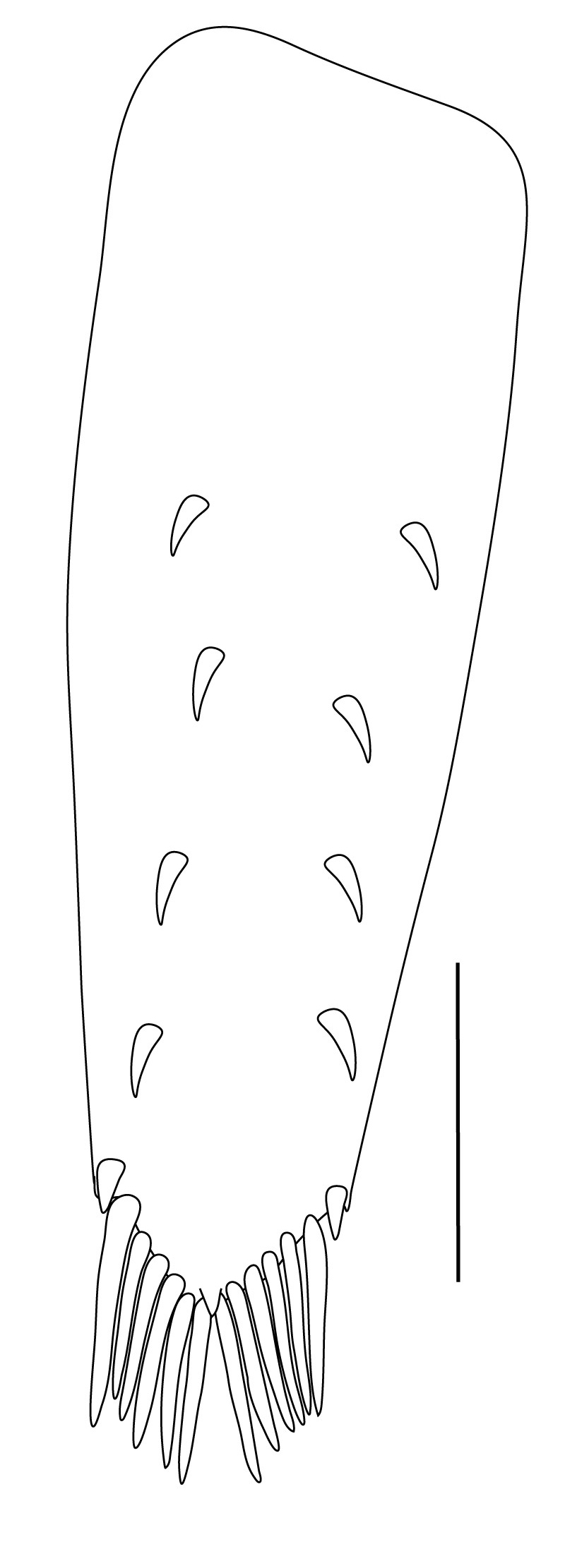
Telson

**Figure 8e.**
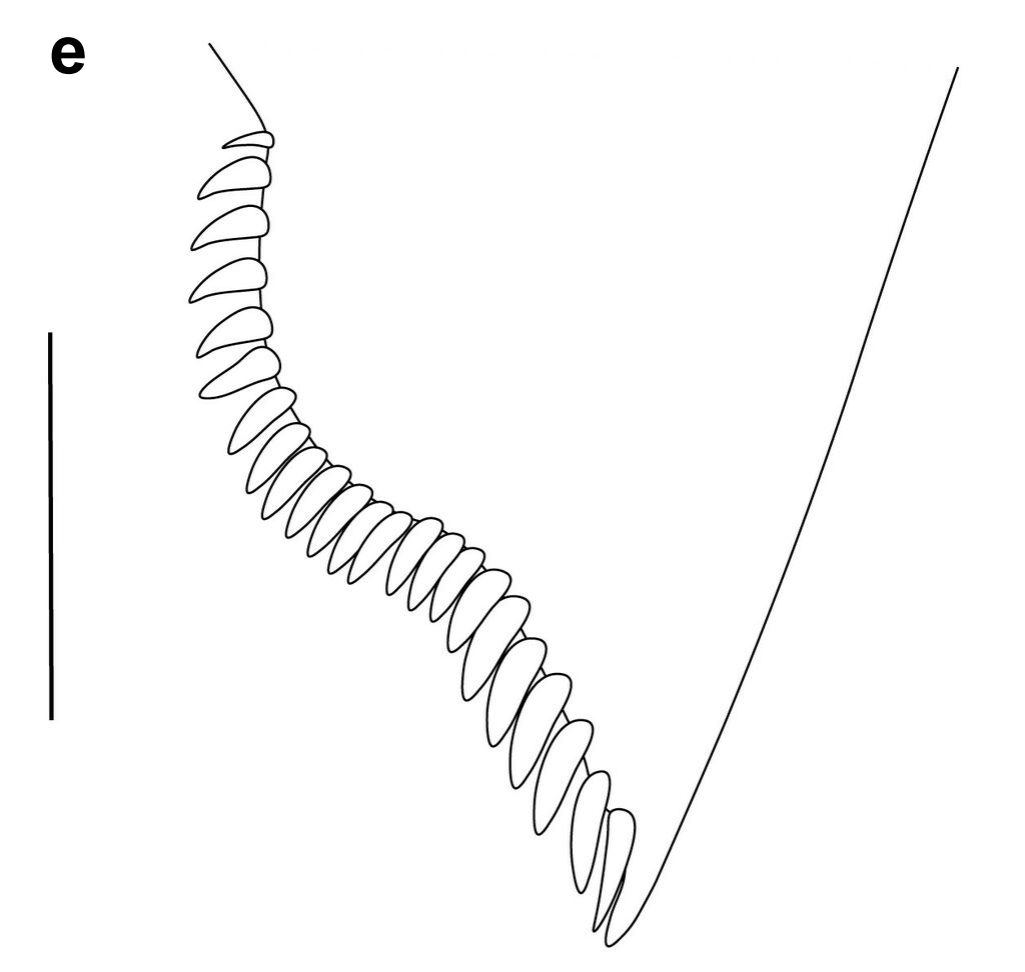
Uropodal diaeresis

**Figure 9a.**
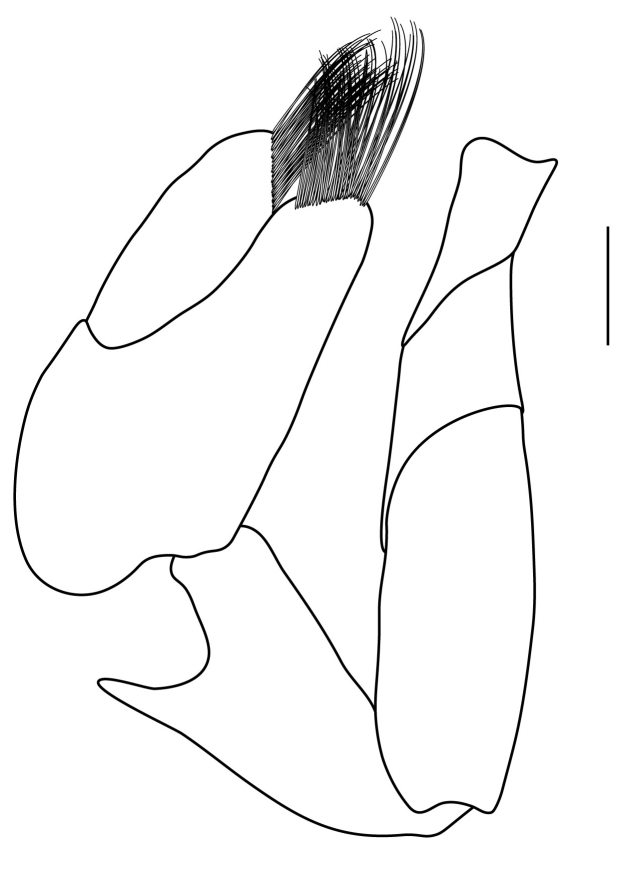
First pereiopod

**Figure 9b.**
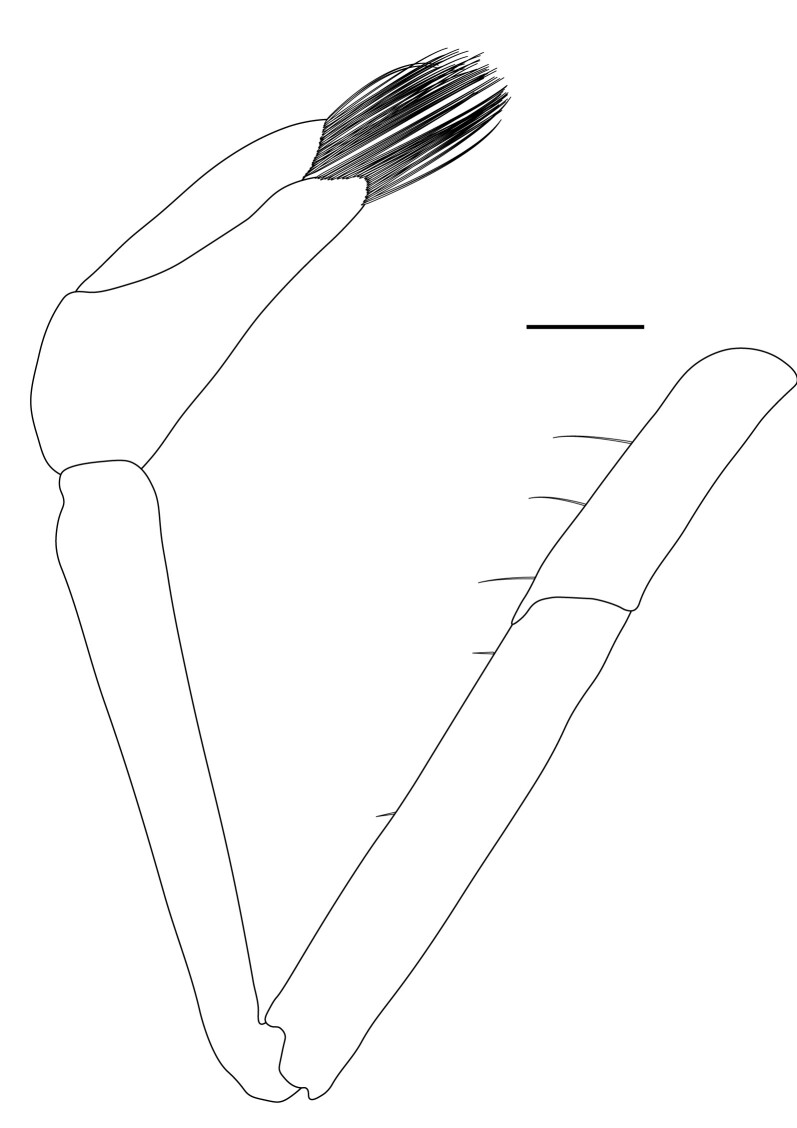
Second pereiopod

**Figure 9c.**
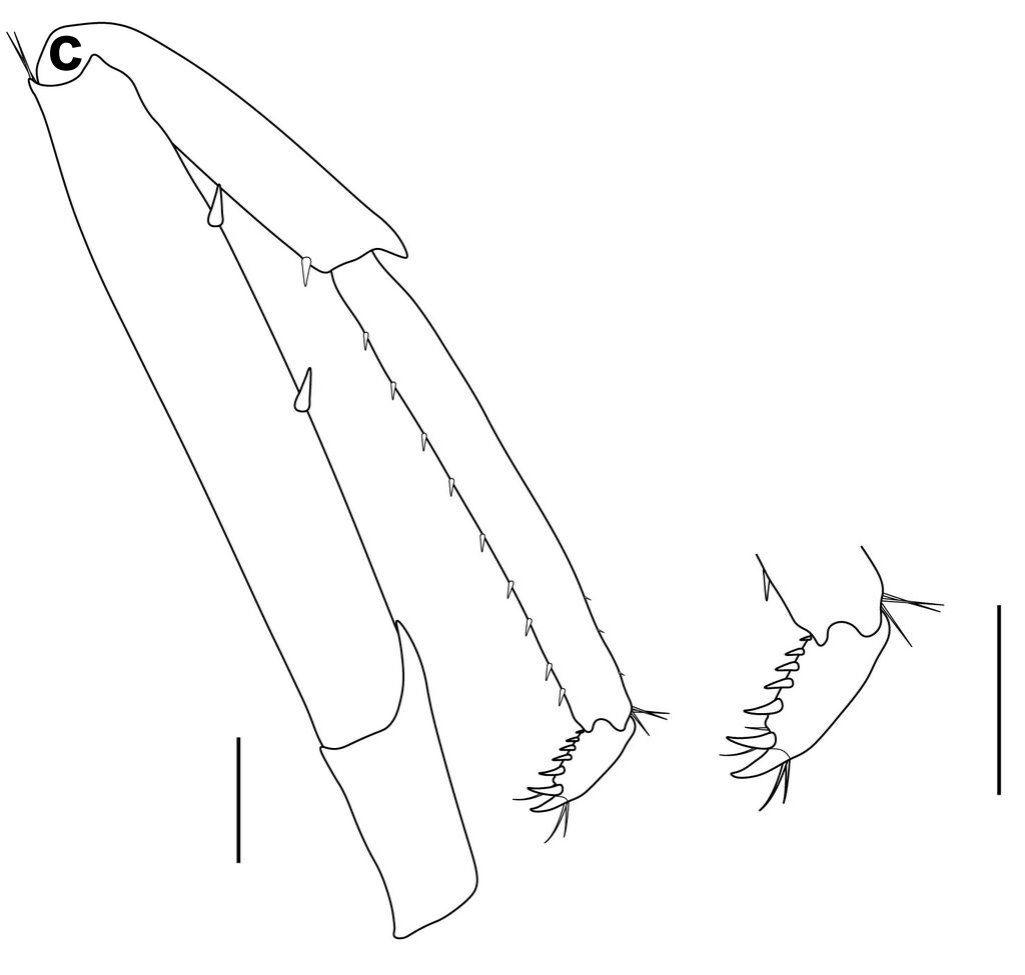
Third pereiopod and dactylus of third pereiopod

**Figure 9d.**
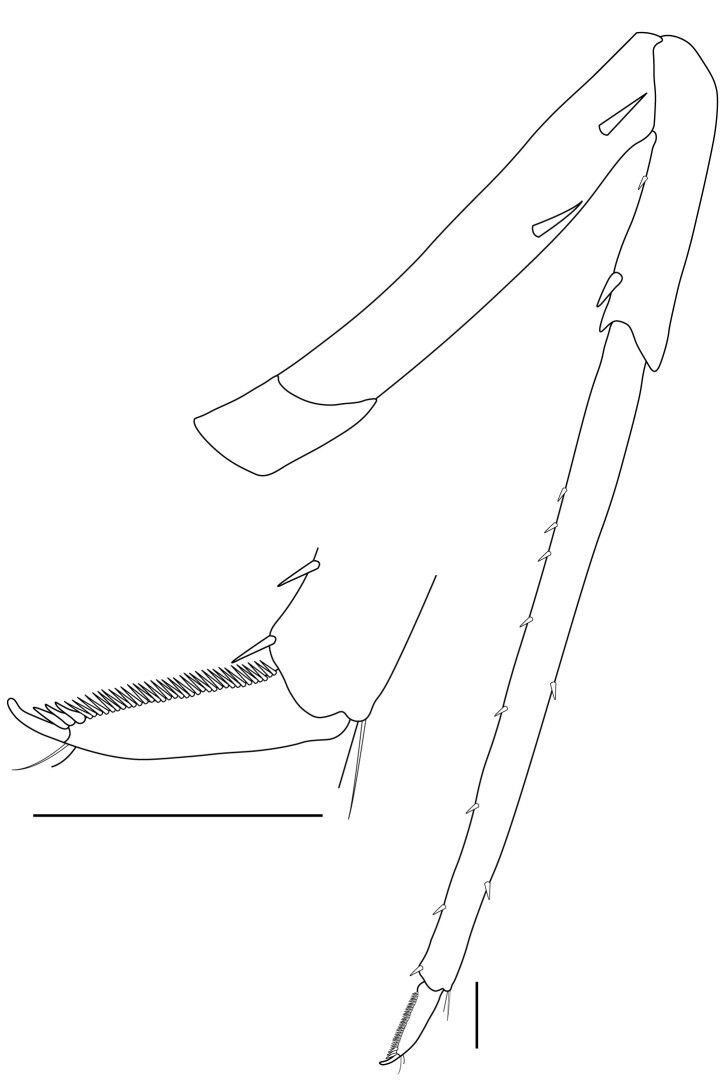
Fifth pereiopod and dactylus of fifth pereiopod

**Figure 9e.**
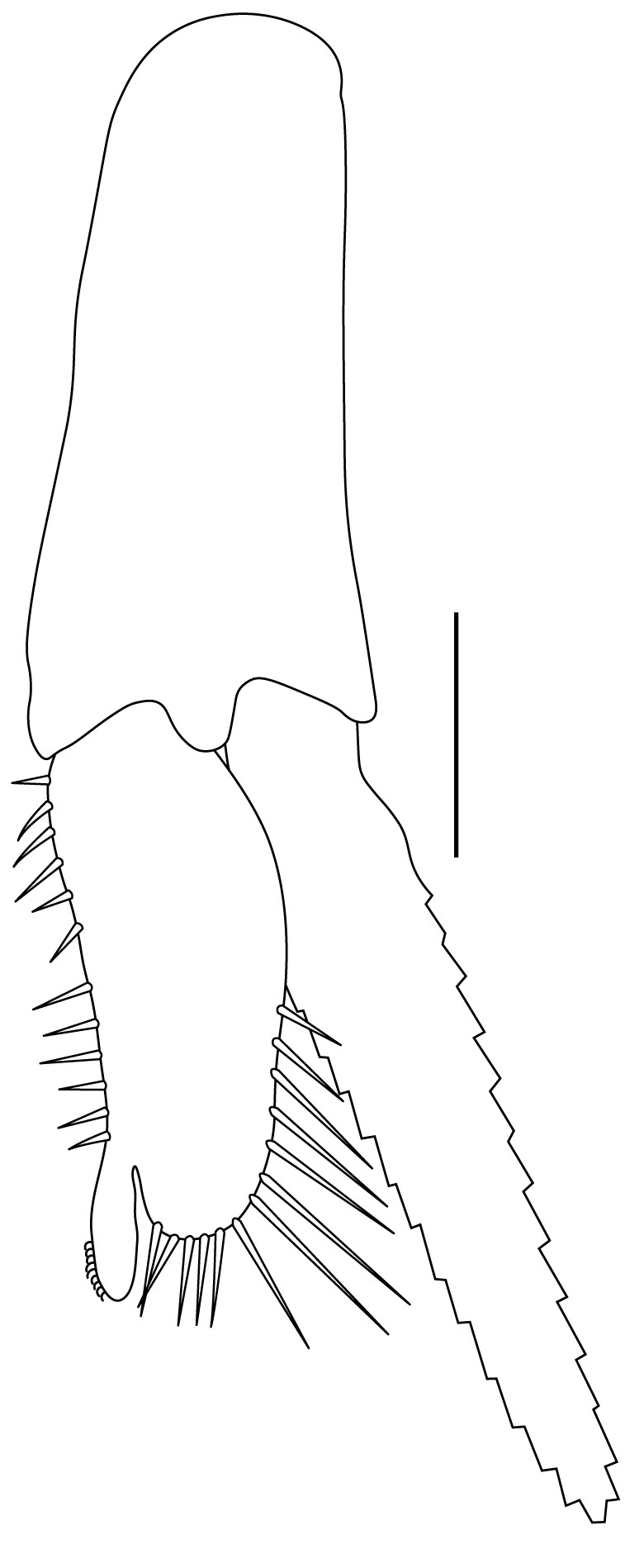
Male first pleopod

**Figure 9f.**
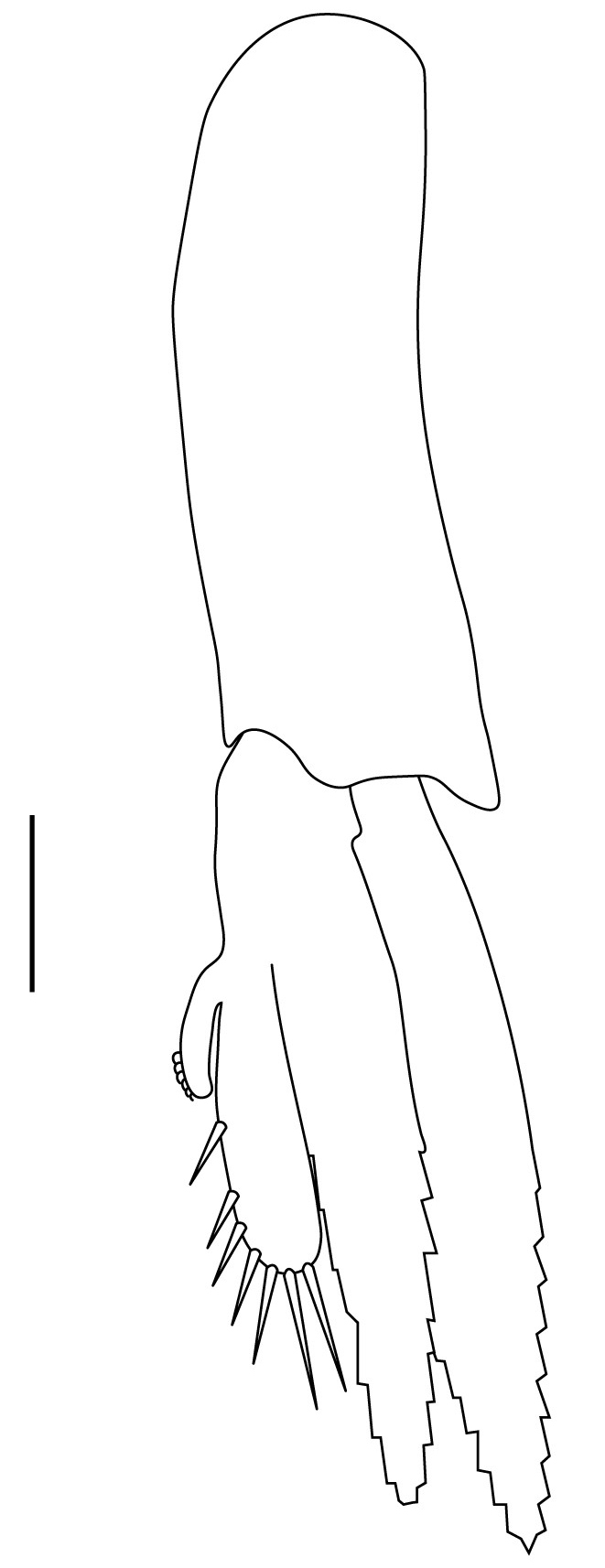
Male second pleopod.

**Figure 10a.**
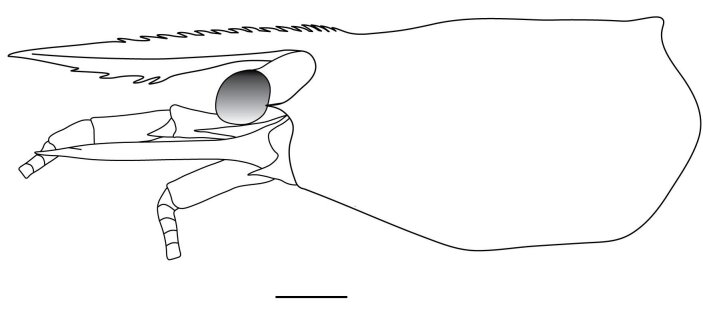
Cephalothorax and cephalic appendages, lateral view

**Figure 10b.**
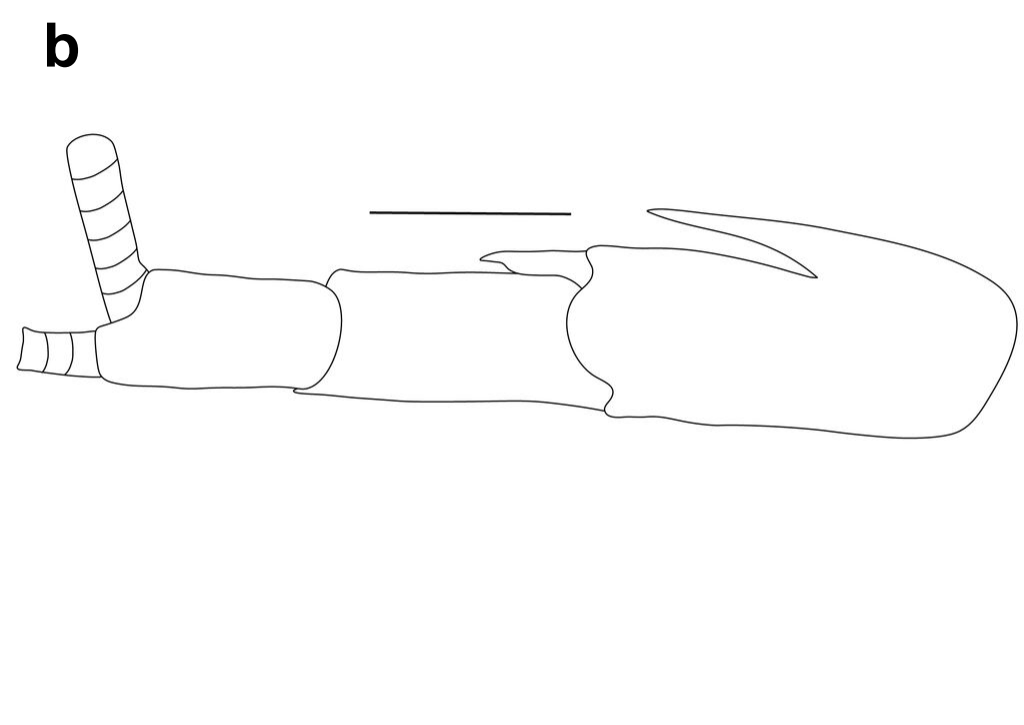
Antennular peduncle

**Figure 10c.**
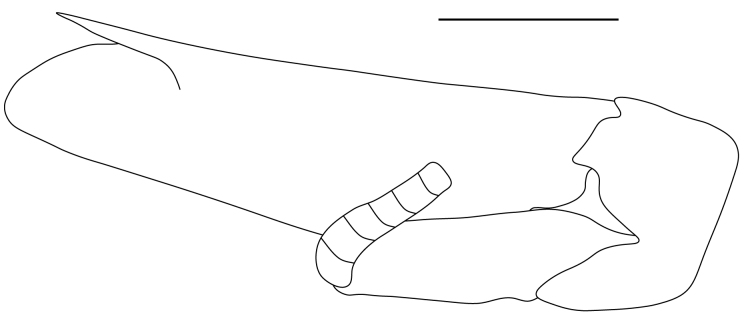
Scaphocerite

**Figure 10d.**
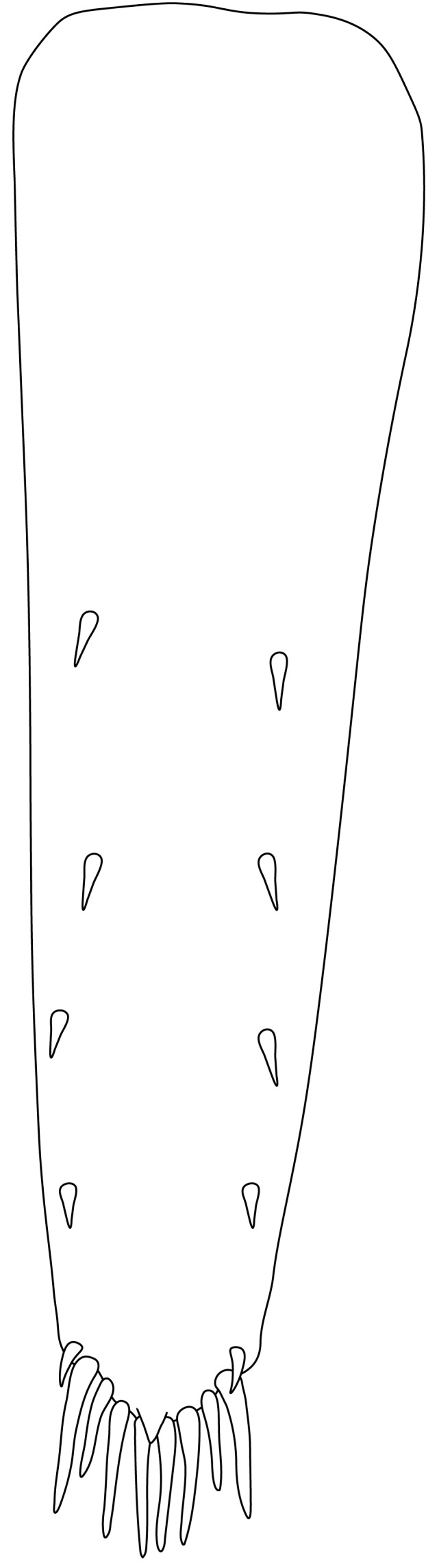
Telson

**Figure 10e.**
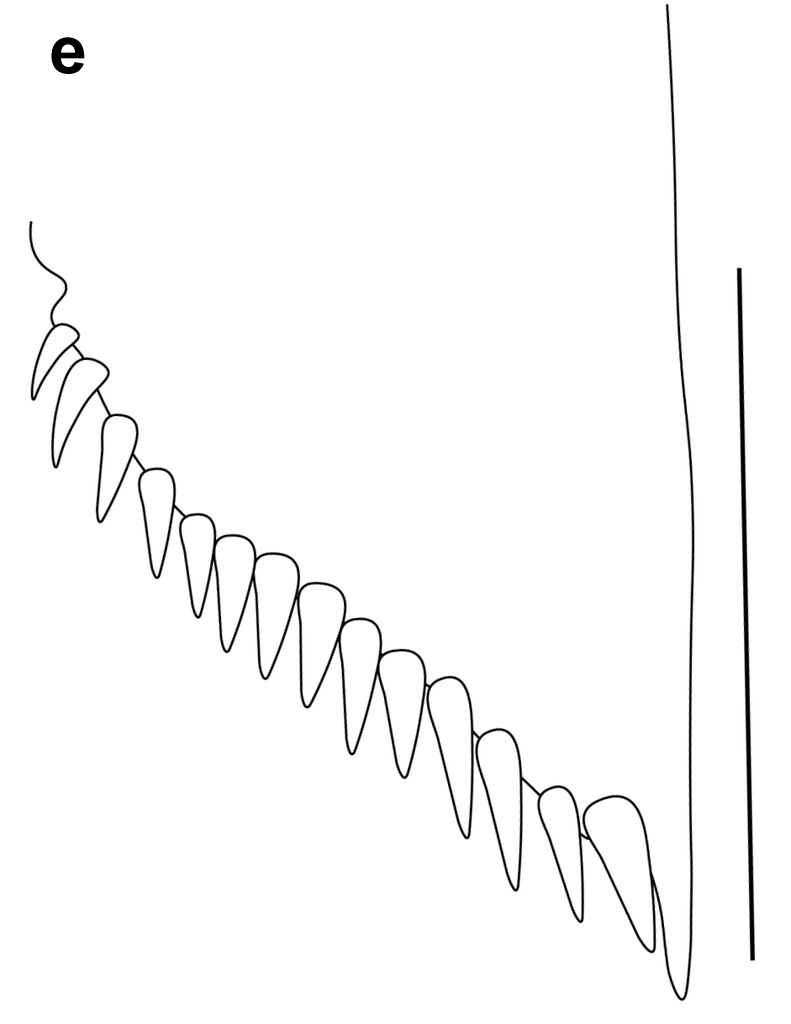
Uropodal diaeresis.

**Figure 11a.**
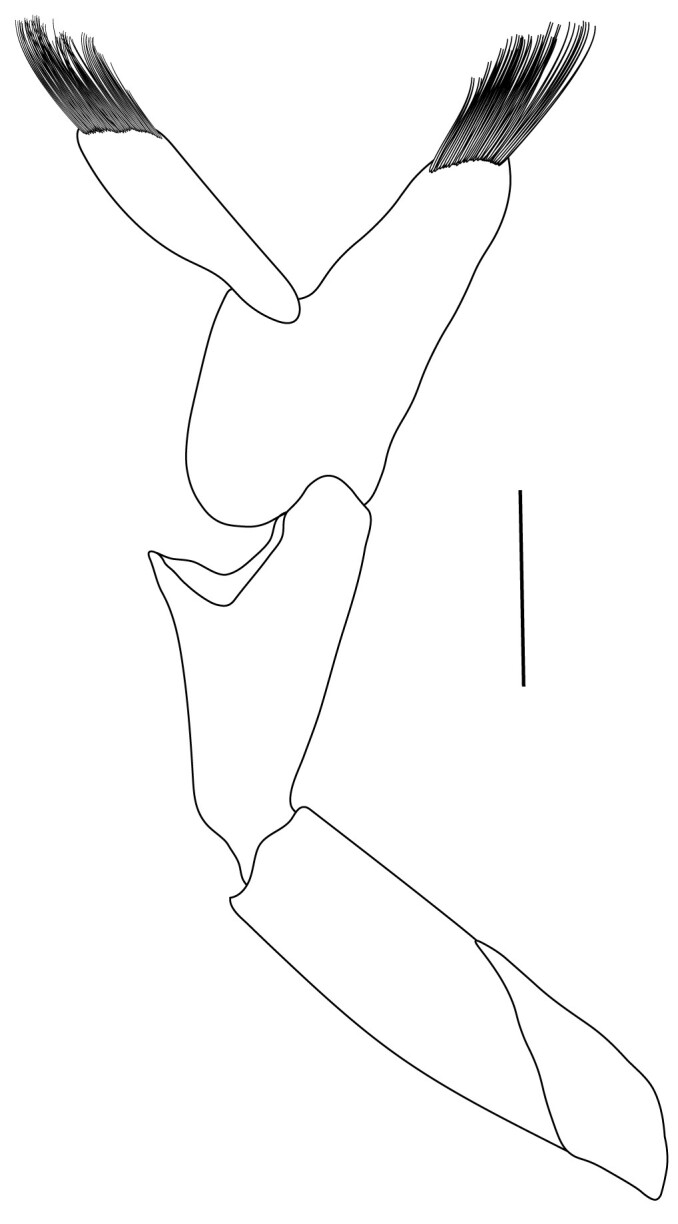
First pereiopod

**Figure 11b.**
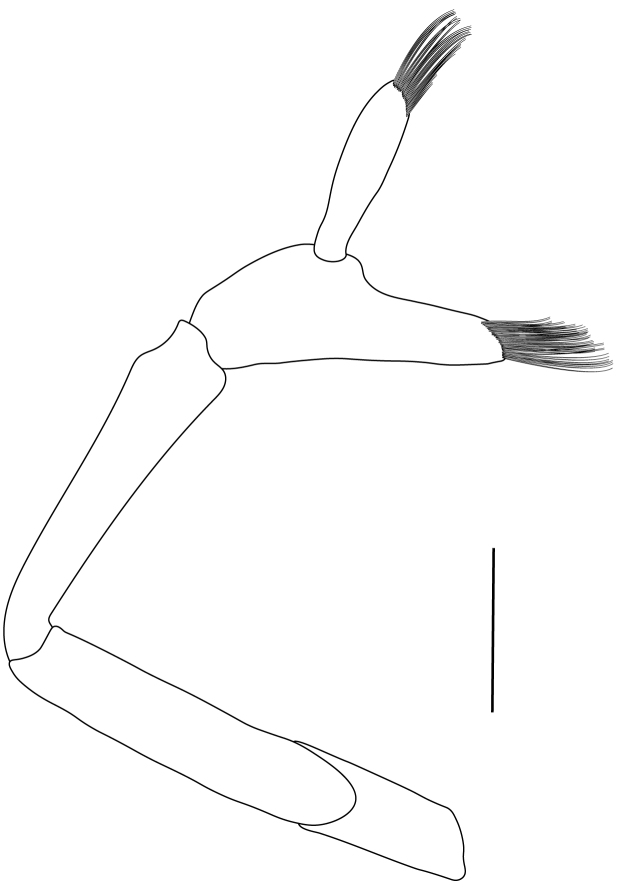
Second pereiopod

**Figure 11c.**
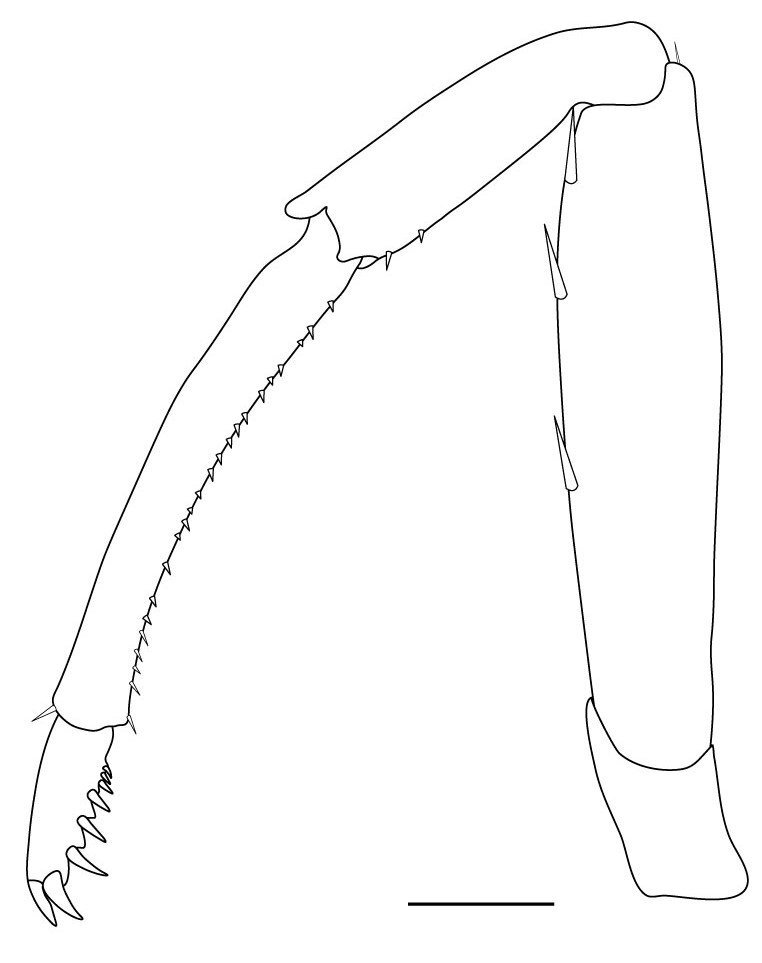
Third pereiopod

**Figure 11d.**
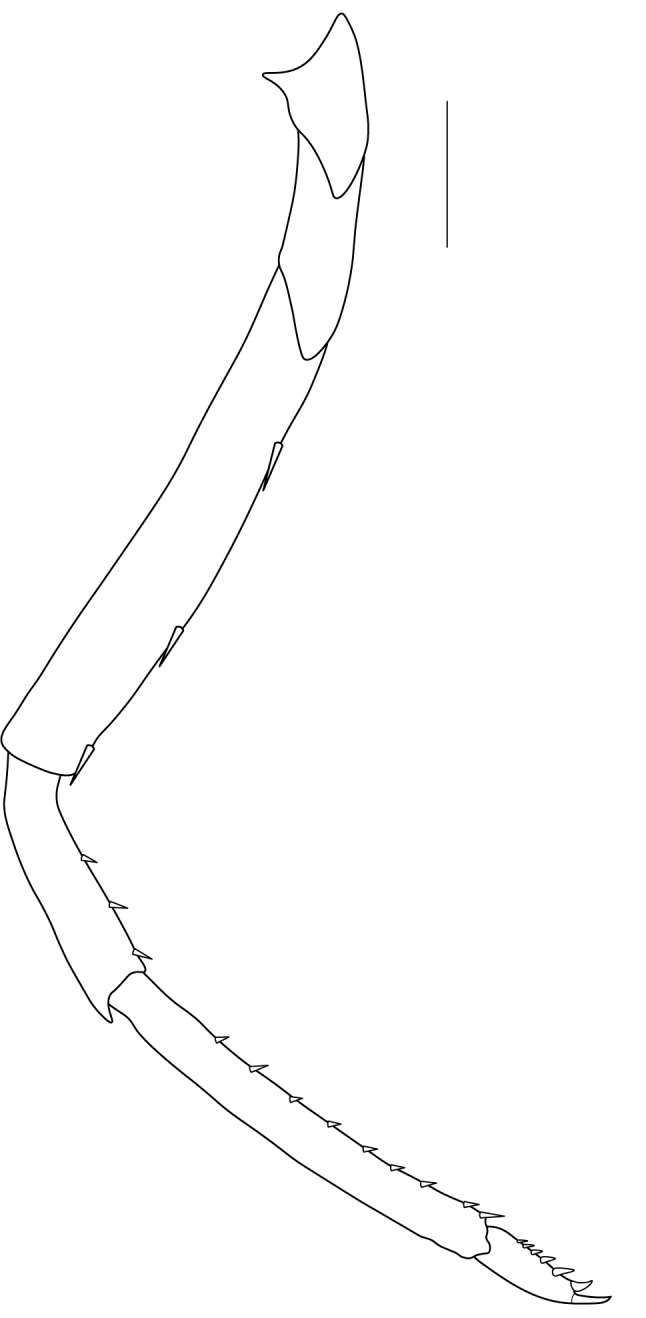
Third pereiopod (female, cl 5.4)

**Figure 11e.**
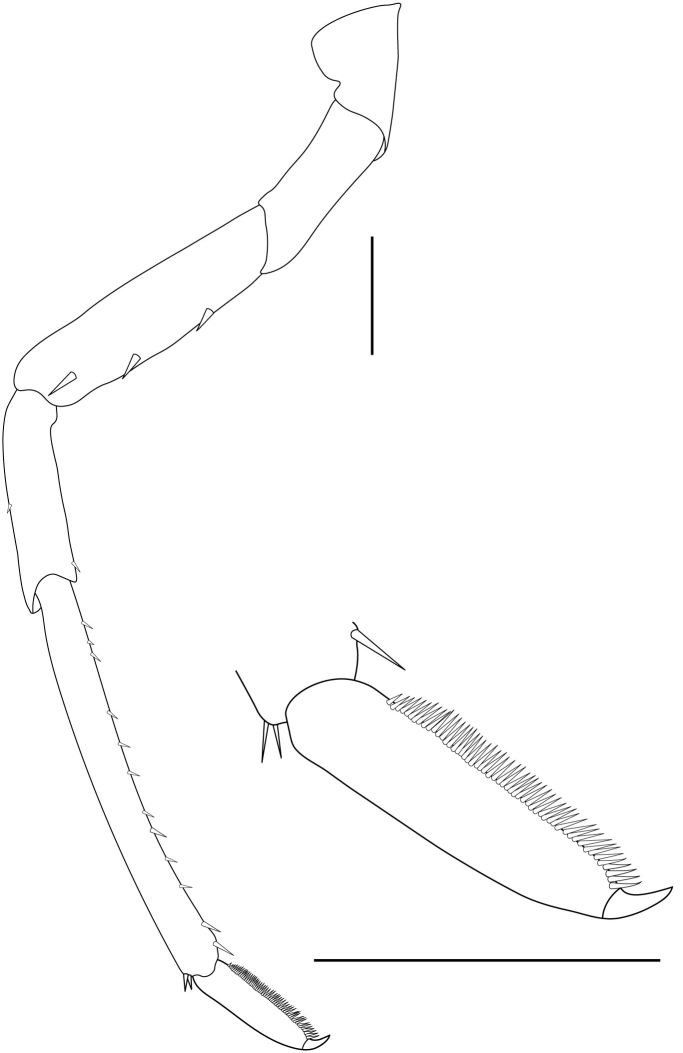
Fifth pereiopod.

**Figure 12a.**
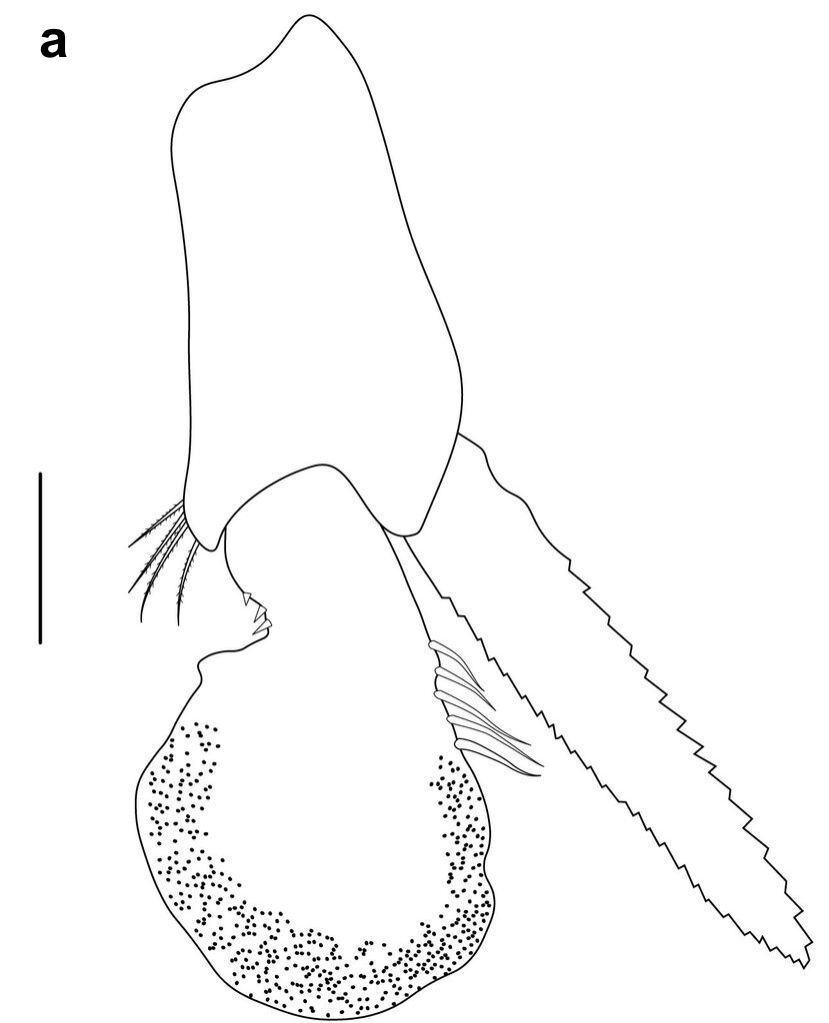
Male first pleopod with appendix interna reduced as a small protrusion at base of inflated part

**Figure 12b.**
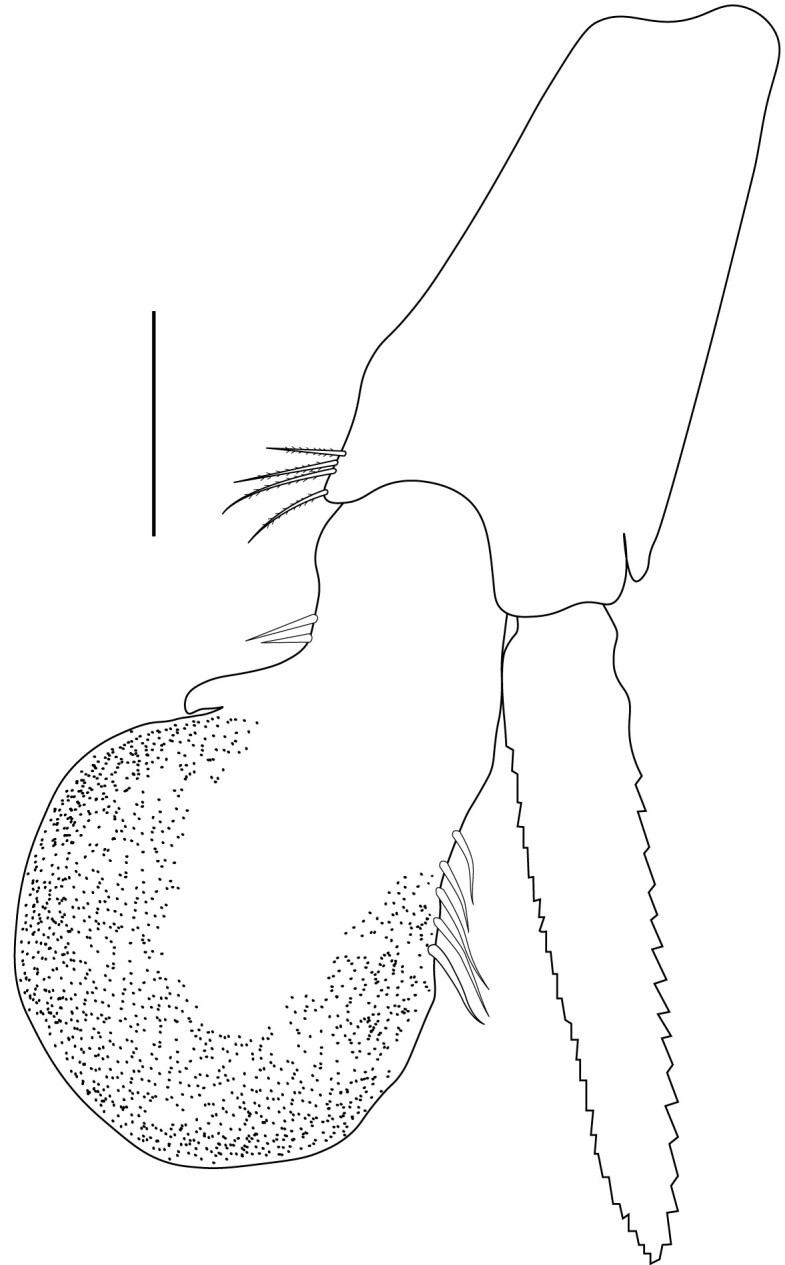
Male first pleopod with appendix interna as short finger at base of inflated part

**Figure 12c.**
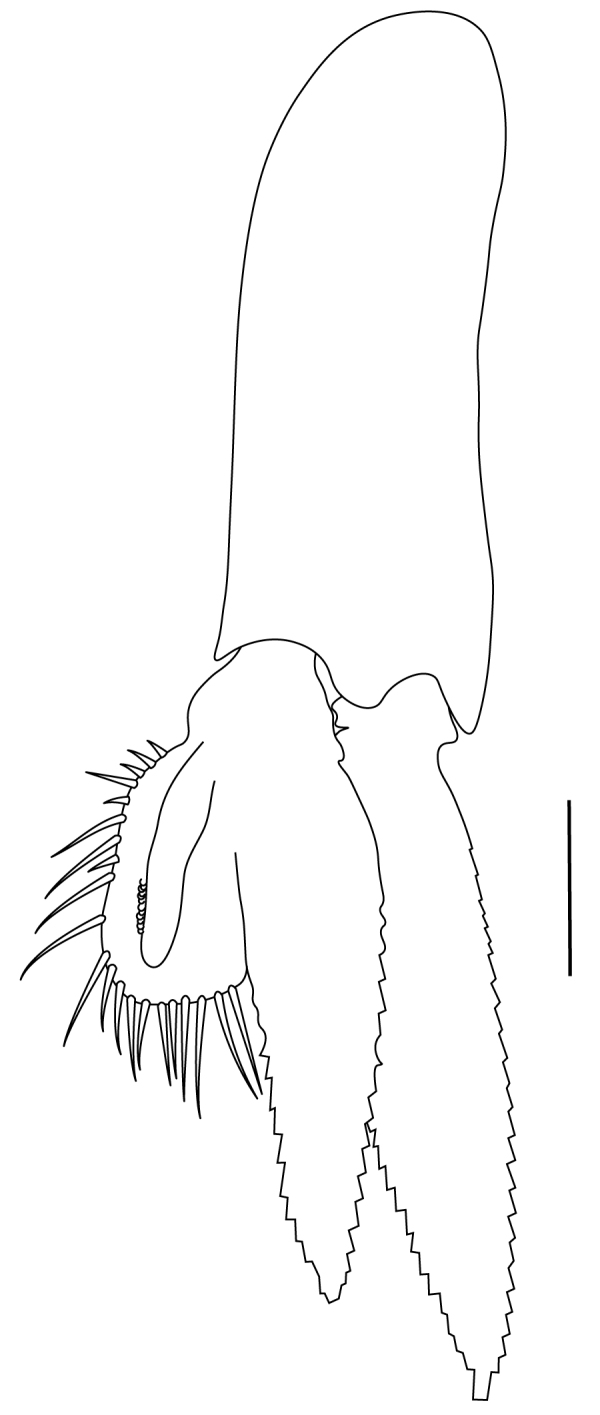
Male second pleopod

**Figure 12d.**
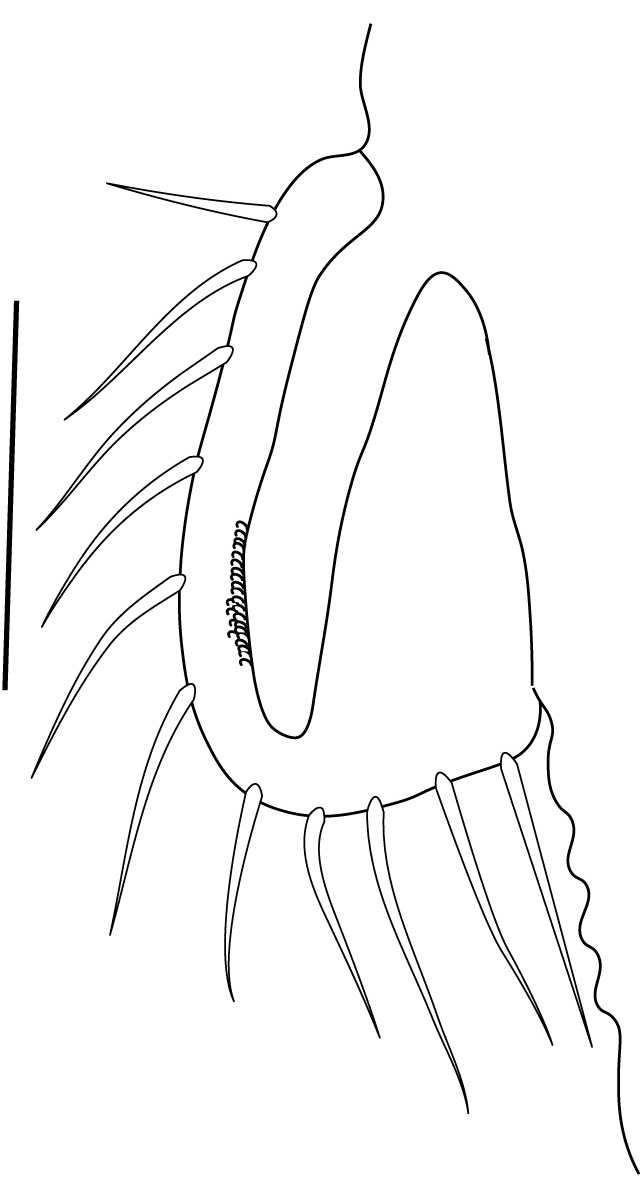
Appendix masculina and interna of male second pleopod.

**Table 1.** The four atyid species with their egg size and distribution in Vietnam.

**Species**	**Egg size (mm)**	**Distribution in Vietnam**
*Caridinacantonensis* Yu, 1938	0.85–0.95 × 0.55–0.6	Only occurs on Cu Lao Cham, a small Island in north-central Vietnam
*Caridinalanceifrons* Yu, 1936	0.8–0.9 × 0.5–0.6	Widely distributed in North Vietnam
*Caridinaserrata* Stimpson, 1860	1.0 × 0.6	Only occurs on Cu Lao Cham Island
*Neocaridinapalmatapalmata* (Shen, 1948)	1.0–1.1 × 0.7	Confined to the northeast of Vietnam
